# Supplementary material for: A Multidisciplinary Curriculum to Standardize Chest Procedures Training for Trainees in General Surgery, Emergency Medicine, and Critical Care
Source: MedEdPORTAL. 2024 Jul 9;20:11421. doi: 10.15766/mep_2374-8265.11421 (PMC11231065; doi:10.15766/mep_2374-8265.11421)
Supplement: Supplementary file 1 — Surgical Tube Thoracostomy Checklist.docxSample Workshop Schedule.docxInstructor Guide Surgical Chest Tube.docxInstructor Guide Seldinger Chest Tube.docxLow-Cost Chest Tube Model.docxInstructor Guide Chest Tube Securement Station.docxInstructor Guide Thoracentesis.docxInstructor Guide POCUS for Thoracic Procedures.docxThoracic Abnormal US Images.pptxChest Procedures Workshop Evaluation.docx [file mep_2374-8265.11421-s001.zip › J. Chest Procedures Workshop Evaluation.docx]

**Example Post-Course Survey**

**Instructions: This form is intended to serve as an example of a post-course survey to be distributed to learners after the workshop to collect feedback on the course.**

**Course Name:** Chest Procedures Workshop **Course Date:**

**Instructor Name(s):**

***Your Division/Department:***

***(optional) Your Name and Training Year***

***===============================================================================***

Please rate the following statements from 1-5, where 1 = strongly disagree 🡪 5 = strongly agree

**Course Evaluation**

1. The course enhanced my understanding of how to perform tube thoracostomy:

Surgical 1..…2…..3…..4…..5…..NA

Seldinger 1..…2…..3…..4…..5…..NA

1. The course enhanced my understanding of how to perform thoracentesis 1..…2…..3…..4…..5…..NA
2. The course enhanced my understanding of how to perform thoracic ultrasound 1..…2…..3…..4…..5…..NA
3. The simulation was an effective educational tool 1..…2…..3…..4…..5…..NA
4. Trainees should spend more time working with simulation to train in chest procedures

1..…2…..3…..4…..5…..NA

1. On a scale of 1-5 (1 = not at all confident, 5 = very confident), please rate your confidence that you could

successfully:

- 1. Place a surgical chest tube if asked to do so: ____ before this workshop

____ after this workshop

- 1. Place a Seldinger-technique chest tube: ____ before this workshop

____ after this workshop

- 1. Perform a thoracentesis: ____ before this workshop

____ after this workshop

- 1. Perform and interpret thoracic ultrasound for procedural planning ____ before this workshop

____ after this workshop

1. Comments:
   _________________________________________________________________________

_________________________________________________________________________

**Simulation Instructor Evaluation**

**Faculty Name: Surgical Chest Tubes**

1. The instructor had a good command of the content 1..…2…..3…..4…..5…..NA
2. The instructor clearly demonstrated the required skills 1..…2…..3…..4…..5…..NA
3. The instructor feedback was helpful 1..…2…..3…..4…..5…..NA
4. Overall, the instructor contributed to my learning 1..…2…..3…..4…..5…..NA
5. Comments:
   ___________________________________________________________________________________

**Faculty Name: Seldinger Chest Tubes**

1. The instructor had a good command of the content 1..…2…..3…..4…..5…..NA
2. The instructor clearly demonstrated the required skills 1..…2…..3…..4…..5…..NA
3. The instructor feedback was helpful 1..…2…..3…..4…..5…..NA
4. Overall, the instructor contributed to my learning 1..…2…..3…..4…..5…..NA
5. Comments:
   ___________________________________________________________________________________

**Faculty Name: Thoracentesis**

1. The instructor had a good command of the content 1..…2…..3…..4…..5…..NA
2. The instructor clearly demonstrated the required skills 1..…2…..3…..4…..5…..NA
3. The instructor feedback was helpful 1..…2…..3…..4…..5…..NA
4. Overall, the instructor contributed to my learning 1..…2…..3…..4…..5…..NA
5. Comments:
   ___________________________________________________________________________________

**Faculty Name: Thoracic Ultrasound**

1. The instructor had a good command of the content 1..…2…..3…..4…..5…..NA
2. The instructor clearly demonstrated the required skills 1..…2…..3…..4…..5…..NA
3. The instructor feedback was helpful 1..…2…..3…..4…..5…..NA
4. Overall, the instructor contributed to my learning 1..…2…..3…..4…..5…..NA
5. Comments:

___________________________________________________________________________________
