# Supplementary material for: A Multidisciplinary Curriculum to Standardize Chest Procedures Training for Trainees in General Surgery, Emergency Medicine, and Critical Care
Source: MedEdPORTAL. 2024 Jul 9;20:11421. doi: 10.15766/mep_2374-8265.11421 (PMC11231065; doi:10.15766/mep_2374-8265.11421)
Supplement: Supplementary file 1 — Surgical Tube Thoracostomy Checklist.docxSample Workshop Schedule.docxInstructor Guide Surgical Chest Tube.docxInstructor Guide Seldinger Chest Tube.docxLow-Cost Chest Tube Model.docxInstructor Guide Chest Tube Securement Station.docxInstructor Guide Thoracentesis.docxInstructor Guide POCUS for Thoracic Procedures.docxThoracic Abnormal US Images.pptxChest Procedures Workshop Evaluation.docx [file mep_2374-8265.11421-s001.zip › A. Surgical Tube Thoracostomy Checklist.docx]

**Tube Thoracostomy Checklist**

**Instructions: This appendix is intended to serve as a rubric to assist in assessment of learners if a checklist-based assessment is preferred for critical actions.**

| **Learner Actions with Descriptions** | **Successful** | **Unsuccessful** |
| --- | --- | --- |
| **Preparation, Draping, and Anesthesia** |  |  |
| Obtains necessary equipment   - *includes chest tube kit/tray, a chest tube between 28 and 36F, antiseptic such as chlorhexidine or betadine, suture (0 or 1-0), and towels or a drape* |  |  |
| Identifies appropriate insertion site   - *between the pectoralis major and latissimus dorsi muscles and above the level of the nipple or inframammary crease* |  |  |
| Prepares Sterile Operating Area   - *preps a wide area with chlorhexidine or betadine and covers surrounding area with sterile towels or a fenestrated drape (learner may discuss appropriate sterile prep if resources are limited)* |  |  |
| Infiltrates Local Anesthetic   - *including both a superficial wheal and along the anticipated course of the chest tube down to the parietal pleura* |  |  |
| **Procedure** |  |  |
| Makes a skin incision   - *incises skin 3-4cm at insertion site in an anterior-posterior orientation, parallel to ribs* |  |  |
| Dissects down to pleura   - *dissects over the rib in a posterior-apical direction from incision* |  |  |
| Penetrates parietal pleura with forceps   - *demonstrates care to not allow instrument to penetrate too deeply into pleura to cause injury to the lung or other underlying tissue* |  |  |
| Spreads forceps in parietal pleura   - *ensures pleural entry site is large enough for the chest tube to pass* |  |  |
| Inserts a finger into the pleural space   - *ensures track is large enough for chest tube and sweeps pleural space* |  |  |
| Places chest tube   - *advances the tube into the pleural space using the forceps, unclamps, and directs the tube in the posterior-apical direction* |  |  |
| Advances the tube an appropriate distance   - *advances tube making sure the distal islet is completely in the pleural space but stopping and predetermined depth or after meeting resistance* |  |  |
| **Securement/Dressing** |  |  |
| Sutures chest tube into position   - *sutures tightly enough to create a slight indentation in the chest tube that prevents movement of the tube but does not impede flow* |  |  |
| Cinches soft tissue tightly around chest tube to avoid air leak   - *can be competed with the same suture that secures the tube or with a second suture* |  |  |
| Covers insertion site   - uses sterile gauze over site and secures with tape or other adhesive wrap |  |  |
| Connects the chest tube to the drainage system   - *connects the chest tube to the drainage system tightly and secures connection with tape* |  |  |
