# Supplementary material for: A Multidisciplinary Curriculum to Standardize Chest Procedures Training for Trainees in General Surgery, Emergency Medicine, and Critical Care
Source: MedEdPORTAL. 2024 Jul 9;20:11421. doi: 10.15766/mep_2374-8265.11421 (PMC11231065; doi:10.15766/mep_2374-8265.11421)
Supplement: Supplementary file 1 — Surgical Tube Thoracostomy Checklist.docxSample Workshop Schedule.docxInstructor Guide Surgical Chest Tube.docxInstructor Guide Seldinger Chest Tube.docxLow-Cost Chest Tube Model.docxInstructor Guide Chest Tube Securement Station.docxInstructor Guide Thoracentesis.docxInstructor Guide POCUS for Thoracic Procedures.docxThoracic Abnormal US Images.pptxChest Procedures Workshop Evaluation.docx [file mep_2374-8265.11421-s001.zip › B. Sample Workshop Schedule.docx]

**Chest Procedures Workshop**

**Sample Schedule for Large Group**

**Instructions: This sample schedule is provided as a reference for faculty and staff when planning the workshop.**

**Learners**

25 total, divided into 5 groups of 5

Ideally grouped by experience level via pre-course email or during initial 30 minutes of the workshop

**Instructors**

1.

2.

3.

4.

5.

**Ultrasound Models**

1.

2.

**Schedule**

0800-0830 Introduction, didactic if needed

0830-1030 Hands-on time

1030-1040 Break/catchup time

1040-1225 Hands-on time

| Station | Instructor | 0830-0915 | 0915-1000 | 1000-1045 | Break | 1055-1140 | 1140-1225 |
| --- | --- | --- | --- | --- | --- | --- | --- |
| Surgical Tubes |  | 1 | 5 | 4 |  | 3 | 2 |
| Tube Thoracostomy Securement and Troubleshooting |  | 2 | 1 | 5 |  | 4 | 3 |
| Seldinger Tubes |  | 3 | 2 | 1 |  | 5 | 4 |
| Thoracentesis |  | 4 | 3 | 2 |  | 1 | 5 |
| Ultrasound |  | 5 | 4 | 3 |  | 2 | 1 |
