# Supplementary material for: A Multidisciplinary Curriculum to Standardize Chest Procedures Training for Trainees in General Surgery, Emergency Medicine, and Critical Care
Source: MedEdPORTAL. 2024 Jul 9;20:11421. doi: 10.15766/mep_2374-8265.11421 (PMC11231065; doi:10.15766/mep_2374-8265.11421)
Supplement: Supplementary file 1 — Surgical Tube Thoracostomy Checklist.docxSample Workshop Schedule.docxInstructor Guide Surgical Chest Tube.docxInstructor Guide Seldinger Chest Tube.docxLow-Cost Chest Tube Model.docxInstructor Guide Chest Tube Securement Station.docxInstructor Guide Thoracentesis.docxInstructor Guide POCUS for Thoracic Procedures.docxThoracic Abnormal US Images.pptxChest Procedures Workshop Evaluation.docx [file mep_2374-8265.11421-s001.zip › C. Instructor Guide Surgical Chest Tube.docx]

**Surgical Tube Thoracostomy**

**Instructions: This instructor guide is to be used as a reference by faculty guiding the surgical chest tube placement station. It outlines the supplies needed, station setup, methods of instruction, steps of the procedure, common errors by trainees, assessment of trainees, and provides a clinical scenario through which to discuss the procedure in a clinical context.**

**
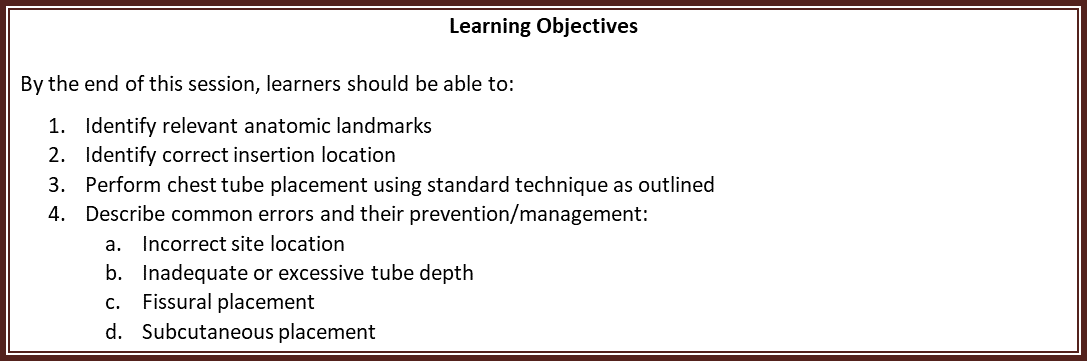
**

**Supplies**

- Manikin (e.g., Laerdal TraumaMan)
- Two equipment tables
- Gauze (1 box)
- Chest tube trays including scalpel, curved forceps, needle driver, scissors
- Chest tubes of various sizes
- 10 cc syringes
- 25 and 22 gauge needles
- Local anesthetic of choice, we use 1% Lidocaine (10cc vials, may be simulated)

Consider starting the session with the clinical case provided at the end of this document.

**Station Setup**

- The manikin should be placed on a table with both sides of the chest accessible to trainees.
- One full set of needed equipment on a table on both sides of the manikin allows for simultaneous practice by two trainees.


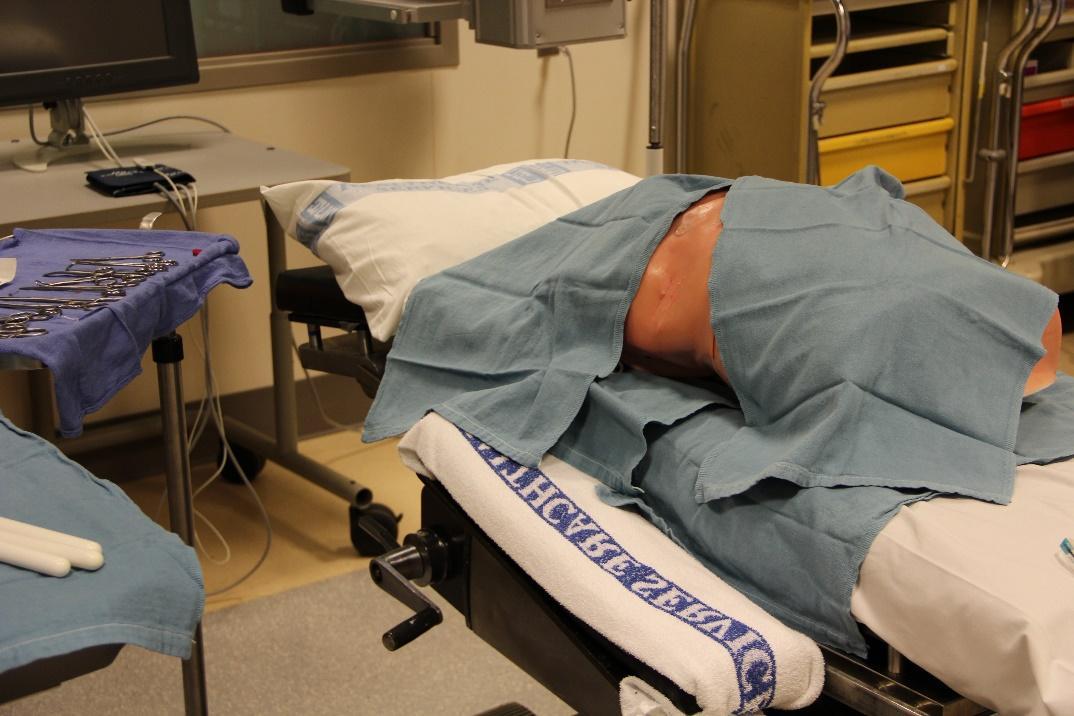

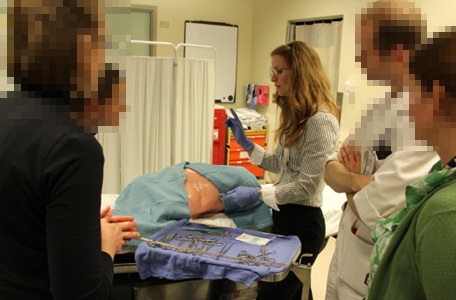


Figure 1a (left). Torso manikin draped for tube thoracostomy placement, with surgical instruments on tray.

Figure 1b. (right) Faculty (author AEM) demonstrates the procedure to a group of learners. Others in image obscured for privacy.

*Images author owned (AEM, Washington Institute for Simulation in Healthcare (WISH), Seattle, Washington)*

**Prerequisites**

Prior to the workshop learners will be asked to watch a video on chest tube placement utilizing images from a published chest tube performance video^^[[1]](#footnote-1)^^. Alternatively, instructors may choose to present the didactic material in person at the beginning of the course, using their own slides and videos.

*Required background knowledge*

- Understanding of anatomy and physiology of the chest wall, pleura, and lungs
- Clinical indications for tube thoracostomy

*Required background skills expected in trainees prior to receiving training in the target course:*

- Learners should have essential skills concerning:
  - How to create a sterile field
  - How to administer local anesthetic
  - How to perform skin incisions and blunt dissection

**Step 1: Expert Description (5 minutes)**

1. **Preparation**

Prior to demonstrating placement, instructor should discuss:

- 1. Optimal patient positioning, including arm restraints
  2. The importance of patient comfort including the following options:
     1. Moderate sedation
     2. Topical analgesia using generous amounts of local anesthetic
     3. Regional nerve blocks
     4. Procedural sedation and analgesia are beyond the scope of this curriculum, but learners should be aware of these options
  3. Key anatomic structures
  4. Optimal insertion location within the Triangle of Safety
  5. Discussion of relative contraindications to urgent/non-emergent chest tube placement including:
     1. INR >1.5 (<https://www.ncbi.nlm.nih.gov/pmc/articles/PMC6026252/>)
     2. PLT <50 (<https://www.ncbi.nlm.nih.gov/pmc/articles/PMC6142536/>)

1. **Equipment**

Prior to demonstrating placement, instructor should also briefly review key equipment including:

- 1. Chest tube highlighting:
     1. Size differences and clinical selection of appropriate tube
        - We start with a 28 French sized chest tube but discuss rationale for selecting larger tube sizes (complex effusions, hemothorax, trauma).
        - We recommend using whatever chest tube size/sizes is most available and comfortable to faculty.
     2. Drainage holes and the importance of ensuring all are intra-thoracic
  2. Clamps of different shapes and sizes, with advantages and drawbacks of each

The drainage collection system will be discussed at the Tube Thoracostomy Securement and Troubleshooting station and should not be discussed here.

**Step 2: Expert Demonstration (5 minutes)**

Instructor should demonstrate performance of tube thoracostomy with narration of key technique elements, per the procedural steps below.

**Step 3: Learner Hands-on Practice (35 minutes)**

- Learners can practice on both sides of the chest simultaneously
- If more than 4 learners are present, a second manikin and ideally a second instructor should be used to ensure adequate hands-on time to reach adequate performance during a 45-minute rotation
- The instructor should avoid repeating the expert demonstration if possible, using verbal instruction to guide learners through difficult steps in order to maximize hands-on time

**Step 4: Assessment**

- In most workshops, competent performance is determined by the instructor using informal global assessment during the course of the station
- Successful performance includes the following elements:
  - Positioning and draping (verbalized/pantomimed)
  - Anesthesia (verbalized, pantomimed)
  - Insertion steps listed below
- If a learner is unable to perform all steps of the procedure to the satisfaction of the instructor by the end of the station rotation, the instructor must discuss with the workshop director the need for additional training at a later date

**Surgical Chest Tube Insertion Steps**

1. Preparation

Discuss indications of the procedure with the attending. Address duration of placement and plans for removal.

- 1. Notify nursing staff of the procedure.
  2. Ensure that all equipment is present and easily accessible from the patient’s bedside.
  3. Obtain informed consent.
  4. Patient should be placed on nasal oxygen and continuous pulse oximetry.
  5. Verify patient identity and procedure location during “Time Out.”

1. Positioning and draping

Position the patient: for anterior axillary line insertion, the head of the bed should be elevated 30-60 degrees, and the arm on the procedural side is restrained over the patient’s head.

Locate and describe the appropriate landmarks and indicate the site of tube placement verbally. Depth of insertion can be estimated by placing the tube tip near the clavicle and marking the distance to the insertion site with a small clamp, ensuring the last drainage hole is within the pleural space.

Prep a wide sterile field from the head to below the torso of the simulator.

1. Anesthesia and Anxiolysis

In nonemergent cases, patients should receive procedural sedation and/or parenteral analgesia; regional nerve block may be considered. Again, a detailed discussion of these topics is beyond the scope of this curriculum.

Local anesthesia: attach a 25 G needle to a 5 or 10 cc syringe containing local anesthetic.

Create a skin wheal (pantomime) at the insertion site, directly over the rib.

Withdraw the needle and replace with 22 G needle. Infiltrate local anesthetic (pantomime on mannequin) into subcutaneous fat, fascia, and deep tissues. Generously anesthetize the anticipated track of the chest tube, over the top of the rib at the inferior border of rib space to be entered. Using this needle, confirm presence of air or fluid in the pleural space.

*Note: If using 1% lidocaine the maximum lidocaine dose: 5-7mg/kg with epinephrine (recommended) or 3-5mg/kg without epinephrine.

1. Procedure

Incise the skin at the insertion site in an anterior-posterior orientation. An incision of 3-4cm is adequate in the nontrauma setting.

Using curved forceps, dissect down to the parietal pleura in a postero-superior direction. Penetrate the subcutaneous tissues and then spread the forceps, arching over the top of the rib and through the intercostal space.

The parietal pleura will be penetrated with forceful application of the forceps and a palpable popping sensation. Spread the forceps wide to ensure the pleural entry site is large enough for the chest tube to pass easily.

Insert a finger into the pleural space. Ensure that the track is large enough for the chest tube to pass and sweep in all directions of the pleural space to ensure the absence of adhesions.

Insert the chest tube into the pleural space using curved forceps clamped near the end of the tube to help guide placement. Advance the tube in a postero-superior direction to the previously marked depth, or until pain or resistance is felt, then pull back 2-3 cm. Ensure the tube is inserted past the last drainage hole.

1. Securing the tube and cleaning up*

Secure the chest tube at the insertion site with 0 or 1-0 silk by placing a U-stitch around the incision site and wrapping the suture several times around the chest tube before knotting several times. Tube should be fixed sufficiently tightly to prevent accidental removal but not so tight as to impair tube drainage.

Cover the insertion site with sterile gauze and foam tape.

Connect chest tube to closed drainage system. Secure connection points with foam tape.

Clean off patient and secure redundant tubing.

Dispose of sharps properly.

Document procedure protocol in patient’s chart.

Communication with patient and family, attending, nursing staff and provide appropriate signout to primary medical team.

* Section V. “Securing and Cleaning up” is included in procedural steps for completeness, but this section is covered at the Tube Thoracostomy Securement and Troubleshooting station.

**Common Errors and Remedies**

Incorrect site location

Identify the triangle of safety prior to draping

Use ultrasound to confirm diaphragm location

In emergent situations or if ultrasound is not available, avoid placement below the 5th intercostal space to avoid subdiaphragmatic placement

Excessive tube depth

Before insertion, measure the tube length on the chest from insertion site to clavicle to estimate maximal depth

If discomfort occurs, consider excessively deep placement causing mediastinal irritation

Inadequate tube depth or subcutaneous placement

Be cautious if there is excessive adipose tissue

Keep your finger inside of the chest cavity during insertion if possible so you can feel the tube slide along your finger and over the rib into the cavity. Avoid placing the tube without this tactile guidance

Note location of islets and depth of subcutaneous tissue with palpation, and ensure last islet is inserted far enough to enter the pleura

Chest tube kinking

Spin the tube gently during insertion and prior to concluding the procedure to ensure there is no resistance

Fissural placement

Sweep the chest cavity with your finger to identify adhesions that may direct the tube into the fissure

Guide the chest tube posterior and superior during insertion

Start the incision over or below the rib and tunnel slightly superiorly over the rib to give the tube a natural cranial/superior path

**Clinical Case**

***CC: Fever and shortness of breath***

**HPI:** A 67-year-old man presents to the ED with fever, cough productive of purulent sputum and progressive shortness of breath over the last 3 days. He endorses right sided pleuritic chest discomfort. He does not have any chronic respiratory diagnoses but has a 40 pack-year smoking history.

**All:** None

**Meds:** lisinopril, aspirin, metformin

**PMHx**: HTN, type II diabetes mellitus

**SocHx**: Married, retired construction supervisor. Born and raised near Seattle, no significant travel history. Moderate EtOH use, tob as above, no other drug use.

**FamHx:** Father died of MI in his 80s, mother had HTN and diabetes, died of a stroke in her 70s

**ROS:** complete ROS negative except as per HPI

***Pertinent PE:***

**Vitals:** Temp 38.9 HR 114, BP 90/60, RR 24, O2 90% on room air

**Gen:** Mild obesity, looks acutely ill. Increased work of breathing but is able to speak in short sentences

**CV:** Tachycardic, no murmur

**Chest:** Decreased breath sounds 1/2 up the right posterior chest wall with dullness to percussion over the same area. Coarse rales and egophony audible in the right mid-lung.

**Abd:** Soft, nontender, normal bowel sounds

**Ext/Skin:** Warm with no edema or rash.

**Thoracic ultrasound:** Large complex pleural effusion on the right. In some areas it appears the lung may be adherent to the chest wall.


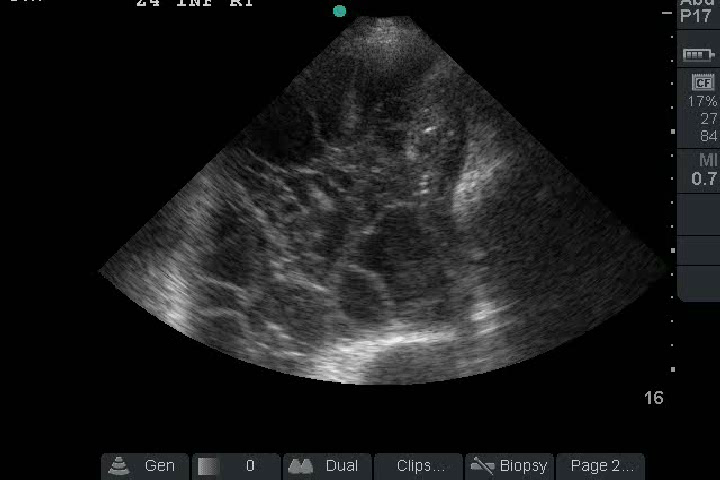


*Image author owned (AEM)*

**A note on variations in scope of practice:** Instructors should discuss with learners the role of specialty involvement in decision making and performance of tube thoracostomy as appropriate based on available resources. In different institutions, this procedure may be performed by a provider from emergency medicine, general surgery, thoracic surgery, pulmonary or interventional pulmonary medicine depending on local norms and available resources. If learners belong to a specialty that would not typically perform this procedure at their institution, faculty should acknowledge that trainees may go on to practice in another location where they would be expected to perform surgical tube thoracostomy.

1. Dev SP, Nascimiento B Jr, Simone C, Chien V. Videos in clinical medicine. Chest-tube insertion. N Engl J Med. 2007;357(15):e15. doi:10.1056/NEJMvcm071974 [↑](#footnote-ref-1)
