# Supplementary material for: A Multidisciplinary Curriculum to Standardize Chest Procedures Training for Trainees in General Surgery, Emergency Medicine, and Critical Care
Source: MedEdPORTAL. 2024 Jul 9;20:11421. doi: 10.15766/mep_2374-8265.11421 (PMC11231065; doi:10.15766/mep_2374-8265.11421)
Supplement: Supplementary file 1 — Surgical Tube Thoracostomy Checklist.docxSample Workshop Schedule.docxInstructor Guide Surgical Chest Tube.docxInstructor Guide Seldinger Chest Tube.docxLow-Cost Chest Tube Model.docxInstructor Guide Chest Tube Securement Station.docxInstructor Guide Thoracentesis.docxInstructor Guide POCUS for Thoracic Procedures.docxThoracic Abnormal US Images.pptxChest Procedures Workshop Evaluation.docx [file mep_2374-8265.11421-s001.zip › F. Instructor Guide Chest Tube Securement Station.docx]

**Tube Thoracostomy Securement and Troubleshooting**

**Instructions: This instructor guide is to be used as a reference by faculty guiding the tube thoracostomy securement station. It outlines the supplies needed, station setup, methods of instruction, steps of the procedure, common errors by trainees, and assessment of trainees.**

**
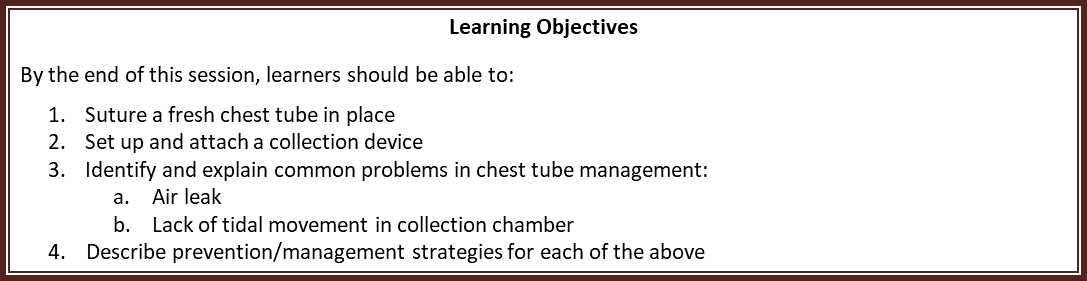
**

**Supplies**

- Manikin: any model that allows suturing
- Two each:
  - Chest tube
  - Equipment table
  - Suture equipment: needle driver, scissors, suture
  - Gauze
  - Foam tape
  - Silk tape
  - Chest tube drainage system (e.g. Atrium device)

**Station Setup**

- The manikin should be placed on a table with both sides of the chest accessible to trainees.
- One full set of needed equipment on a table on each side of the manikin allows for simultaneous practice by two trainees.

**Prerequisites**

Chest tube securement is best reviewed after the tube insertion station. However, for large workshops some learners may need to attend this station prior to insertion. Typically, instructors will address all necessary content at the station itself using demonstration and discussion.

*Required background knowledge*

- Understanding of anatomy and physiology of the chest wall, pleura, and lungs

*Required background skills expected in trainees prior to receiving training in the target course:*

- Learners should have essential suture technique skills

**Step 1: Expert Description (10 minutes)**

1. **Preparation**

Prior to demonstrating placement, instructor should discuss key principles of tube securement:

1. Importance of secure suture technique to prevent dislodgement
2. Equipment and technique options (see below)
3. **Equipment**

Prior to demonstrating placement, instructor should also briefly review key equipment:

- 1. 0 or 1-0 silk suture
  2. Tape options for skin and tubing connections
  3. Drainage system setup, including filling the water seal chamber and adjusting suction
  4. Proper methods to connect tubing from patient to collection device to wall suction

**Step 2: Expert Demonstration (5 minutes)**

Alternatively, the instructor may verbally lead learners through the steps of securement and collection device setup.

**Step 3: Learner Hands-on Practice (25 minutes)**

- Learners can practice on both sides of the chest simultaneously
- If more than 4 learners are present, additional manikins should be used
- The instructor should avoid repeating expert performance if possible, using verbal instruction to guide learners through difficult steps in order to maximize hands-on time

**Step 4: Troubleshooting**

- Once learners have demonstrated adequate securement technique, the instructor should lead the group through common troubleshooting scenarios as outlined below
- Learners should demonstrate systematic evaluation of the chest tube system from the insertion site to the wall suction device, describing key elements and locations of evaluation

**Step 5: Assessment**

- In most workshops, competent performance is determined by the instructor using informal global assessment during the course of the station
- Successful performance must include all elements of the steps outlined below:
  - Suture chest tube insertion site
  - Connect all tubing from patient to drainage device to wall suction
  - Verbal discussion of troubleshooting steps
- If learners are unable to perform all steps of the procedure to the satisfaction of the instructor by the end of the station rotation, the instructor must discuss with the workshop director the need for additional training at a later date.

**Chest Tube Securement Steps**

1. Preparation: collect/arrange equipment
2. Suturing

**U-Stitch (See Example Photos Below)**

Using a large silk suture (1-0 or 0) and taking care not to puncture the chest tube with the suture needle,

- 1. Place a U-stitch (horizontal mattress) around the tube

1. Cinch down to create a tight seal around the tube and the skin/soft tissue
2. Pass both tails of suture around the chest tube multiple times from both sides
3. Loops around the tube may remain as seen in image 5 (“Roman sandal” configuration) or slid down toward the incision as seen in image 6. before cinching down with a knot that creates a small, non-obstructive indent in the tube


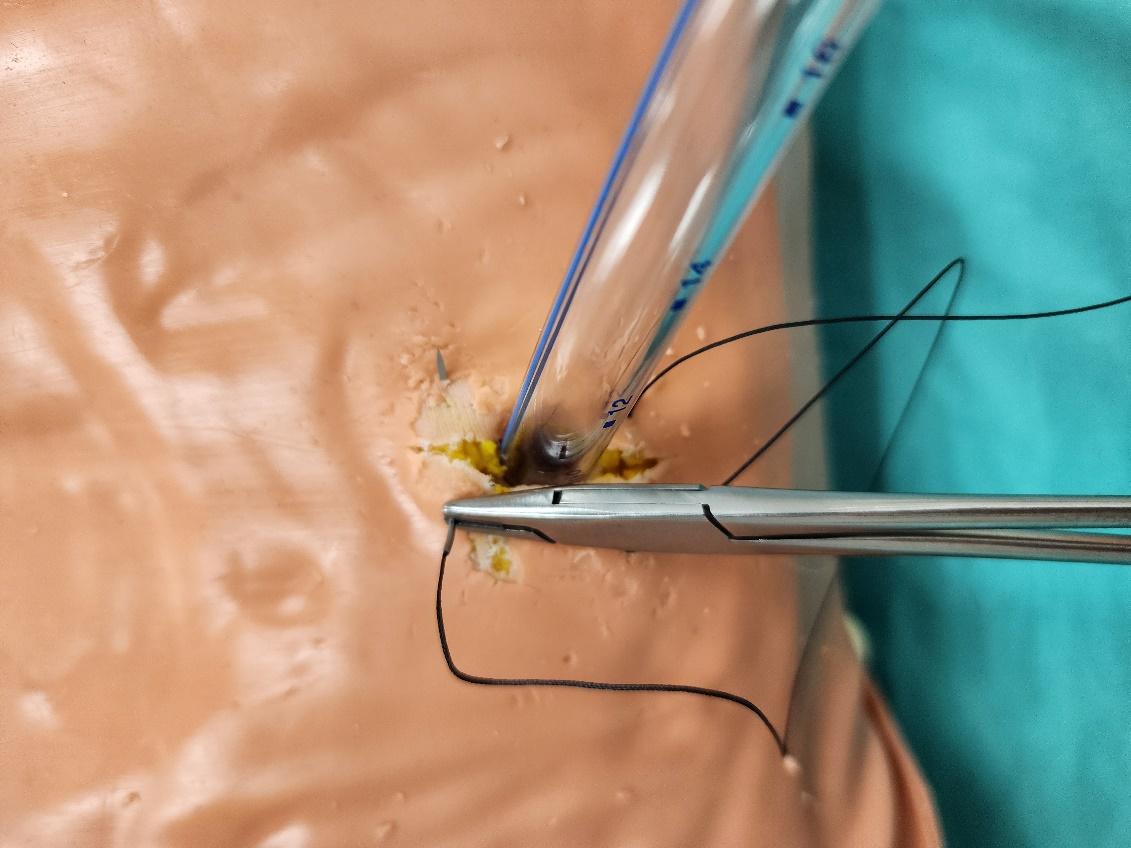

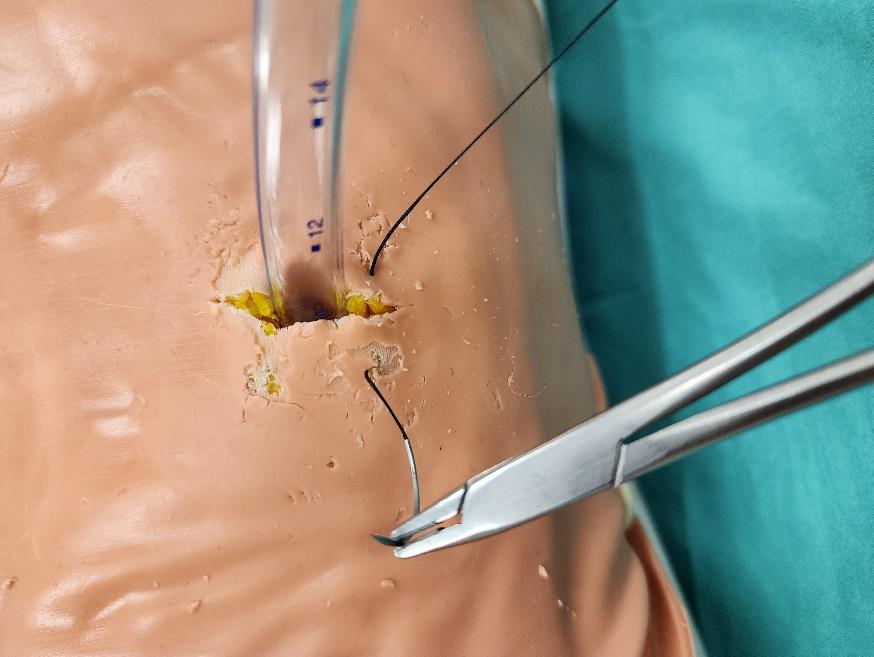


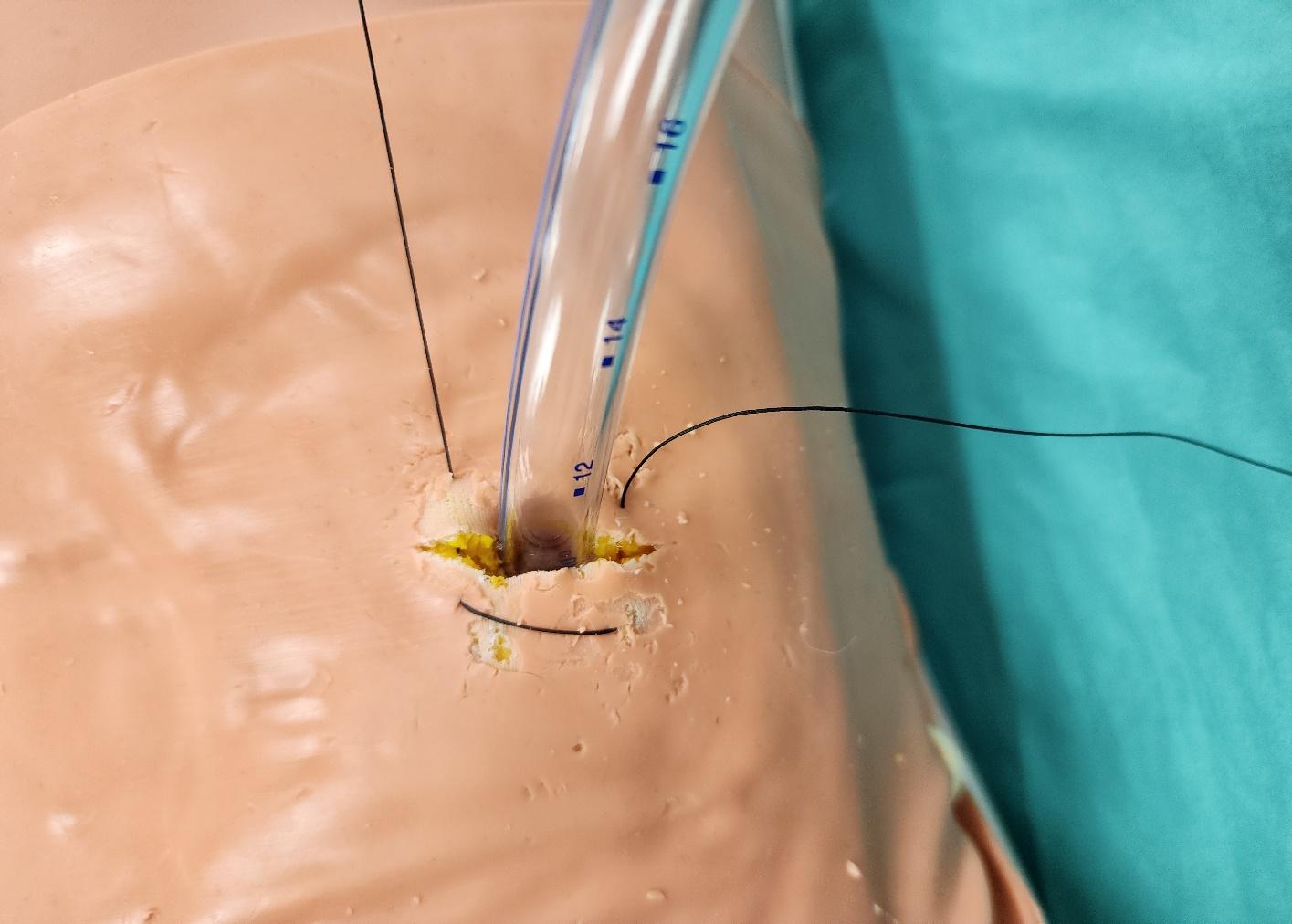


**



**
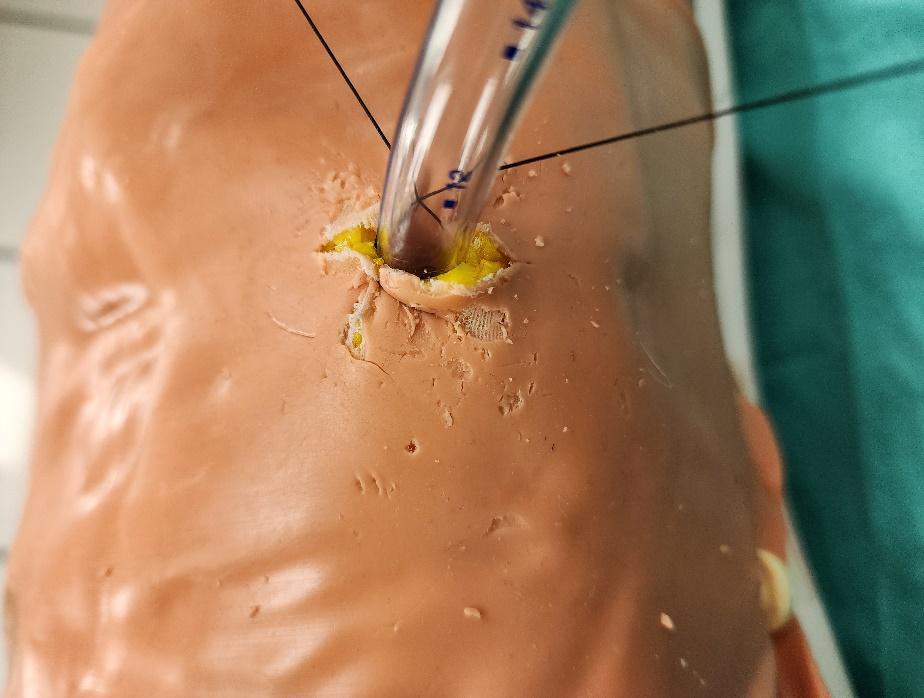

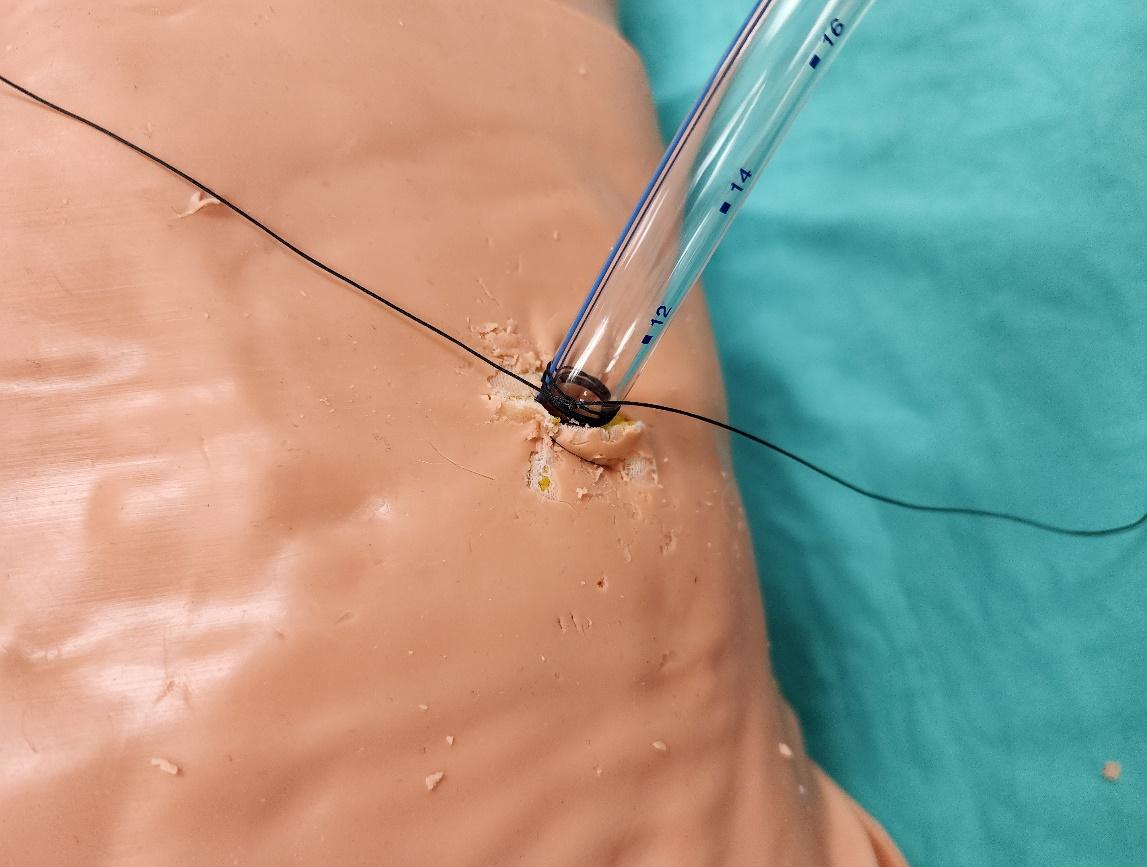

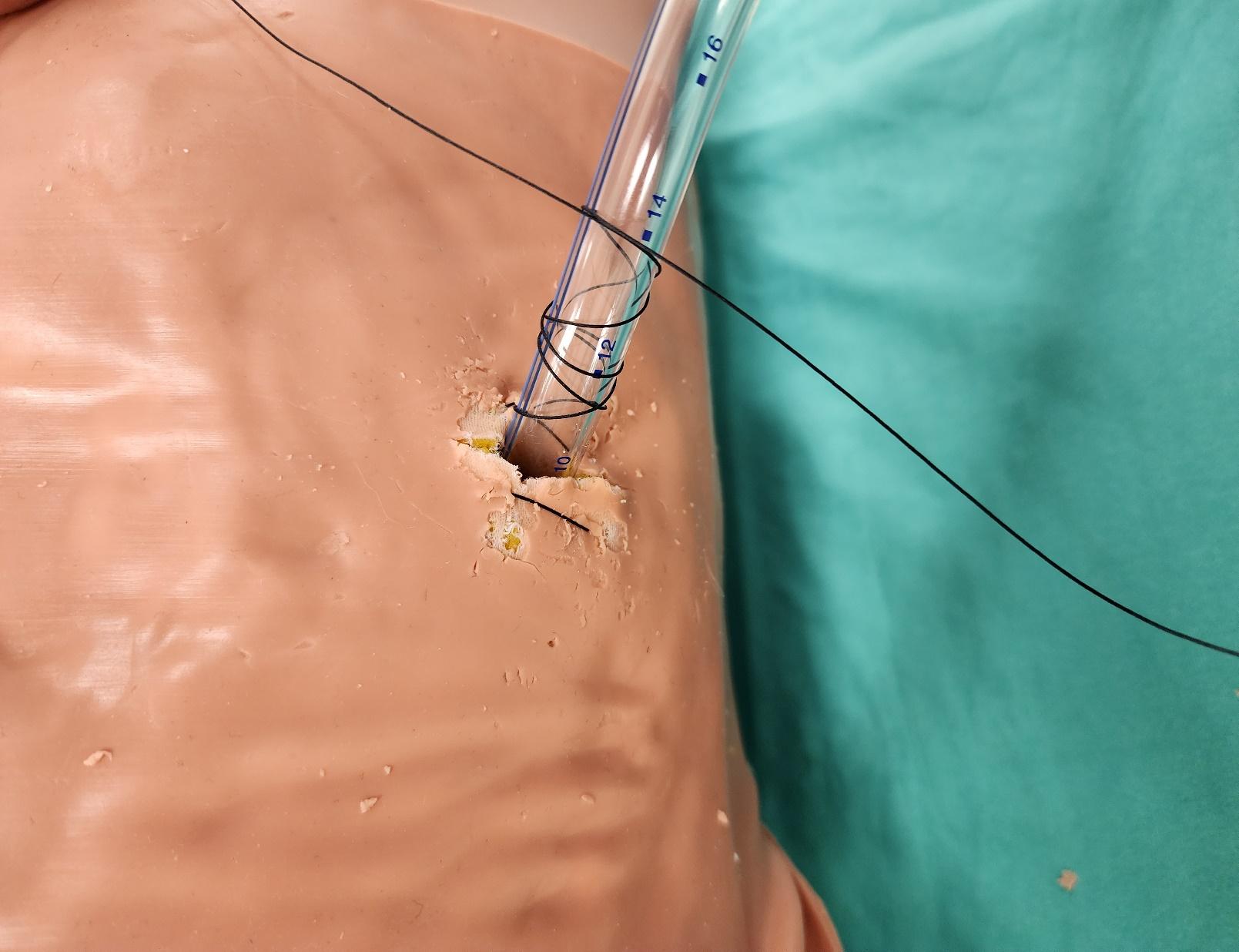


1. Cover the insertion site with gauze drain sponge, then foam tape to secure.
2. Chest tube drainage system setup
   1. Fill water seal chamber
   2. The chest tube itself is connected to tubing running to the atria using a spiral technique that enables continuous visualization of tubing connections.


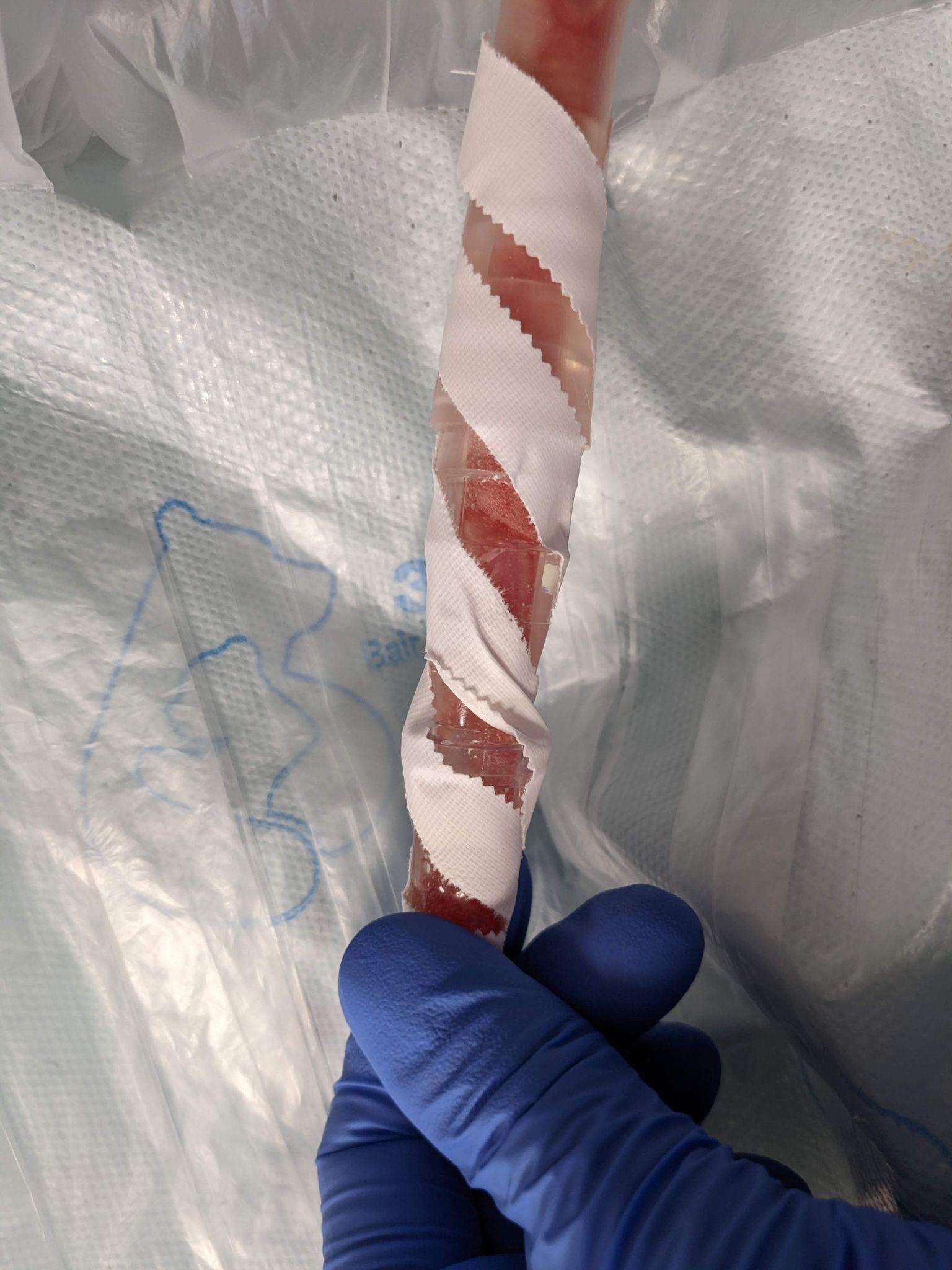

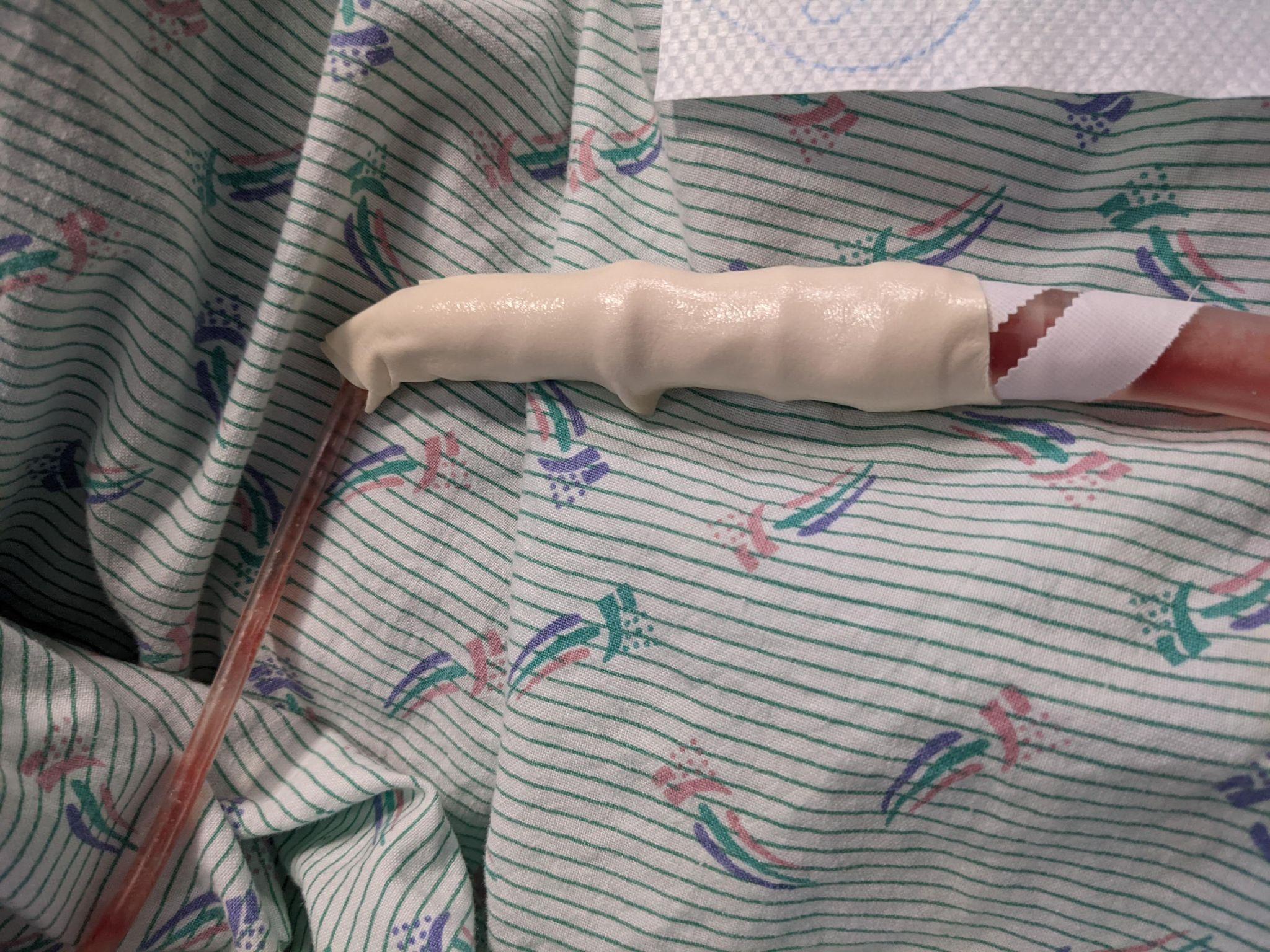


Fig 1a. (left) This tube connection site demonstrates spiral technique, allowing visualization of the connection location.

Fig 1b. (right) Foam tape has been placed over the spiral tape, a common mistake that does not prevent separation at the connection site, and will impair its detection.

*Images author owned (AEM)*

1. Cleaning up
   1. Clean off patient and secure redundant tubing
   2. Dispose of sharps properly
   3. Document procedure protocol in patient’s chart
   4. Communication with patient and family, attending, nursing staff and provide appropriate signout to primary medical team

**Common Pitfalls/Troubleshooting**

This component of the station is focused not on learner error, but common problems that arise with chest tubes following insertion.

Chest tube dislodgement (Prevention)

Avoid using suture smaller than 0 or 1-0 suture

Use a U stitch at the skin and loop the suture around the tube multiple times. A “sandal” suture pattern is optional

Secure tightly to the tube, but not so much that there is obstruction of the tube

Use multiple large pieces of tape over the insertion site to reinforce suture in the event the chest tube is accidentally pulled, e.g. during patient movement

Disconnection between chest tube and atrium (Prevention)

Secure the connection point between chest tube and atrium while maintaining visibility (see photo above)

Air leak

Identify an air leak as indicated by bubbles present in the water seal chamber of the atrium

Differential diagnosis: the origin of an air leak may be

Intrathoracic (e.g. bronchopleural fistula)

At the insertion site

Elsewhere in the tubing system

Troubleshooting: with suction on, determine the location of the air leak by sequentially pinching or clamping the tubing along the length of the system

| Clamp location | Leak location suggested by cessation of bubbles |
| --- | --- |
| Chest tube itself | Internal (e.g. bronchopleural fistula) |
|  | Insertion site (e.g. inadequate chest tube insertion depth): Undress the insertion site to inspect |
| Tubing between chest tube and atrium | Connection between chest tube and atrium tubing |
|  | Damaged proximal atrium tubing |
| (Bubbles persist despite the above) | Connection between atrium tubing and collection chamber |

Lack of tidal movement in collection chamber

Occurs when intrathoracic pressure variations are not transmitted to the chest tube system

Assess when suction is off

Differential diagnosis:

Tubing is kinked (may be internal or external to the patient) or clamped

Tubing is obstructed e.g. by thick exudative material or coagulated blood

Intrathoracic pressure is not changing on the affected side e.g. endotracheal tube is misplaced

Troubleshooting:

Visually inspect external tubing for kinking or clamp

Evaluate CXR for internal tube migration

“Stripping” or “milking” the tubing to encourage flow is controversial because it may cause strong negative pressure at the open (internal) end of the chest tube, affecting nearby tissues

If clogging with blood or thick drainage is suspected, the tube may be flushed with sterile saline. Technique varies by size of tube (e.g. small bore tube with three-way stopcock vs larger surgical tube requiring system interruption) and can be addressed if time at instructor discretion
