# Supplementary material for: A Multidisciplinary Curriculum to Standardize Chest Procedures Training for Trainees in General Surgery, Emergency Medicine, and Critical Care
Source: MedEdPORTAL. 2024 Jul 9;20:11421. doi: 10.15766/mep_2374-8265.11421 (PMC11231065; doi:10.15766/mep_2374-8265.11421)
Supplement: Supplementary file 1 — Surgical Tube Thoracostomy Checklist.docxSample Workshop Schedule.docxInstructor Guide Surgical Chest Tube.docxInstructor Guide Seldinger Chest Tube.docxLow-Cost Chest Tube Model.docxInstructor Guide Chest Tube Securement Station.docxInstructor Guide Thoracentesis.docxInstructor Guide POCUS for Thoracic Procedures.docxThoracic Abnormal US Images.pptxChest Procedures Workshop Evaluation.docx [file mep_2374-8265.11421-s001.zip › G. Instructor Guide Thoracentesis.docx]

**Thoracentesis**

**Instructions: This instructor guide is to be used as a reference by faculty guiding the thoracentesis station. It outlines the supplies needed, station setup, methods of instruction, steps of the procedure, common errors by trainees, assessment of trainees, and provides a clinical scenario through which to discuss the procedure in a clinical context.**

**
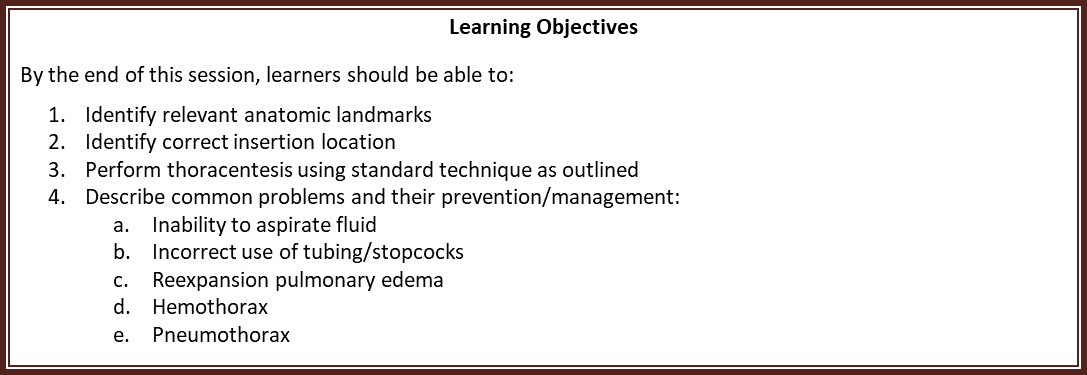
**

**Supplies**

- Thoracic simulator(s) (e.g., Simulab Thoracentesis model THMP-30)
  - Minimum one per two learners
- Two equipment tables with stools/chairs for learners
- Extra towels
- Thoracentesis kits one per simulator
  - The hub of the thoracentesis catheter in many commercially available kits contain a spring and ball bearing that prevents reinsertion of the trocar once removed from the catheter. The ball bearing can be removed by drilling a hole into the side of the catheter hub, allowing instructors to replace the trocar for multiple learners to use the kit.

*
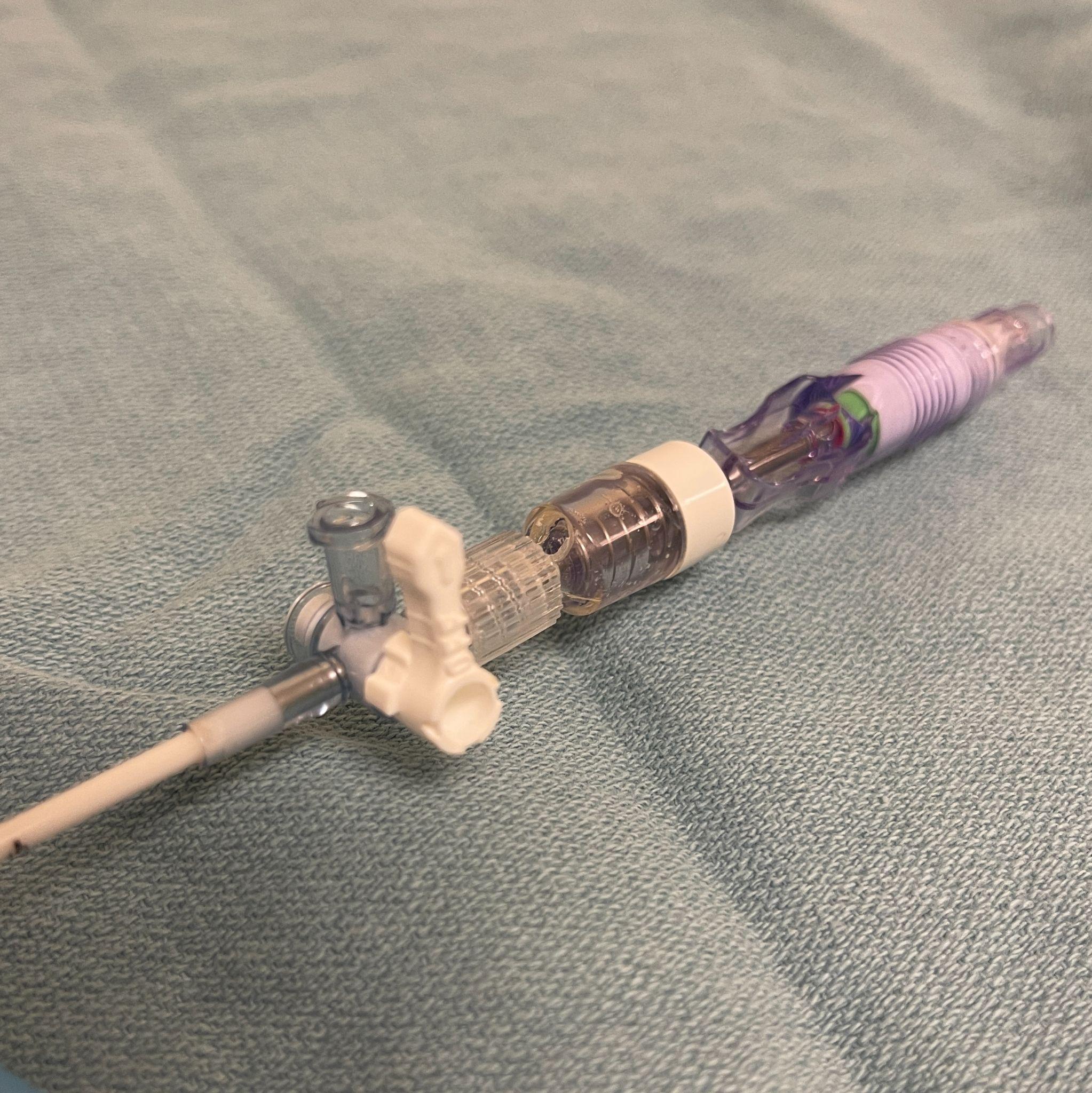
*

Figure 1. Modified thoracentesis trocar. Spring is visible but ball bearing has been removed to facilitate multiple uses. *Image author owned (AEM)*

Consider starting the session with the clinical case provided at the end of this document.

**Station Setup**

- The simulator should be placed on a table at a comfortable height for seated or standing performance
- Multiple simulators allow simultaneous practice by trainees.
- Table should be large enough for at least one open kit next to each simulator
- One ultrasound machine per simulator, if ultrasound-compatible


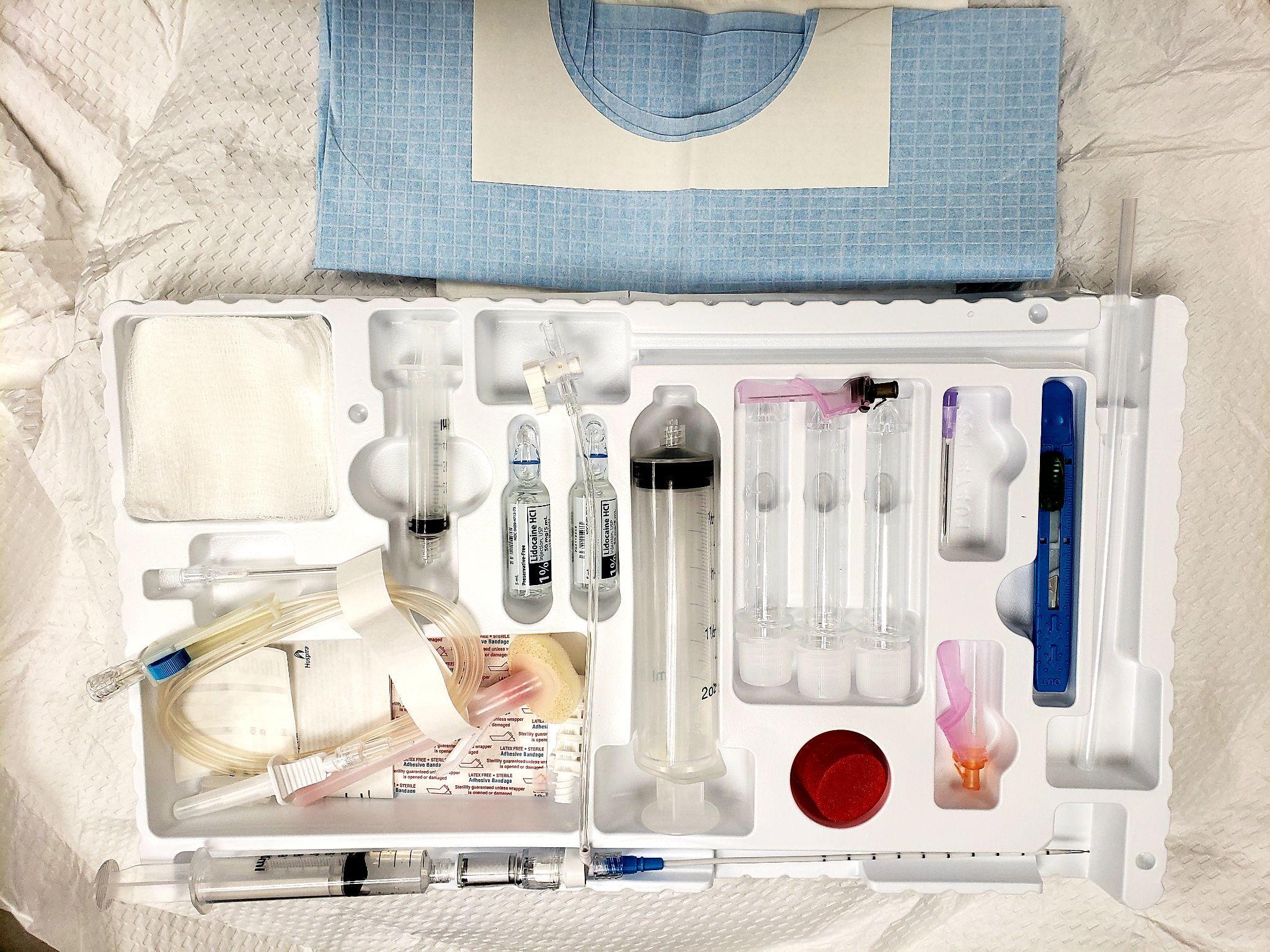


Figure 2. Example of a thoracentesis kit widely available in the United States. *Image author owned (RJK)*

**Prerequisites**

Preparatory materials are not required prior to the workshop.

*Required background knowledge*

- Understanding of anatomy and physiology of the chest wall, pleura, and lungs
- Clinical indications for thoracentesis

*Required background skills expected in trainees prior to receiving training in the target course:*

- Learners should have essential skills concerning:
  - How to create a sterile field
  - How to administer local anesthetic

**Step 1: Expert Description (5 minutes)**

1. **Preparation**

Prior to demonstrating placement, instructor should discuss:

1. Optimal patient positioning, including patients who are unable to sit up for the procedure
2. The importance of patient comfort including the following options:
   1. Topical analgesia using generous amounts of local anesthetic such as lidocaine (max. 3-5mg/kg without epinephrine, or 5-7mg/kg with epinephrine)
3. Key anatomic structures
4. Optimal insertion location using surface landmarks and confirmed by ultrasound
5. **Equipment**

Prior to demonstrating placement, instructor should also briefly review key equipment including:

- 1. Spring loaded needle tip that extends beyond catheter
  2. Color change associated with spring tip and exposure of cutting surface (if present in the kit being used for this workshop)
  3. Catheter length relative to needle length, requiring additional insertion depth once fluid is obtained prior to advancing catheter through pleura
  4. Tubing connections and stopcocks

**Step 2: Expert Demonstration (5 minutes- optional)**

Instructors may perform thoracentesis with narration of key technique elements, per the procedural steps below. This step is optional especially for advanced learner groups who have performed large numbers of thoracenteses earlier in their training. Alternatively, instructors may choose to verbally guide learners through an initial placement, followed by independent practice.

**Step 3: Learner Hands-on Practice (35 minutes)**

- Learners can practice on multiple simulators simultaneously
- If more than 2 learners are practicing at one time, ideally a second instructor should be used to ensure adequate feedback to each learner
- The instructor should avoid repeating expert performance if possible, using verbal instruction to guide learners through difficult steps in order to maximize hands-on time

**Step 4: Assessment**

- In most workshops, competent performance is determined by the instructor using informal global assessment during the course of the station
- Successful performance must include all elements of the steps outlined below:
  - Positioning and draping (verbalized/pantomimed)
  - Anesthesia (verbalized, pantomimed)
  - Procedure
- If learners are unable to perform all steps of the procedure to the satisfaction of the instructor by the end of the station rotation, the instructor must discuss with the workshop director the need for additional training at a later date.

**Thoracentesis Steps**

1. Preparation

Discuss indications, contraindications, coagulation labs, and ultrasound findings

Notify nursing staff of the procedure

Obtain informed consent

Ensure all equipment is present and easily accessible from patient’s bedside:

Sterile gloves (some settings require additional sterile garb)

Thoracentesis kit

Additional items if kit does not contain, or per operator preference: Chlorhexidine, local anesthetic/lidocaine, larger sterile drape, or additional sterile towels

Ultrasound machine with sector array transducer

Verify patient identity and procedure location during “Time Out”

Discussion of relative contraindications to urgent/non-emergent chest tube placement including:

1. INR >1.5 (<https://www.ncbi.nlm.nih.gov/pmc/articles/PMC6026252/>)
2. PLT >50 (<https://www.ncbi.nlm.nih.gov/pmc/articles/PMC6142536/>)
3. Positioning and landmarks

Patient seated with feet supported, arms resting on tray table, leaning forward only slightly to avoid fluid pooling anteriorly. Bed height adjusted so operator can sit or stand comfortably

Locate and describe external landmarks: midscapular line, above the 8^th^ or 9^th^ rib

Ultrasound confirmation: * Take note of ultrasound transducer angle

Confirm adequate fluid to avoid lung and diaphragm; mark site by indentation not ink

Estimate subcutaneous thickness = anticipated insertion distance to fluid aspiration

Consider addition of color doppler to avoid large vessels at the insertion site

Confirm sliding lung at the apex of the thorax

1. Prepare operating area

Don mask and sterile gloves

Chlorhexidine scrub over a wide area around insertion site

Secure sterile drape +/- additional towels per operator preference

Open thoracentesis tray and lay out equipment on sterile field or bedside table within easy reach during the procedure

1. Anesthesia

Create a skin wheal at the insertion site with local anesthetic (we use 1% lidocaine) using a 25G or 22G needle

Using a 22G or 19G needle, infiltrate local anesthetic along the anticipated catheter track over the top of the rib. Maintain the angle of the ultrasound transducer above, and use a stepwise approach (advance while aspirating – confirm no blood – inject)

When pleural fluid is reached, note needle depth. Withdraw slightly to the point at which fluid is no longer aspirated, and deliver additional 1cc to optimize pleural anesthesia

1. Procedure

Incise the skin at the insertion site using the scalpel in the kit. 5mm is usually adequate

Insert the over-the-needle catheter and syringe in the thoracentesis kit into the small incision made in the skin. An additional 2-3cc of lidocaine allows for just-in-time anesthesia if needed

Advance along the anesthetized track, aspirating as the catheter and needle are advanced together. If using a spring-loaded needle tip, note the color change in the needle hub when passing through soft tissue (usually red as shown below)

Once pleural space is reached, fluid will aspirate into the syringe and color indicator will change to green or clear. Advance the catheter and needle together an additional 1cm to ensure the catheter has entered the pleural space

Hold the needle still and advance the catheter to its fullest extent into the pleural cavity (advancing only the catheter, not the needle)

Once the catheter is fully inserted, remove the needle. Ensure stopcock is closed to the patient

For diagnostic procedures, use a syringe to aspirate enough pleural fluid to send all required studies. For therapeutic thoracentesis or cytology, attach drainage tubing and bag and collect fluid using “pumping” action with syringe and stopcock. Maximum 1500 cc, or until the patient develops significant discomfort

When drainage is complete, ask the patient to hum to avoid a breath in that could entrain air and cause pneumothorax, then quickly remove the catheter


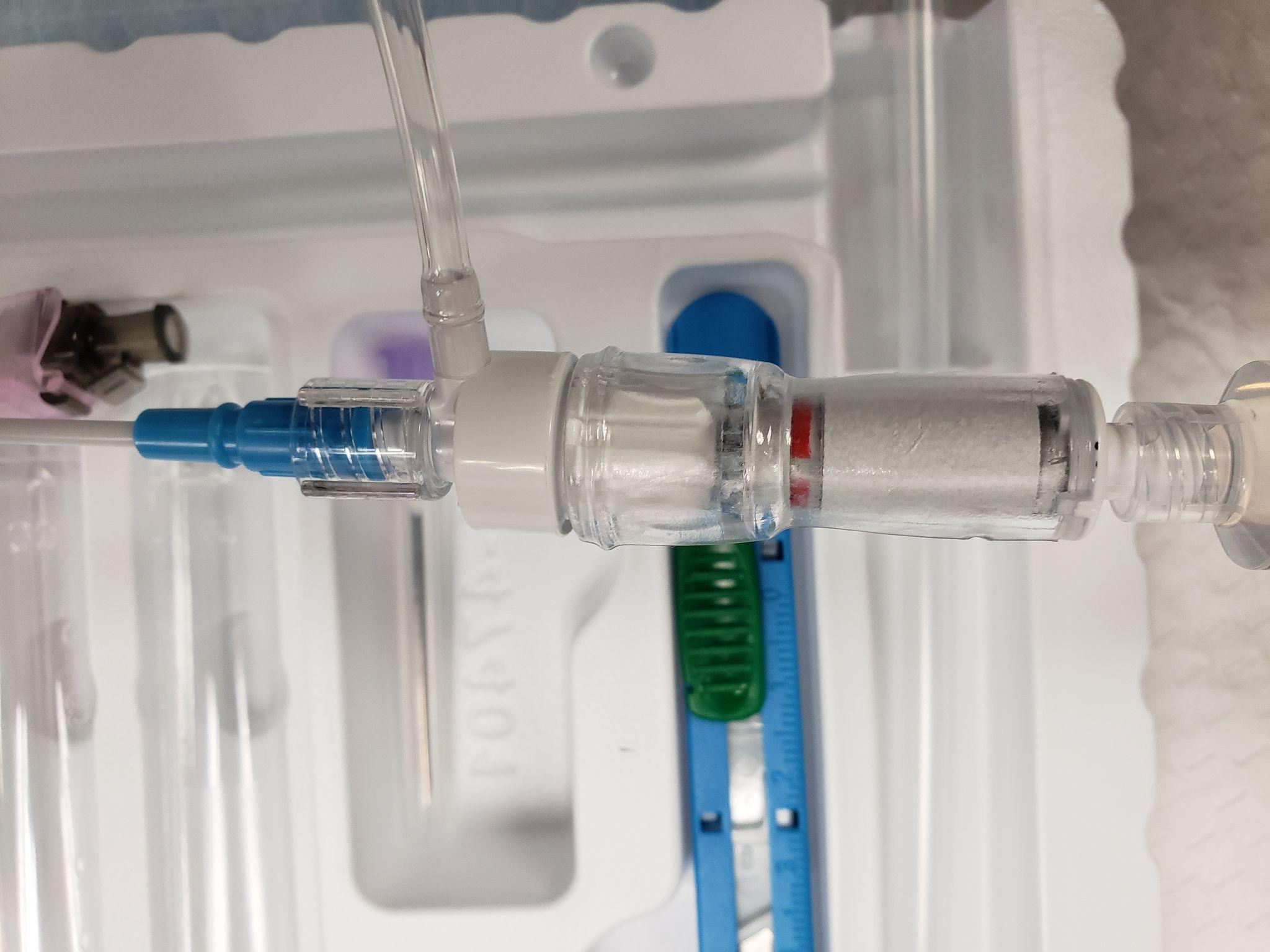

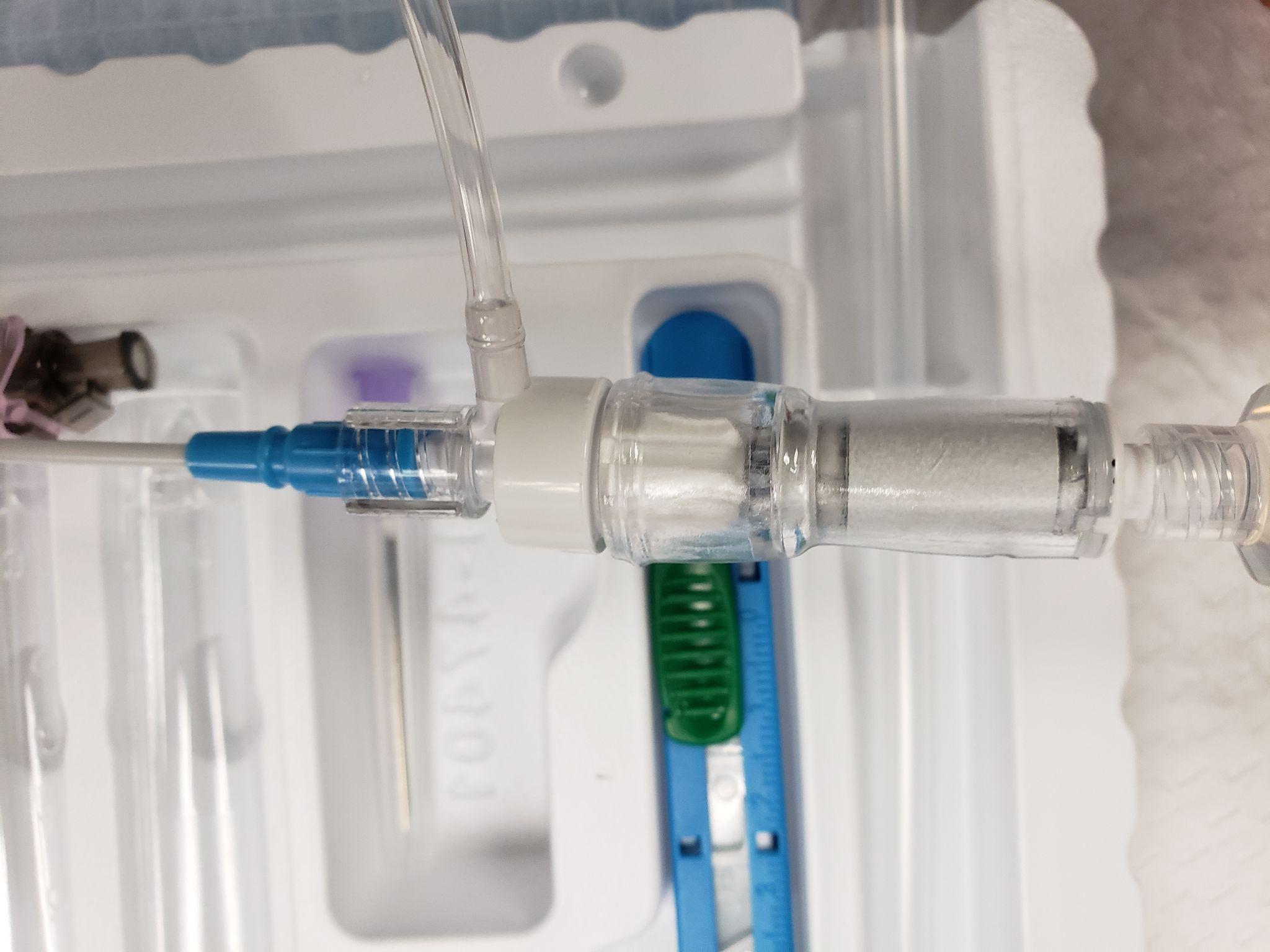


Figure 3a. (left) Catheter and needle demonstrating red indicator while passing through soft tissue.

Figure 3b. (right) This indicator is absent or green when the needle is not encountering resistance, such as when the needle has passed through the pleura to enter the pleural space. *Images author owned (AEM)*

1. Cleaning up

Place bandage or gauze and tape over procedure site

If present pre-procedure, confirm sliding lung sign is still present at lung apex

Place sharps in appropriate containers

Document procedure in the chart and provide appropriate signout to primary medical team

**Common Problems and Prevention/Remedy**

Unable to aspirate fluid during catheter placement

Ensure patient is not leaning too far forward, which will cause fluid to sit anteriorly in the chest, inaccessible to posterior catheter

Ensure patient positioning is unchanged from ultrasound to procedure. If needed, use a sterile probe cover to reassess ultrasound image prior to inserting catheter

Insert the catheter at the same angle as the ultrasound transducer, ensuring the catheter is directed toward the visualized fluid

After fluid is aspirated during insertion, advance the needle-catheter an additional 1 cm to ensure the catheter tip (which is shorter than the needle) enters the pleural space

Fluid aspirated into catheter, but unable to drain via tubing into collection bag

Confirm three-way stopcock is correctly oriented: “open” to patient and tubing

Confirm tubing is correctly assembled with respect to one-way valves

Replace tubing with a syringe to aspirate directly. If successful, tubing may be incorrectly assembled or faulty. If unsuccessful, inject 1-2cc sterile saline and assess ease of instillation

If saline flows easily but aspiration is still unsuccessful, catheter eyeholes may be obstructed by surrounding tissue (e.g., reexpanding lung) or thick exudate within the pleural fluid:

Reevaluate catheter positioning within the pleural fluid using ultrasound

Rotate catheter at the skin or withdraw slightly to move away from obstruction

Re-expansion pulmonary edema

Most likely a cytokine-mediated response to excessively negative intrapleural pressure rather than a consequence of too large or too-rapid fluid removal

Still, many experts limit rate of withdrawal by using gravity or hand collection techniques rather than a vacutainer, and remove no more than 1.5-2L of fluid at one time

A manometer may be used to ensure intrapleural pressure remains above -20cm H2O

Hemothorax

May be caused by vascular injury during insertion, intrathoracic mechanical injury from the needle or catheter, or pleural disruption in the setting of trapped lung physiology and excessively negative intrapleural pressures, especially with friable mucosa or coagulopathy

Use ultrasound to confirm size and location of pleural fluid, diaphragm, and lung

Color doppler at the insertion site can rule out large chest wall vessels in the insertion path

Consider trapped lung in chronic inflammatory effusions (e.g., malignant). Use a manometer or avoid large volume removal to avoid excessively negative pleural pressure

Pneumothorax

Pneumothorax is infrequent but can occur without any known tissue disruption during thoracentesis. The following may reduce the risk:

Use appropriate ultrasound to evaluate effusion size and location for catheter insertion

Consider trapped lung physiology in the appropriate clinical setting and manage as above

Ensure the patient is cooperative and has adequate analgesia to avoid inadvertent needle movement or catheter dislodgement

Evaluate for sliding lung at the end of the procedure to facilitate early detection

**Clinical Case**

***CC: Fever and shortness of breath***

**HPI:** A 72-year-old woman with a history of breast cancer in remission presents to the ED with fever, chest pain, and progressive shortness of breath over the last 3 weeks. She does not have any chronic respiratory diagnoses. She was recently told that her cancer may be recurring based on recent CT scans.

**All:** None

**Meds:** Trastuzumab, atenolol

**PMHx**: HTN, breast cancer thought to be in remission until recent imaging as above

**SocHx**: Married, retired architect. No significant travel history. No EtOH, tobacco, or other drug use

**FamHx:** Multiple family members with heart disease

**ROS:** complete ROS negative except as per HPI

***Pertinent PE:***

**Vitals:** Temp 37.8 HR 85, BP 105/65, RR 24, O2 90% on room air

**Gen:** Thin, looks fatigued and chronically ill. Minimally increased work of breathing, able to speak in short sentences

**CV:** Soft systolic murmur, not new

**Chest:** Decreased breath sounds 1/2 up the right posterior chest wall with dullness to percussion over the same area. Coarse rales and egophony audible in the right mid-lung.

**Abd:** Soft, nontender, normal bowel sounds

**Ext/Skin:** Warm with no edema or rash.

**Labs:**

Platelets are 40,000. Coagulation panel is normal

**Thoracic ultrasound:** Large effusion on the right. It is not overtly loculated, no fibrinous stranding, but there appears to be some increased echogenicity within the fluid. See image below.


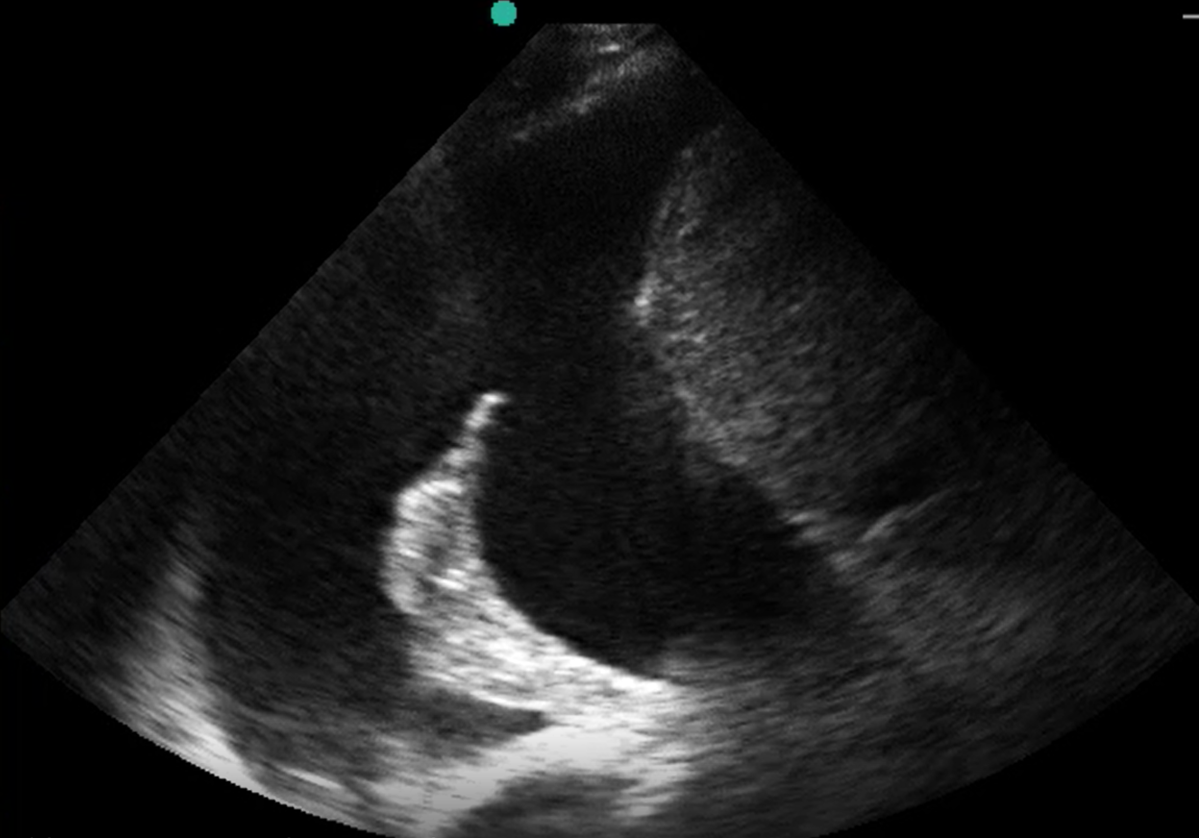


Fig 4. Ultrasound demonstrating pleural effusion. *Image author owned (AEM)*
