# Supplementary material for: A Multidisciplinary Curriculum to Standardize Chest Procedures Training for Trainees in General Surgery, Emergency Medicine, and Critical Care
Source: MedEdPORTAL. 2024 Jul 9;20:11421. doi: 10.15766/mep_2374-8265.11421 (PMC11231065; doi:10.15766/mep_2374-8265.11421)

## Slide 1
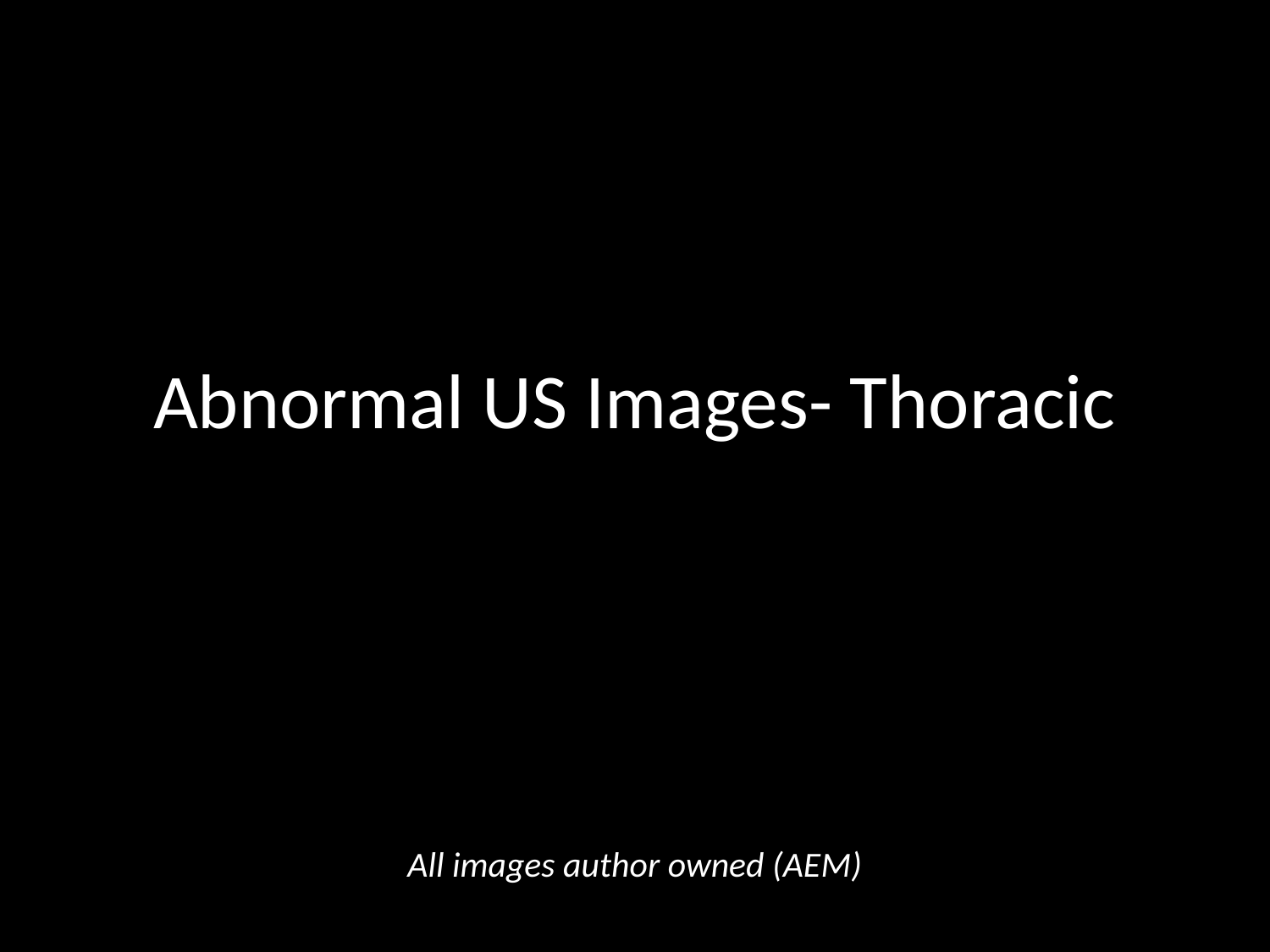

# Abnormal US Images- Thoracic
All images author owned (AEM)

## Slide 2
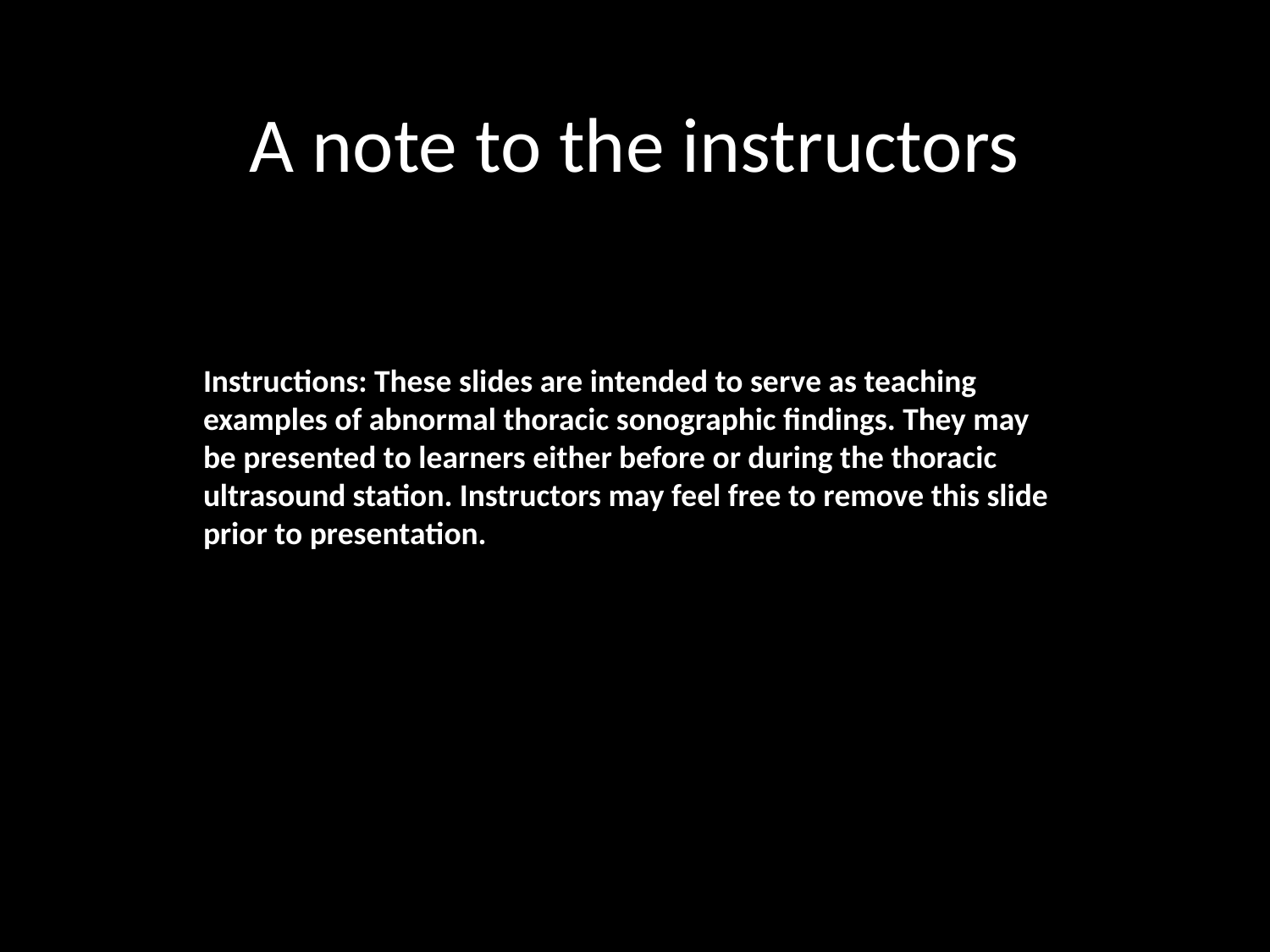

# A note to the instructors
Instructions: These slides are intended to serve as teaching examples of abnormal thoracic sonographic findings. They may be presented to learners either before or during the thoracic ultrasound station. Instructors may feel free to remove this slide prior to presentation.

## Slide 3
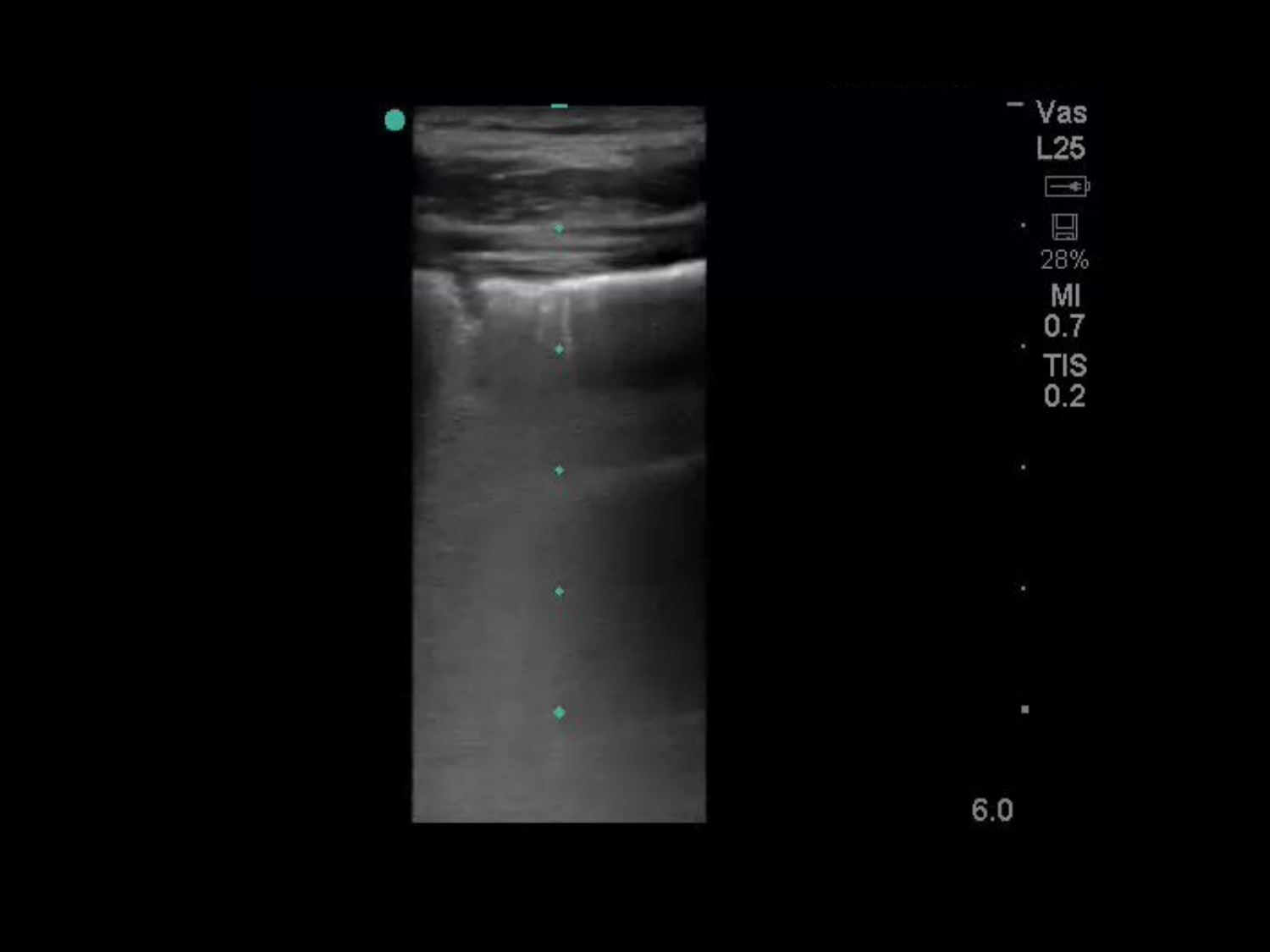

#

## Slide 4
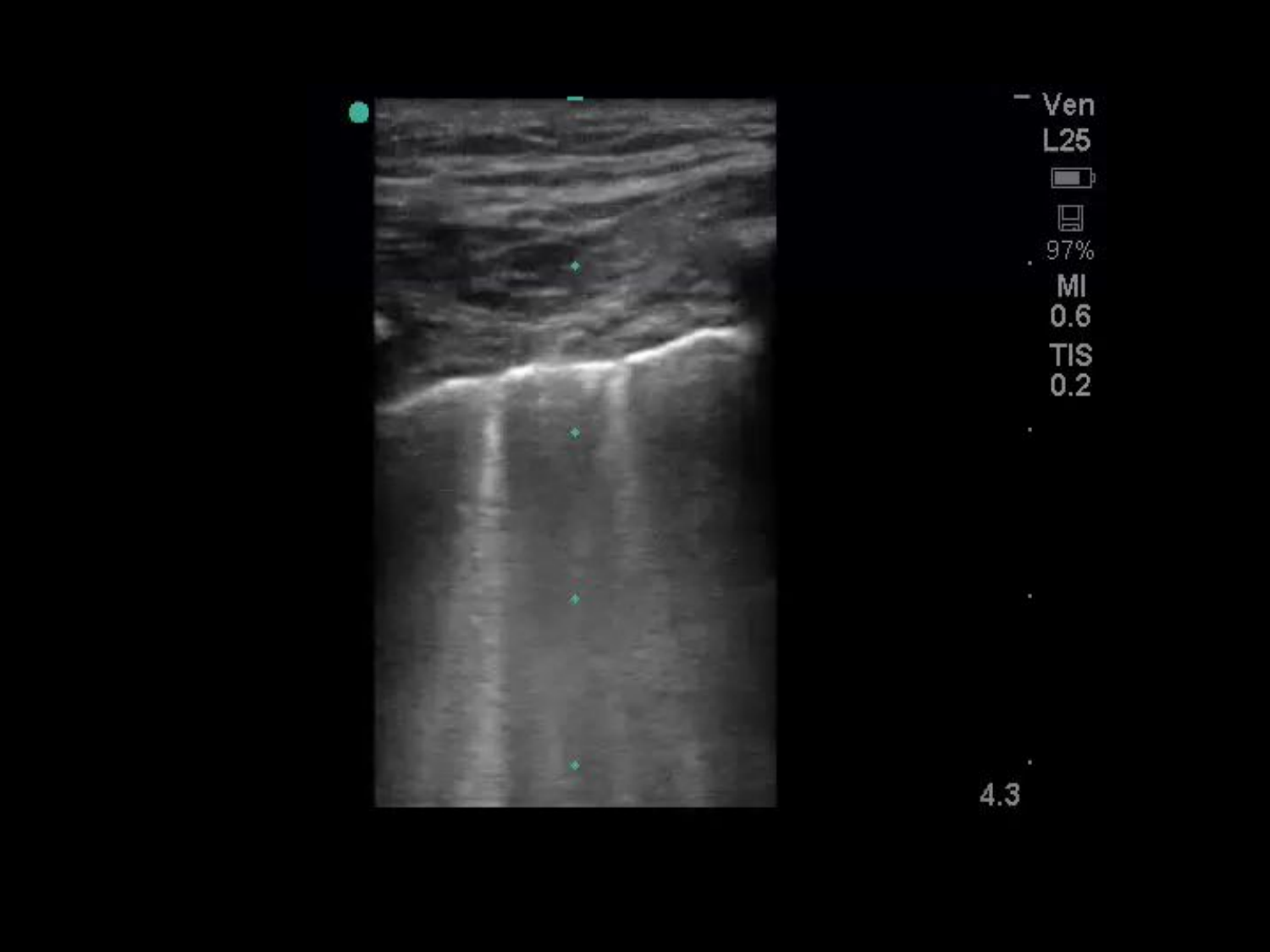

#

## Slide 5
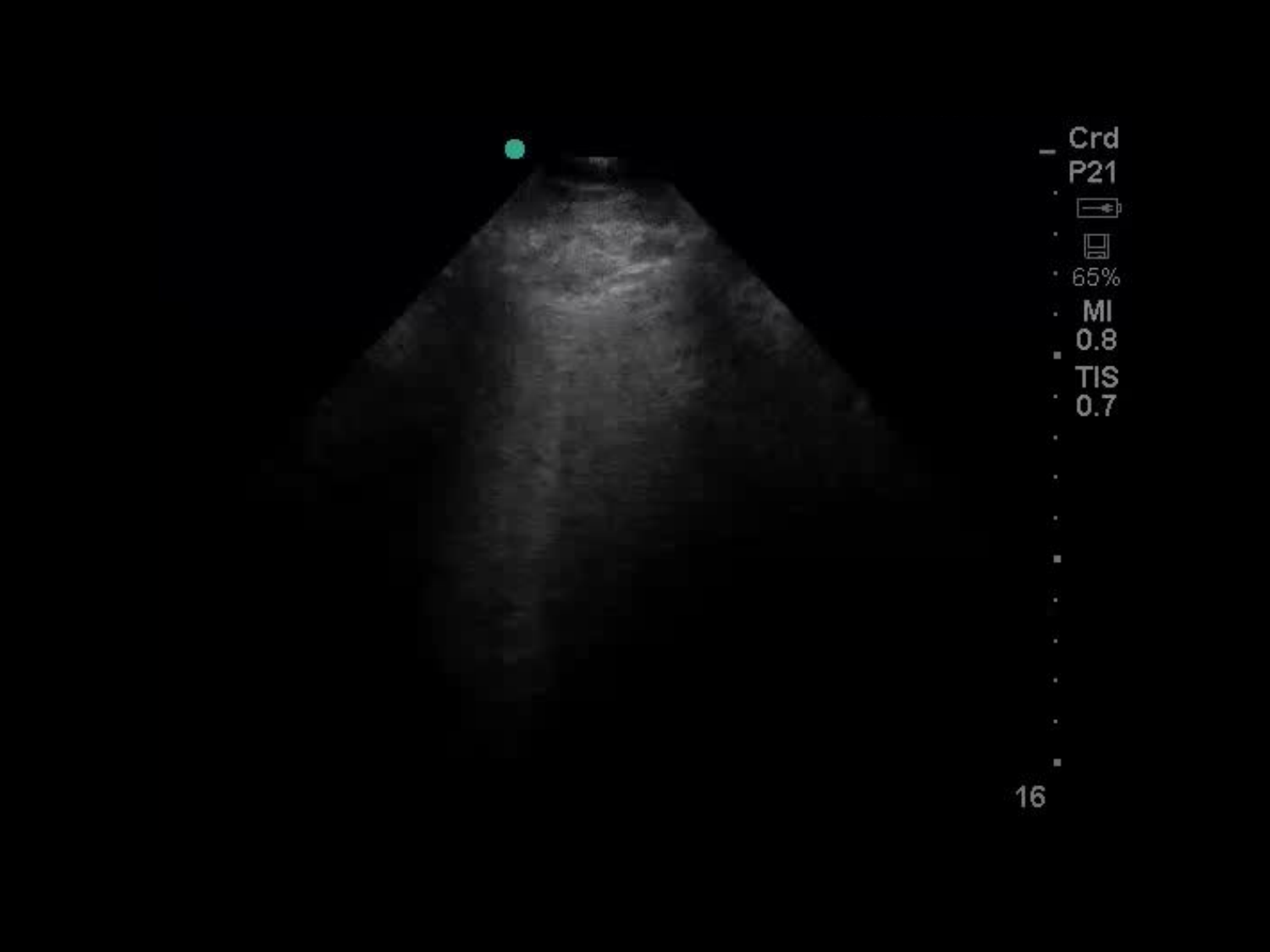

#

## Slide 6
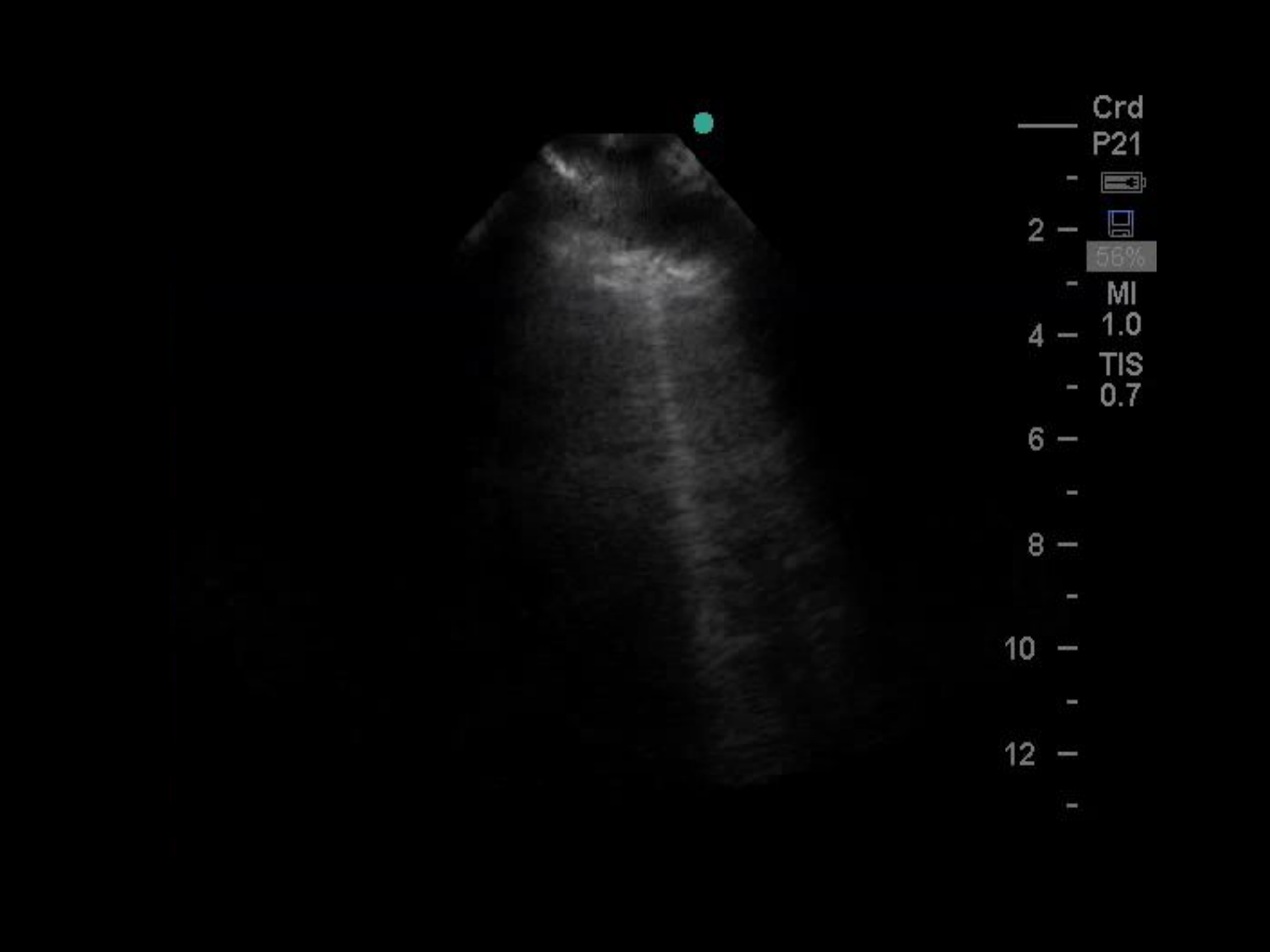

#

## Slide 7
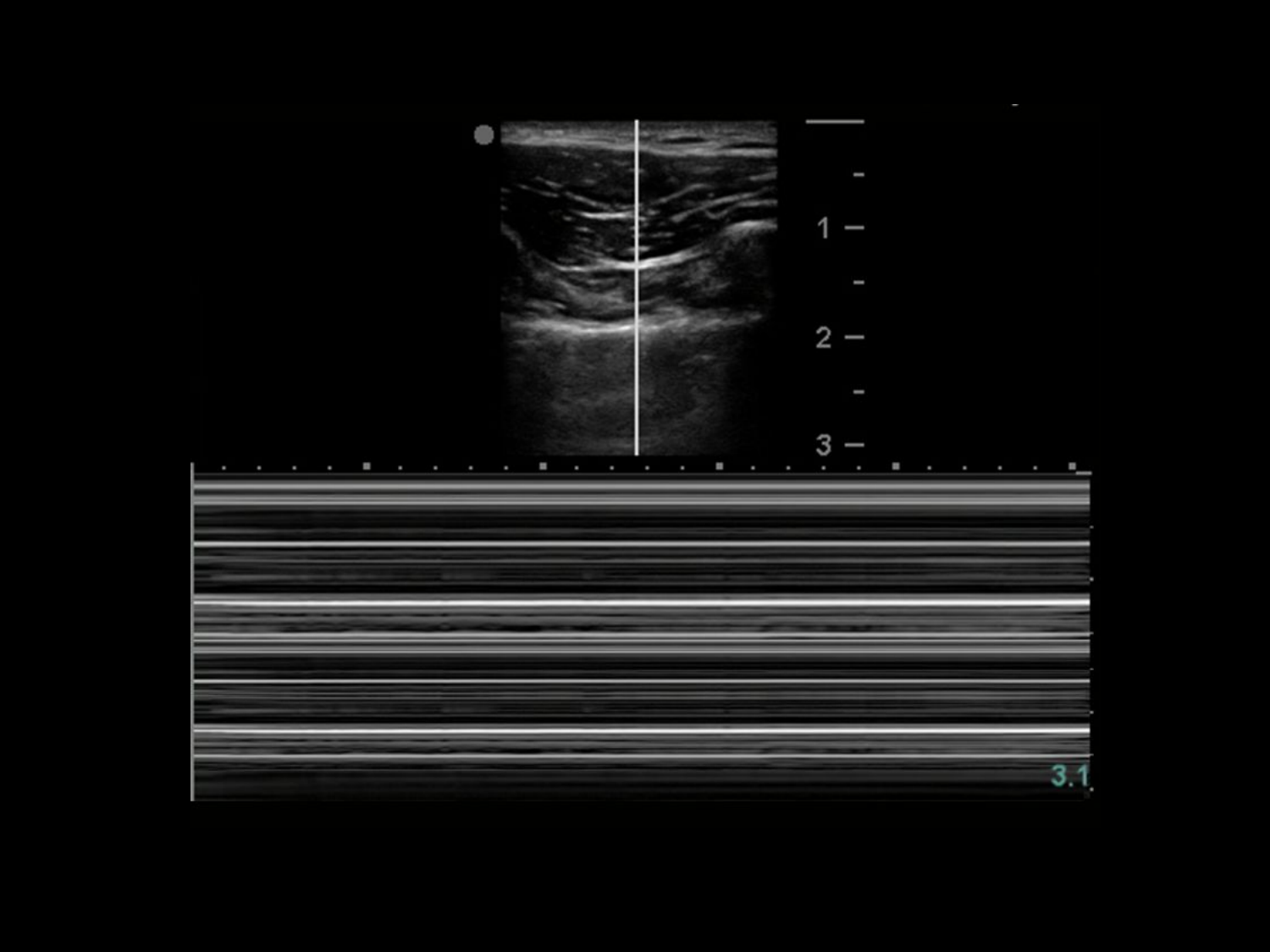

#

## Slide 8
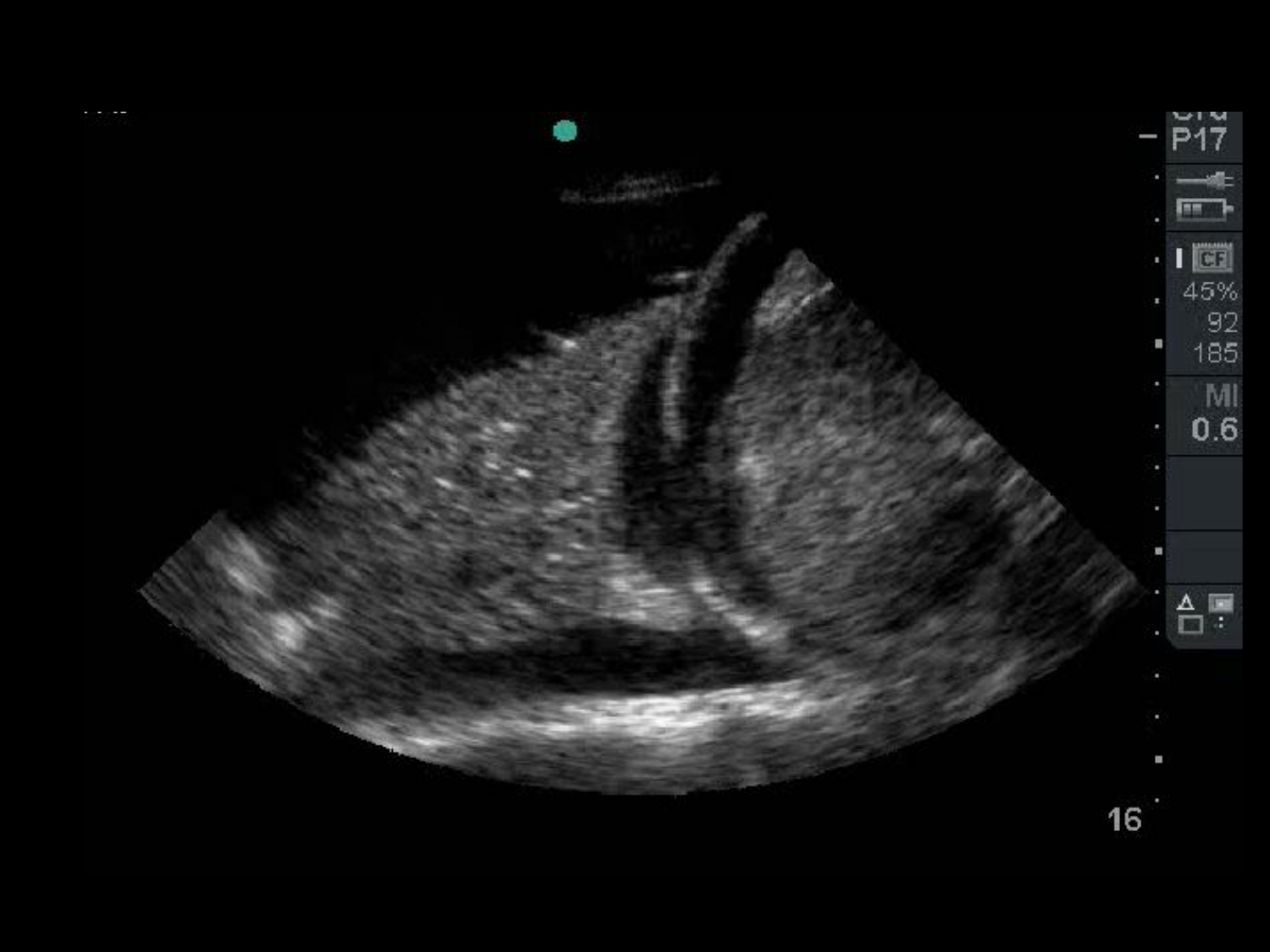

#

## Slide 9
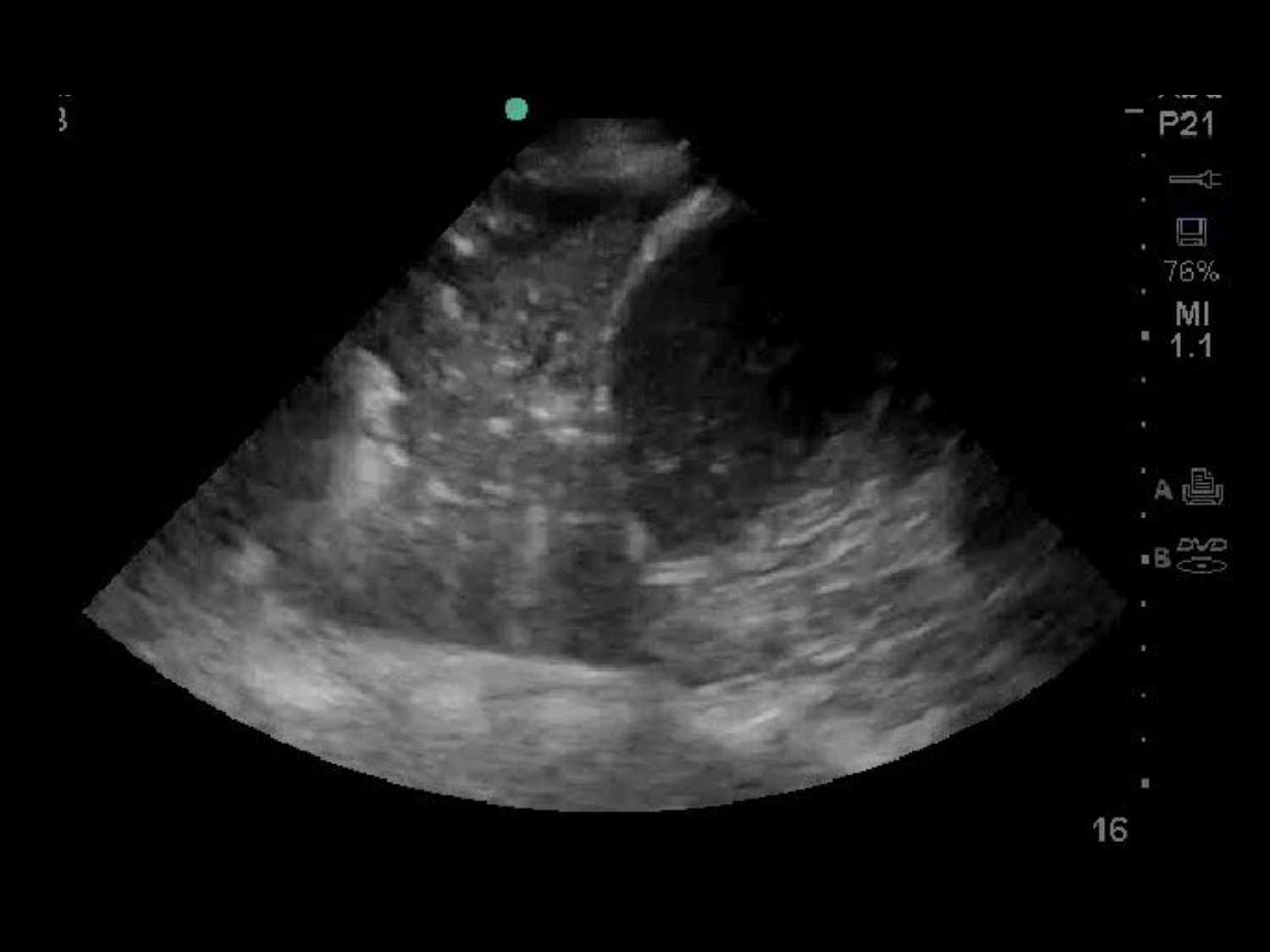

#

## Slide 10
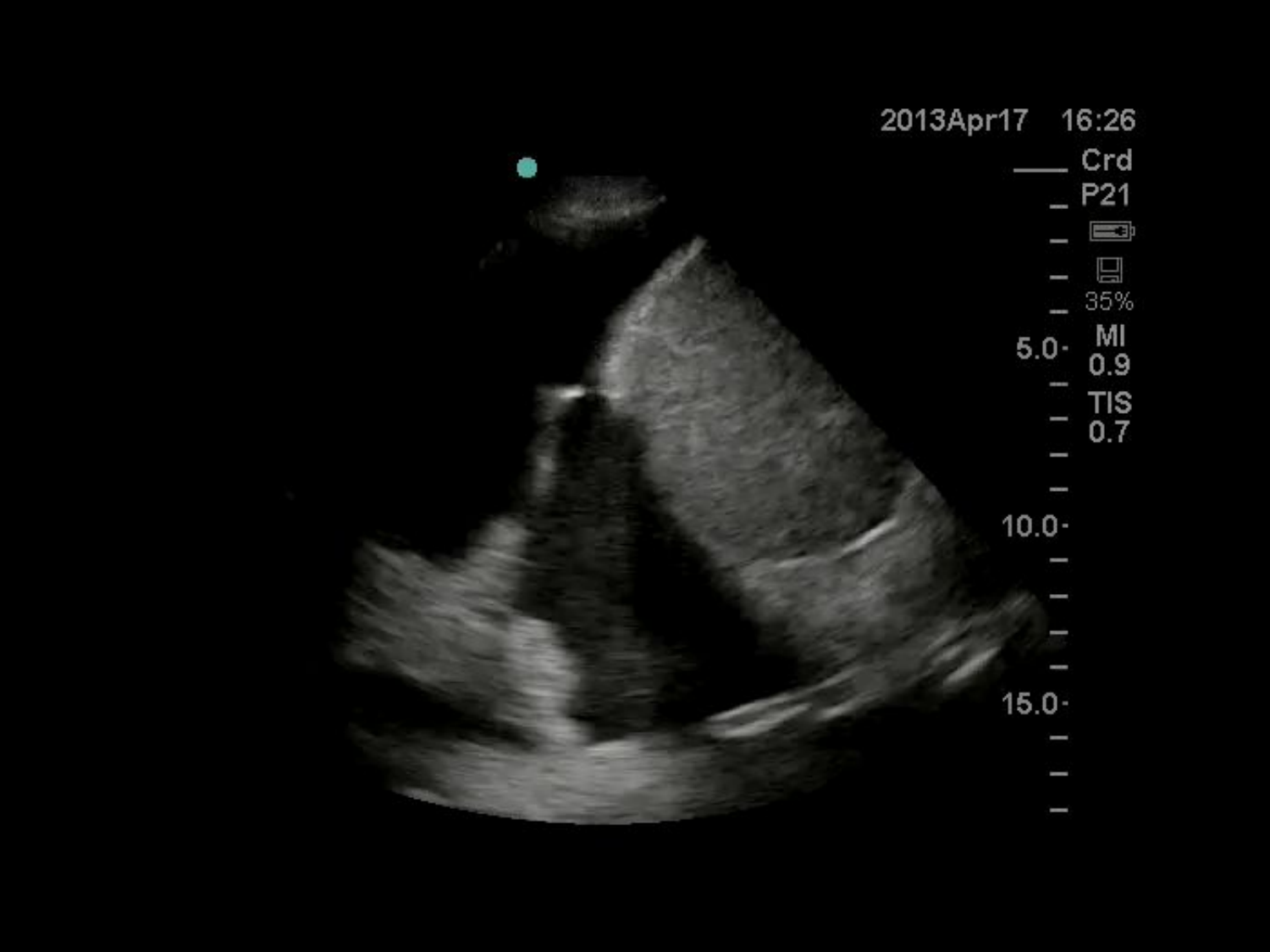

#

## Slide 11
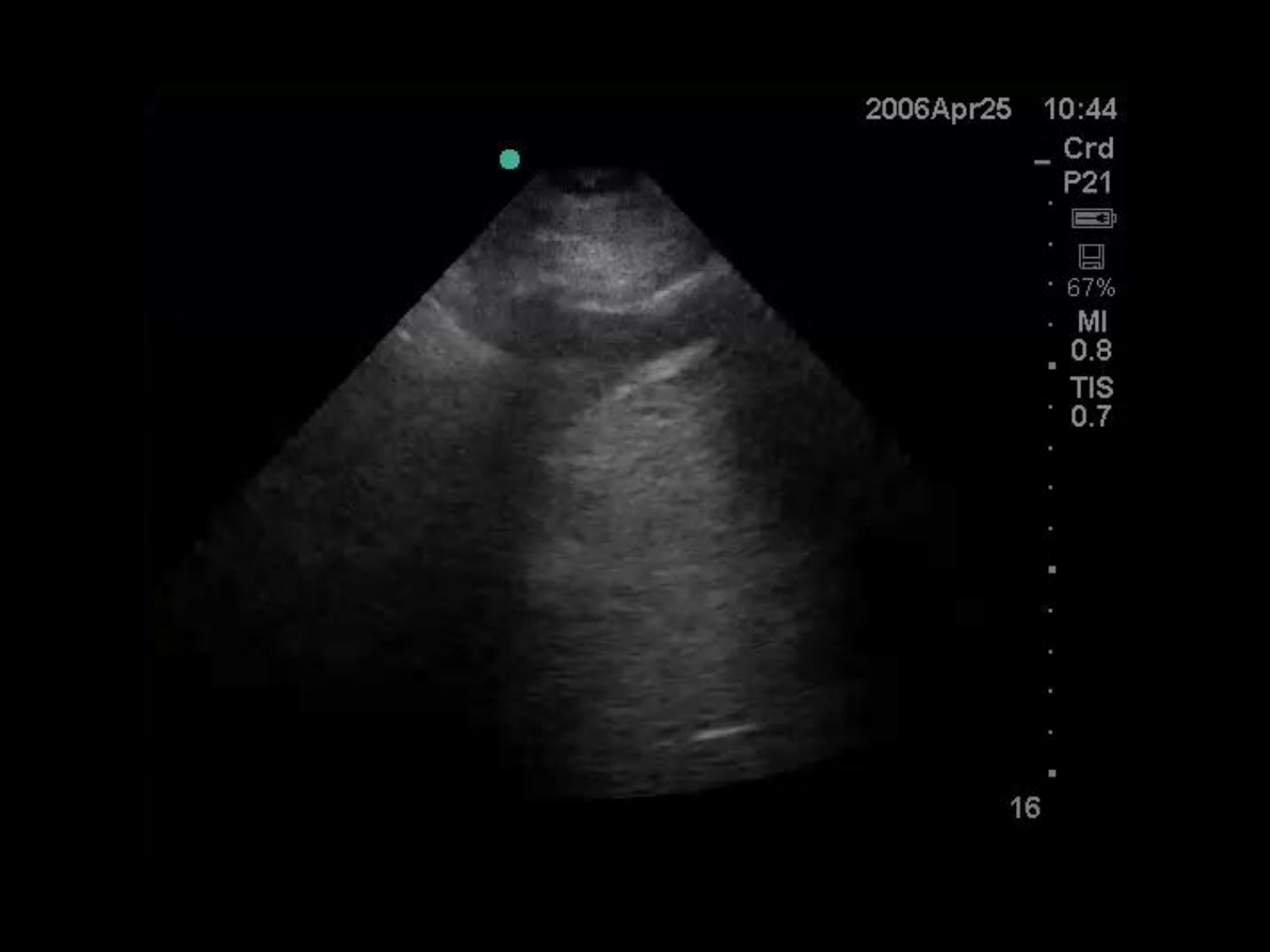

#

## Slide 12
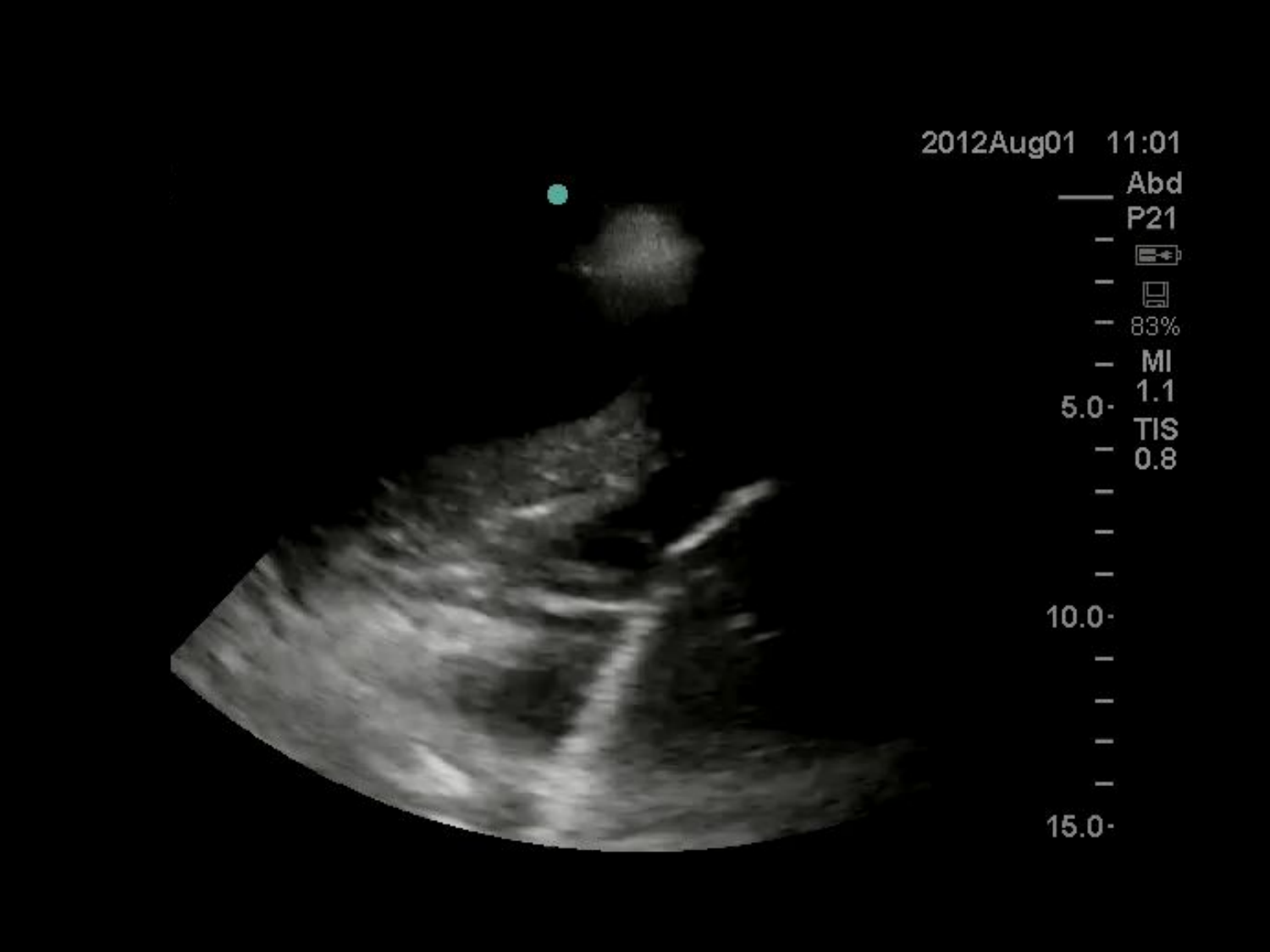

#

## Slide 13
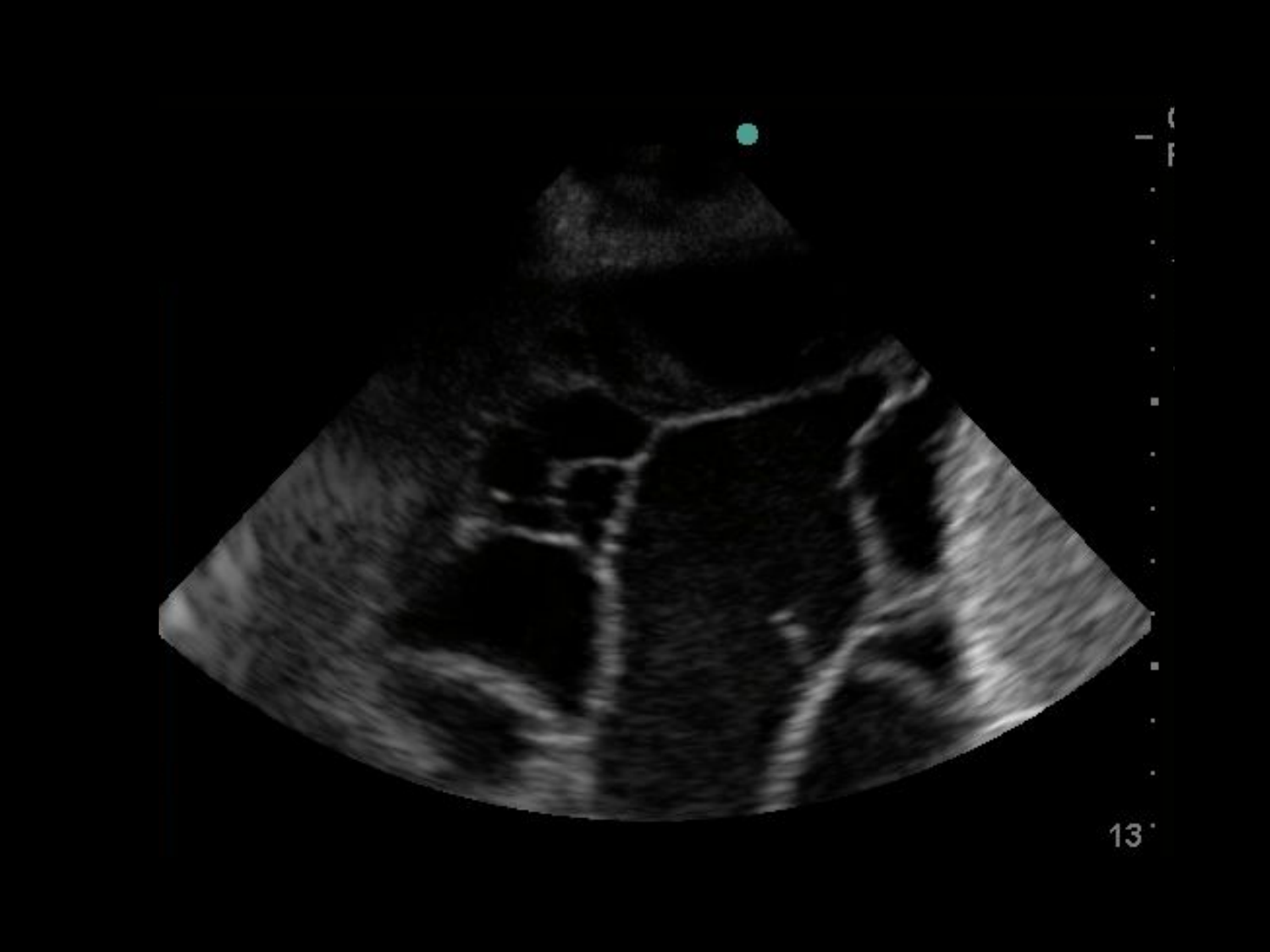

Supplement: Supplementary file 1 — Surgical Tube Thoracostomy Checklist.docxSample Workshop Schedule.docxInstructor Guide Surgical Chest Tube.docxInstructor Guide Seldinger Chest Tube.docxLow-Cost Chest Tube Model.docxInstructor Guide Chest Tube Securement Station.docxInstructor Guide Thoracentesis.docxInstructor Guide POCUS for Thoracic Procedures.docxThoracic Abnormal US Images.pptxChest Procedures Workshop Evaluation.docx [file mep_2374-8265.11421-s001.zip › I. Thoracic Abnormal US Images.pptx]
