# Supplementary material for: A Multidisciplinary Curriculum to Standardize Chest Procedures Training for Trainees in General Surgery, Emergency Medicine, and Critical Care
Source: MedEdPORTAL. 2024 Jul 9;20:11421. doi: 10.15766/mep_2374-8265.11421 (PMC11231065; doi:10.15766/mep_2374-8265.11421)
Supplement: Supplementary file 1 — Surgical Tube Thoracostomy Checklist.docxSample Workshop Schedule.docxInstructor Guide Surgical Chest Tube.docxInstructor Guide Seldinger Chest Tube.docxLow-Cost Chest Tube Model.docxInstructor Guide Chest Tube Securement Station.docxInstructor Guide Thoracentesis.docxInstructor Guide POCUS for Thoracic Procedures.docxThoracic Abnormal US Images.pptxChest Procedures Workshop Evaluation.docx [file mep_2374-8265.11421-s001.zip › D. Instructor Guide Seldinger Chest Tube.docx]

**Seldinger Technique Thoracostomy**

**Instructions: This instructor guide is to be used as a reference by faculty guiding the Seldinger chest tube placement station. It outlines the supplies needed, station setup, methods of instruction, steps of the procedure, common errors by trainees, assessment of trainees, and provides a clinical scenario through which to discuss the procedure in a clinical context.**

**
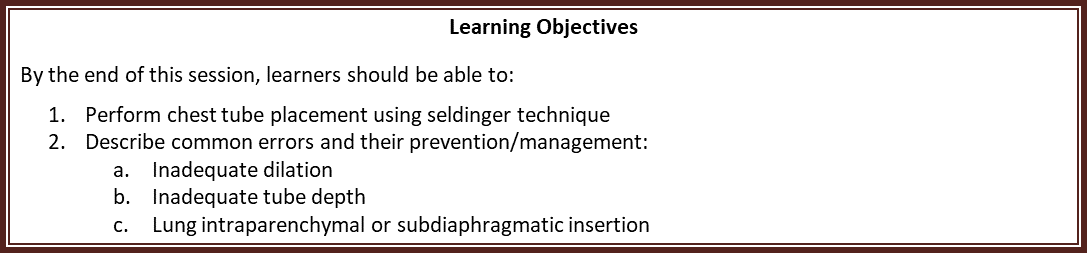
**

**Supplies**

- Table of similar height to patient bed, ideally with locking mechanism and or positioned against a wall for stability
- Gauze (1 box)
- Seldinger chest tube kit(s)
  - e.g., Wayne pneumothorax tray by Cook Medical (C-UTPTY-1400-WAYNE-112497-IMH)
  - Other institutions should use whatever kits/tubes are standard within their institution or departments
  - Minimum one kit for every two simultaneous learners, but kits can be reused for multiple groups throughout the workshop duration
- Thoracic Model: Three options in order of fidelity/complexity
- We use the low cost, high-fidelity model described and shown in Appendix E. Please see that appendix for details on what materials to purchase and how to construct the simulator. Alternatively, either of the following low-cost, low fidelity models can be used:

1. “Banana” model (Fig. 2)
   - Tupperware container (5” x 7” x 3” works well) with hole cut in lid ~2” x 1”
   - Banana with 2” section of peel removed on one side
   - Wide tape (any kind)
2. Simple rib model (Fig. 3)
   - (4) ½” pipe 48” length
   - (2) H-style ½” pipe clamp fixture
   - (4) S-hooks and/or zip ties (several)
   - (4) Perpendicular IV pole clamp
   - Large fluid basin
   - Silk tape
   - 1L bag IV fluids
   - Plastic food wrap
   - Rack of pork ribs (sheep ribs are alternative)

Consider starting the session with the clinical case provided at the end of this document.

**Station Setup**

- Optimize number of models and kits for learner groups: one setup per learner provides maximum hands-on time, but two learners per model works well with the time allotted here.
- Adequate table space should be available for learners to lay out equipment from Seldinger tube thoracostomy kits. Kits are ideally provided in original packaging so learners can familiarize themselves with all components, but the essential elements are pictured below.


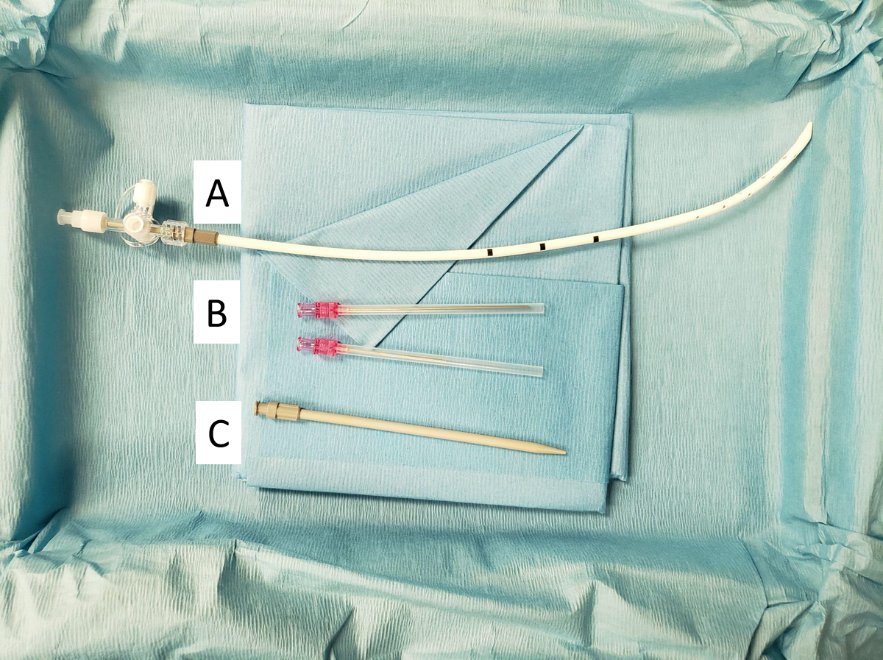


Figure 1. Essential components of the Seldinger technique tube thoracostomy kit. A. Pigtail catheter with trocar in place, straightening the distal pigtail curl. B. Introducer needles of two different sizes. C. Dilator. Not pictured: flexible wire. *Image author owned (RJK)*

- Banana model
  - Fill the Tupperware container ~2/3 with water. A few drops of food coloring can assist learners in visualizing aspiration of fluid
  - Place the banana, peel removed side down, over the hole in the lid
  - Secure with tape


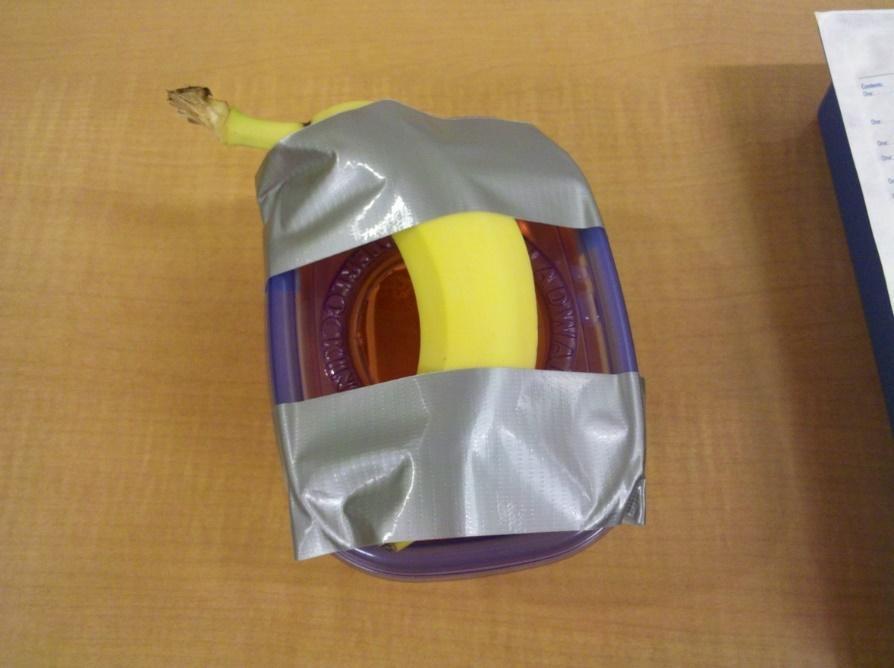

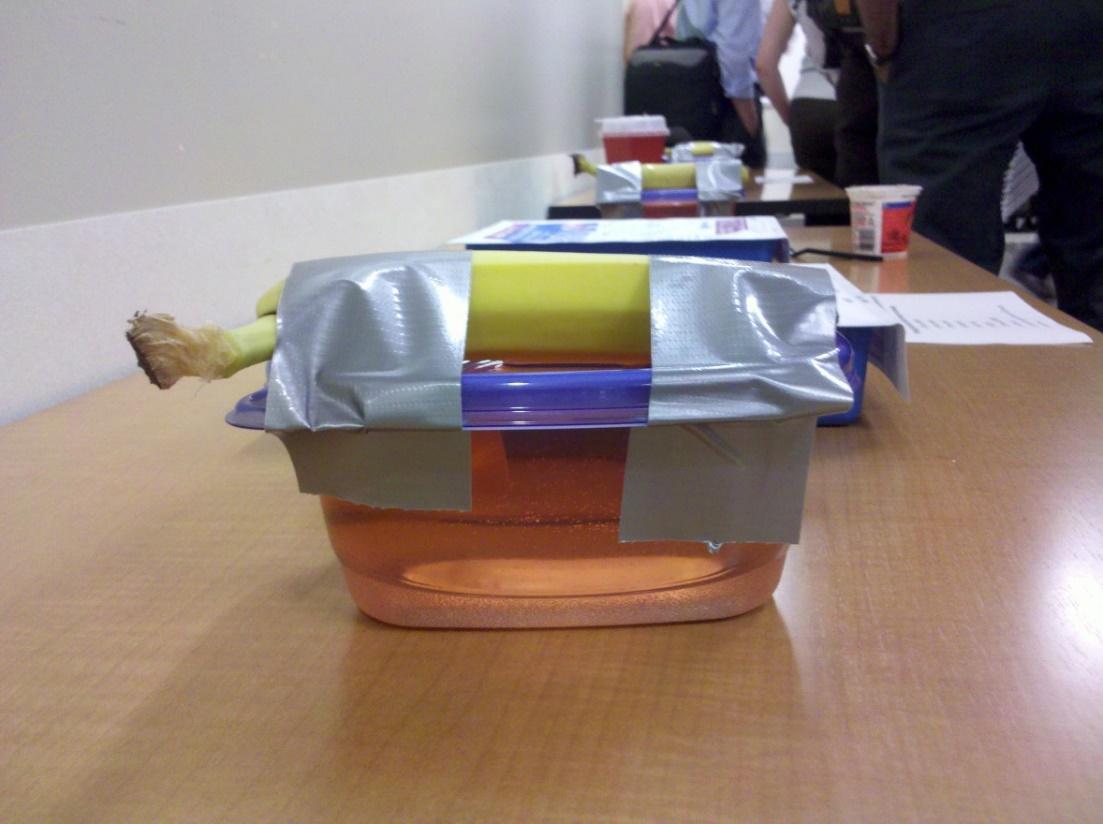


Figure 2a. (left) Banana model from above. The peel simulates patient skin and is removed on the posterior aspect of the banana overlying a small hole cut into the container lid (not seen)

Figure 2b. (right) Banana model from the side. The fluid in the container simulates pleural fluid for aspiration.

*Images author owned (AEM)*

- Simple thoracic rib model
  - See Appendix E for instructions on constructing the pipe frame
  - If desired, affix 1L bag of IV fluid to the pleural surface of the ribs
  - Wrapping the ribs and fluid bag in plastic food wrap helps to minimize fluid loss from the IV bag when punctured
  - Model can be hung by hooks or zip ties with ribs in a horizontal or vertical orientation; vertical rib orientation better simulates supine patient positioning


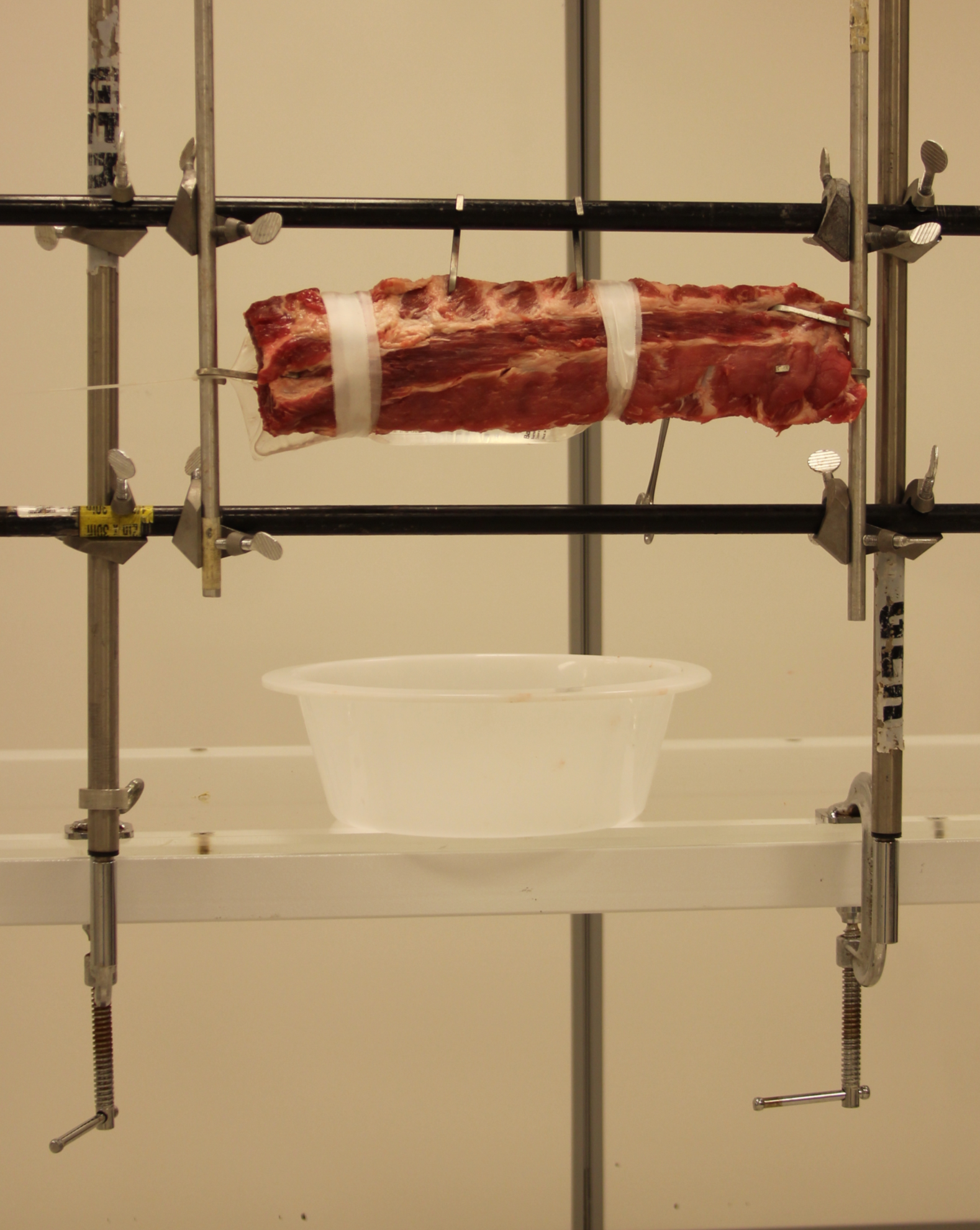

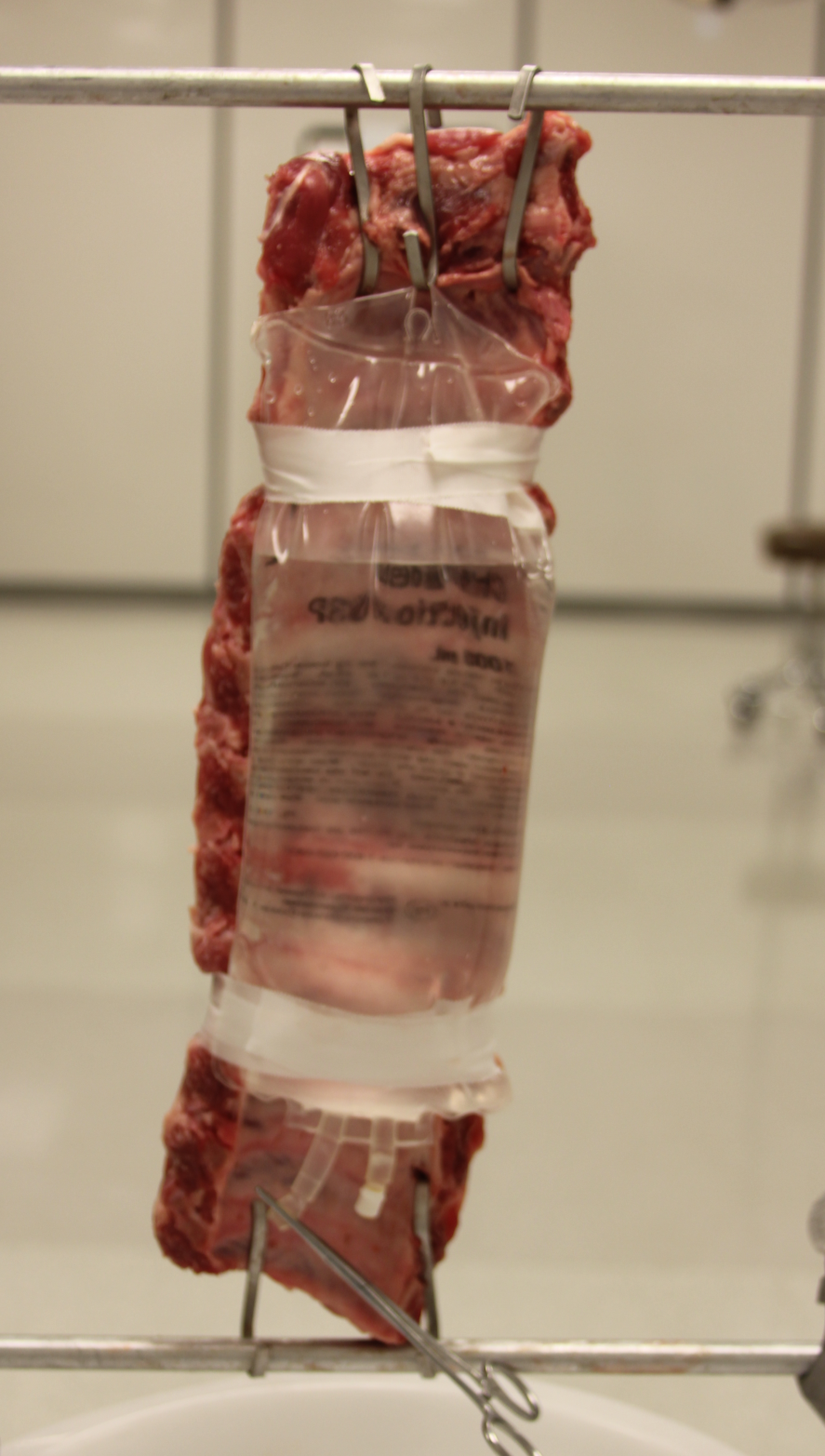


Figure 3a. (left) The rib model is seen with ribs oriented vertically as in a supine patient. The learners perform the procedures on the muscular side of the model. Note the large basin underlying the model to catch fluid drips. Wrapping the entire model with plastic food wrap (not seen in this image) can better approximate ribs and IV fluid bag, reducing fluid loss.

Figure 3b. (right) The rib model is seen with ribs oriented horizontally, as in a patient who is seated upright. This orientation can also be used for thoracentesis or pleural biopsy training. The “pleural” side of the model is seen here, with the bag of IV fluid as a model of pleural effusion.

*Images author owned (BS)*

**Prerequisites**

*Required background knowledge*

- Understanding of anatomy and physiology of the chest wall, pleura, and lungs
- Clinical indications for Seldinger tube thoracostomy, as differentiated from indications for thoracentesis or placement of a surgical chest tube

*Required background skills expected in trainees prior to receiving training in the target course:*

- Learners should have the following essential skills prior to this workshop:
  - Creation of a sterile field
  - Administration of local anesthetic
  - Performance of Seldinger technique in other applications (e.g., central venous catheter)

**Step 1: Expert Description (5 minutes)**

1. **Preparation**

Prior to demonstrating placement, instructor should discuss:

- 1. Optimal patient positioning
  2. Key anatomic structures
  3. The importance of patient comfort using topical analgesia
  4. Optimal insertion locations in the following situations:
     1. Pneumothorax
     2. Pleural effusion*

* Ultrasound is used to guide location of Seldinger tubes for pleural effusions. This will be discussed in detail at the Ultrasound station of this workshop

1. **Equipment**

Prior to demonstrating placement, instructor should review key equipment including:

- 1. Contents of the chest tube kit
  2. Any additional items not included in the kit that learners should gather beforehand. For example, some kits do not contain local anesthetic. Additional equipment can be gathered a la carte, or by using an additional kit that contains the needed items (e.g., a CVC kit)

The drainage collection system will be discussed at the Tube Thoracostomy Securement and Troubleshooting station, and should not be discussed here.

**Step 2: Expert Demonstration (5 minutes)**

Instructor should perform Seldinger tube thoracostomy and narrate key technique elements per the procedural steps below. Alternatively, instructors may prefer to verbally instruct a learner as they perform the steps of the procedure as other learners observe.

**Step 3: Learner Hands-on Practice (35 minutes)**

- Learners can practice assembling the catheter equipment while awaiting their turn on the simulator
- If more than 2 learners are present, additional practice opportunities with a low fidelity model is recommended
- If more than 4 learners are present, a second high fidelity manikin and ideally a second instructor should be used to ensure adequate hands-on time to reach adequate performance during a 45-minute rotation
- The instructor should avoid repeating their expert demonstration if possible, using verbal instruction to guide learners through difficult steps in order to maximize hands-on time

**Step 4: Assessment**

- In most workshops, competent performance is determined by the instructor using informal global assessment during the course of the station
- Successful performance must include all elements of the steps outlined below:
  - Anesthesia (verbalized, pantomimed)
  - Procedure
  - Equipment cleanup, including removal of sharps from the field
- If learners are unable to perform all steps of the procedure to the satisfaction of the instructor by the end of the station rotation, the instructor must discuss with the workshop director the need for additional training at a later date

**Seldinger Tube Thoracostomy Steps**

**Notes:**

* Section I. “Preparation” and much of section II. “Positioning and draping” are identical to the steps in the Surgical Tube Thoracostomy station. Instructors may choose not to repeat those steps here, particularly if learners have already completed the Surgical Tube Thoracostomy station.

** Section VIII. “Securing and Cleaning up” is included in procedural steps for completeness, but this section is covered at the Tube Thoracostomy Securement and Troubleshooting station.

1. Preparation:
2. Discuss indications of the procedure with the attending. Address duration of placement and plans for removal.
3. Notify nursing staff of the procedure.
4. Ensure that all equipment is present and easily accessible from patient’s bedside.
5. Obtain informed consent.
6. Discussion of relative contraindications to urgent/non-emergent chest tube placement including:
   1. INR >1.5 (<https://www.ncbi.nlm.nih.gov/pmc/articles/PMC6026252/>)
   2. PLT <50 (<https://www.ncbi.nlm.nih.gov/pmc/articles/PMC6142536/>)
7. Patient should be placed on nasal oxygen and continuous pulse oximetry.
8. Verify patient identity and procedure location during “Time Out.”
9. Positioning and draping
   1. Position the patient: for anterior axillary line insertion, head of the bed should be elevated 30-60 degrees, and the arm on the procedural side is restrained over the patient’s head.
   2. Locate and describe the appropriate landmarks outlining the triangle of safety and indicate the site of tube placement verbally.
   3. Prep a wide sterile field around the site of insertion.
10. Equipment preparation
    1. Lay out kit contents in the order of use to maximize procedural efficiency.
    2. Load trocar to stiffen the flexible catheter.
    3. Lubricate the dilator and catheter with sterile saline.
11. Analgesia/Sedation
    1. Options include:
       1. Moderate sedation
       2. Topical analgesia using generous amounts of local anesthetic
       3. Regional nerve blocks
       4. Procedural sedation and analgesia are beyond the scope of this curriculum, but learners should be aware of these options
12. Insert wire
    1. Choose the appropriate introducer needle length based on pleural depth by ultrasound and confirmed by anesthesia step above.
    2. Load 2-3 cc saline (or local anesthetic such as lidocaine) in a syringe and aspirate as you advance.
    3. When fluid (for effusion) or bubbles (for pneumothorax) appear in the syringe, advance ~5mm and ensure pleural fluid aspirates easily; for pneumothorax, inject ~1cc saline/local anesthetic to ensure smooth flow.
    4. Stabilize hand position against chest wall to prevent needle advance/withdrawal and remove syringe.
    5. Feed the wire through the needle until there is ~10cm remaining.
    6. Remove the needle.
13. Dilate
    1. Nick the skin at the needle site by inserting the scalpel alongside the wire to the scalpel’s hub, making a 1cm incision.
    2. Confirm a skin bridge is not present by moving the wire within the incision and ensuring it moves freely.
    3. Insert one dilator to the necessary depth to penetrate pleura based on subcutaneous tissue thickness estimated by ultrasound and confirmed by steps above.
    4. Slide the dilator in and out 2-3 times to ensure it moves freely, then remove.
14. Insert catheter
    1. Feed catheter (with trocar in place so it is stiff) over wire.
    2. Advance catheter/trocar and wire as a unit at least as far as estimated pleural depth plus 1-2cm (see dilator insertion depth above).
    3. At this point the trocar and wire may be held steady while the catheter is advanced to at least the first black line (5 cm proximal to the last sidehole) and deep enough to ensure the last sidehole is intrapleural.
    4. Remove the trocar and wire together.
15. Secure the tube and clean up
    1. Secure the chest tube at the insertion site with 0 or 1-0 silk by placing a U-stitch around the incision site and wrapping the suture several times around the chest tube before knotting several times. Tube should be fixed sufficiently tightly to prevent accidental removal but not so tight as to impair tube drainage.
    2. Cover the insertion site with foam tape.
    3. Connect chest tube to closed drainage system (or flutter valve). Secure connection points with foam tape.
    4. Clean off patient and secure redundant tubing.
    5. Dispose of sharps properly.
    6. Document procedure protocol in patient’s chart.
    7. Communication with patient and family, attending, nursing staff and provide appropriate sign out to primary medical team.

**Common Errors and Their Remedy**

1. Inadequate dilation
   1. Resistance immediately upon inserting the dilator through the skin suggests inadequate incision length or depth. Be sure the incision is large enough to allow passage of the dilator through the skin.
   2. Resistance deep to the skin is normal as the dilator travels through the intercostal tissue and pleura.
2. Inadequate tube depth
   1. Ensure the tube is inserted far enough that all islet holes have passed through the pleura into the pleural space.
   2. Pleural depth can be estimated by ultrasound and confirmed with depth to fluid or air aspiration.
   3. The catheter must traverse the pleura with the trocar in place. Past this point, the catheter may be advanced further into the pleural space with the trocar held still (so the catheter is advancing, but not the trocar). If the trocar is not advanced through the pleura, the catheter will curl in the subcutaneous tissues as it is too soft to traverse through the pleura without the rigidity of the trocar.
   4. For this reason, in the setting of a large pneumothorax or effusion, err on the side of deeper insertion with the trocar in place to stiffen the pigtail catheter.
3. Lung Intraparenchymal or subdiaphragmatic insertion
   1. Chest tube kits that do not employ the Seldinger wire technique typically load the catheter directly onto a sharp, rigid needle or trocar that can puncture directly into the lung or through the diaphragm if inserted too deeply. The Seldinger technique above is safer.
   2. However, even with the Seldinger technique if the needle is inserted too deep or an incorrect site is chosen, the needle and therefore wire could enter the lung, leading to intraparenchymal catheter insertion.
      1. Use ultrasound to confirm correct insertion site, ideally within the triangle of safety. The diaphragm is often more superior than anticipated, particularly in supine and critically ill patients.
      2. Use the shortest needle needed to enter the pleura, as estimated by ultrasound and depth to the pleural space as determined by fluid or air aspiration during anesthesia.
      3. If resistance is encountered with wire advance, stop and reassess needle location and depth.
   3. Intrapleural adhesions in which visceral pleura is adherent to parietal pleura will increase the risk of parenchymal injury. Imaging findings that suggest this phenomenon should prompt consideration of surgical tube thoracostomy or placement of a Seldinger technique catheter under real-time (as opposed to static, or “marking” ultrasound guidance.)

**Clinical Case**

For simplicity, we recommend using the same case as provided in Appendix C. Surgical Tube Thoracostomy Instructor Guide, but without evidence of lung adherent to chest wall on ultrasound. The absence of that finding increases the safety of Seldinger technique tube thoracostomy as opposed to surgical technique, which allows for manual sweep of the intrapleural space to ensure the tube does not enter lung parenchyma. Real time ultrasound guidance as discussed above would also increase the safety of Seldinger technique thoracostomy in that situation.

***CC: Fever and shortness of breath***

**HPI:** A 67-year-old man presents to the ED with fever, cough productive of purulent sputum and progressive shortness of breath over the last 3 days. He denies hemoptysis but does endorse pleuritic chest discomfort on the right side. He does not have any chronic respiratory diagnoses but has smoked 1 pack of cigarettes per day for 40 years.

**All:** None

**Meds:** lisinopril, aspirin, metformin

**PMHx**: HTN, type II diabetes mellitus

**SocHx**: Married, retired construction supervisor. Born and raised near Seattle, no significant travel history. Moderate EtOH use, tob as above, no other drug use.

**FamHx:** Father died of MI in his 80s, mother had HTN and diabetes, died of a stroke in her 70s

**ROS:** complete ROS negative except as per HPI

***Pertinent PE:***

**Vitals:** Temp 38.9 HR 114, BP 90/60, RR 24, O2 90% on room air

**Gen:** Mild obesity, looks acutely ill. Increased work of breathing but is able to speak in short sentences

**CV:** Tachycardic, no murmur

**Chest:** Decreased breath sounds 1/2 up the right posterior chest wall with dullness to percussion over the same area. Coarse rales and egophony audible in the right mid-lung.

**Abd:** Soft, nontender, normal bowel sounds

**Ext/Skin:** Warm with no edema or rash.

**Thoracic ultrasound:** Large complex pleural effusion on the right. In contrast to the case at the surgical tube thoracostomy station, there is no suggestion that lung may be adherent to the chest wall.


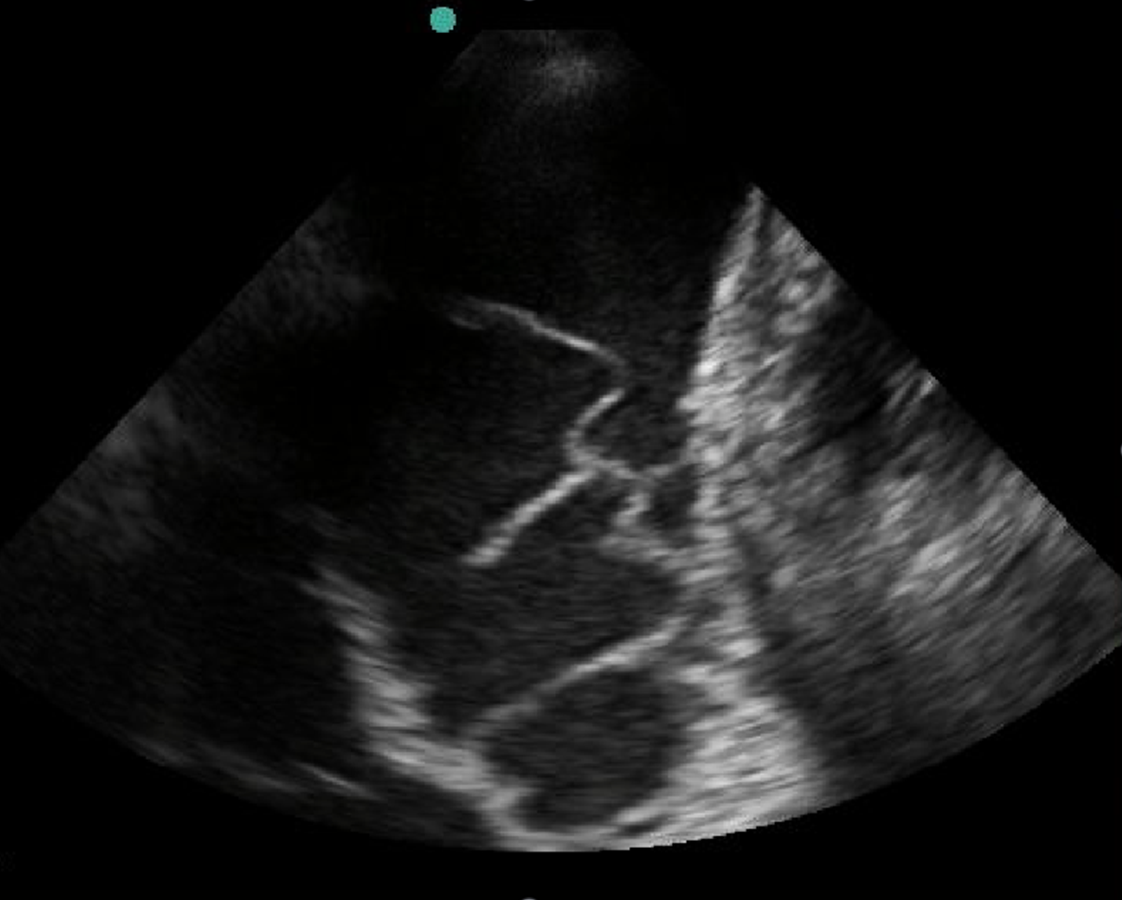


Figure 4. Still image from lung ultrasound demonstrating complex pleural effusion without appearance of lung adherent to chest wall. *Image author owned (AEM)*
