# Supplementary material for: A Multidisciplinary Curriculum to Standardize Chest Procedures Training for Trainees in General Surgery, Emergency Medicine, and Critical Care
Source: MedEdPORTAL. 2024 Jul 9;20:11421. doi: 10.15766/mep_2374-8265.11421 (PMC11231065; doi:10.15766/mep_2374-8265.11421)
Supplement: Supplementary file 1 — Surgical Tube Thoracostomy Checklist.docxSample Workshop Schedule.docxInstructor Guide Surgical Chest Tube.docxInstructor Guide Seldinger Chest Tube.docxLow-Cost Chest Tube Model.docxInstructor Guide Chest Tube Securement Station.docxInstructor Guide Thoracentesis.docxInstructor Guide POCUS for Thoracic Procedures.docxThoracic Abnormal US Images.pptxChest Procedures Workshop Evaluation.docx [file mep_2374-8265.11421-s001.zip › E. Low-Cost Chest Tube Model.docx]

**A Low Cost and High Fidelity Alternative to**

**Commercially Available Surgical Tube Thoracostomy Practice Manikins**

**Instructions: This guide is meant to serve as a method for building a cost effective, high fidelity alternative to expensive commercially available tube thoracostomy training manikins. It outlines supplies needed with estimated costs and method of construction of the model. This is the high-fidelity, low cost simulator referred to in the ESR and Appendix D while discussing the “Seldinger Tube Thoracostomy” station.**

Developed by Beth Sobba MS1, Dr. Hugh Foy, Dr. Genevieve Pagalilauan, Dr. Keiran Warner, Dr. Amy Morris, and Leann Elder at the WWAMI Institute for Simulation in Healthcare (WISH), a part of the University of Washington School of Medicine, Seattle Washington.

**Summary of Instructional Guide**

1. Objective
2. Equipment List
3. Setup Images
4. Additional Resources
   1. Recipe for making silicone “skin” for procedure models
   2. Cost estimates for reusable and non-reusable items
5. **Objective**

This model is a high fidelity, low cost alternative simulation model for practicing surgical tube thoracostomy. The model replicates a human thoracic wall using animal (pork or sheep) ribs, neoprene, foam, and special effects quality simulation skin. The tissue layers sit flush upon one another, and the taut neoprene creates a realistic “pop” sensation similar to the pleura. The frame that holds the model provides stability and is adjustable to various sizes of ribs and tissue tensions. The initial startup materials are available via local hardware stores and online retailers with an estimated total cost of approximately $380. These items only need to be purchased once, and are sturdy enough to be reused many times over. Per each session of 8-10 learners (depending on the number of intercostal spaces on ribs), non-reusable materials have an estimated total cost of approximately $30. Compare this to a commonly used commercially available product: the TraumaMan™ system costs approximately $25,000. Tissues can be used approximately 8-10 times before needing replacement, and tissue replacements cost $200 each.

1. **Equipment List**

Frame supplies:

- - (6) Black pipes ½” diameter 48” length
  - (2) H-style pipe clamp fixture for ½” diameter black pipe
  - (8) Perpendicular IV pole clamp^1^
  - Table *preferably with wheels that lock and height similar to patient bed

Chest model supplies:

- Animal ribs (pork used in our institution, but sheep is a good alternative)
- Neoprene 2mm thick (diameter larger than diameter of black pipe)^2^
- Foam ¼” thickness, cut somewhat larger than the size of ribs. *Preferably a different color than the neoprene.
- Animal (pig) skin or Smooth-On Skin (see additional resources for Smooth-On recipe)
- (20+) Zip ties 8-11” length

Additional supplies:

- Chux pads – to protect floor from pork rib drippings
- Surgical towels - to create “sterile field” (optional) and for clean up
- Drape for table
- Marking pen or permanent marker

Surgical instruments:

- Chest tube
- Scalpel
- (2) Kelly clamps
- (2) Curved hemostats
- (4) Towel clamps – if creating a “sterile field” with surgical towels.

^1^ e.g. Fisherbrand™ Castaloy™ Clamp Jumbo Holder can be purchased directly from [Fisher Scientific](https://www.fishersci.com/shop/products/thermo-scientific-castaloy-clamp-jumbo-holder/05757q.). These clamps are also commonly found in hospital operating rooms, used by anesthesia to secure the cross bar that sections off anesthesia from the surgical sterile field.

^2^ The ends of the neoprene are sewn in advance to form “sleeves” that slide over the ½” black pipe of the frame (see photos). Hand sewing has been effective in securing the sleeves. A system of zip ties could also work. Glue and stapling proved to be too weak to withstand the tension applied to the neoprene when it is stretched during setup.

1. **Setup Images**

*All images in this section courtesy of author BS
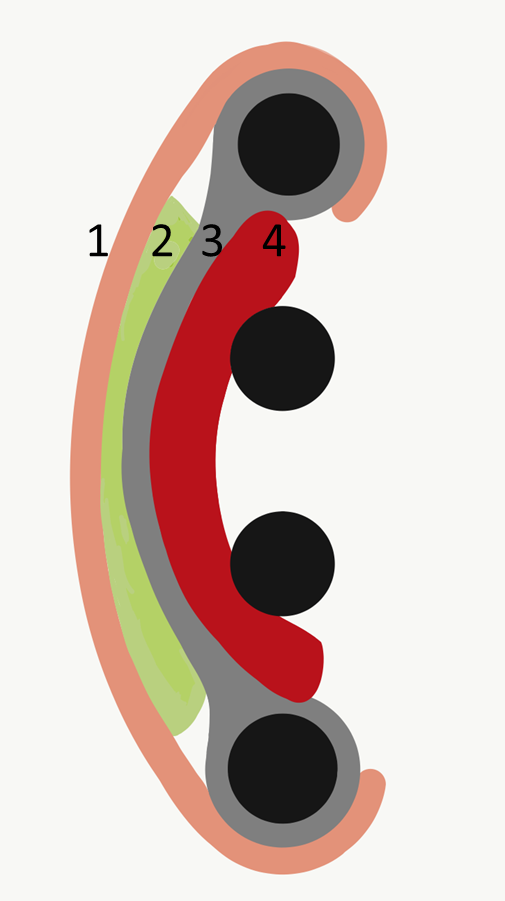
*

| Figure 1. Cross sectional view of chest model tissue layers. From left to right: 1. skin, 2. foam, 3. neoprene, 4. store-bought ribs. Horizontal pipes of the frame are represented by the black circles. *Image author owned (BS)*  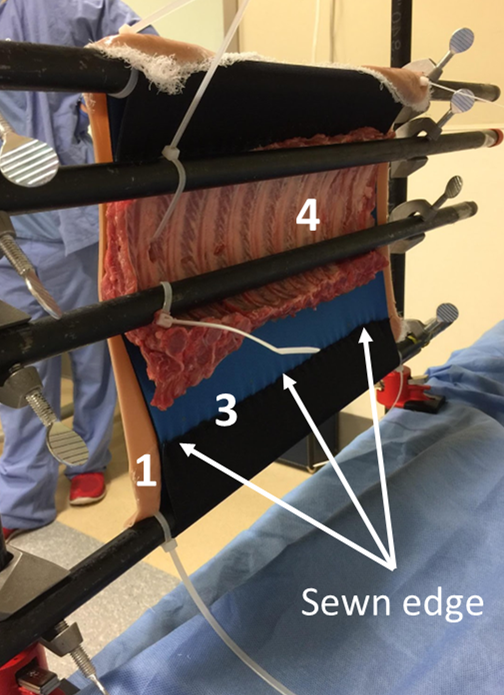 |  |
| --- | --- |

Figure 2. (right) Chest model posterior view revealing tissue layers. From left to right: 1. skin, [2. foam is not visible in this image], 3. neoprene, 4. store-bought ribs. Note neoprene has been folded over and sewn to create a “sleeve” for the lowest pipe in the image. *Image author owned (BS)*


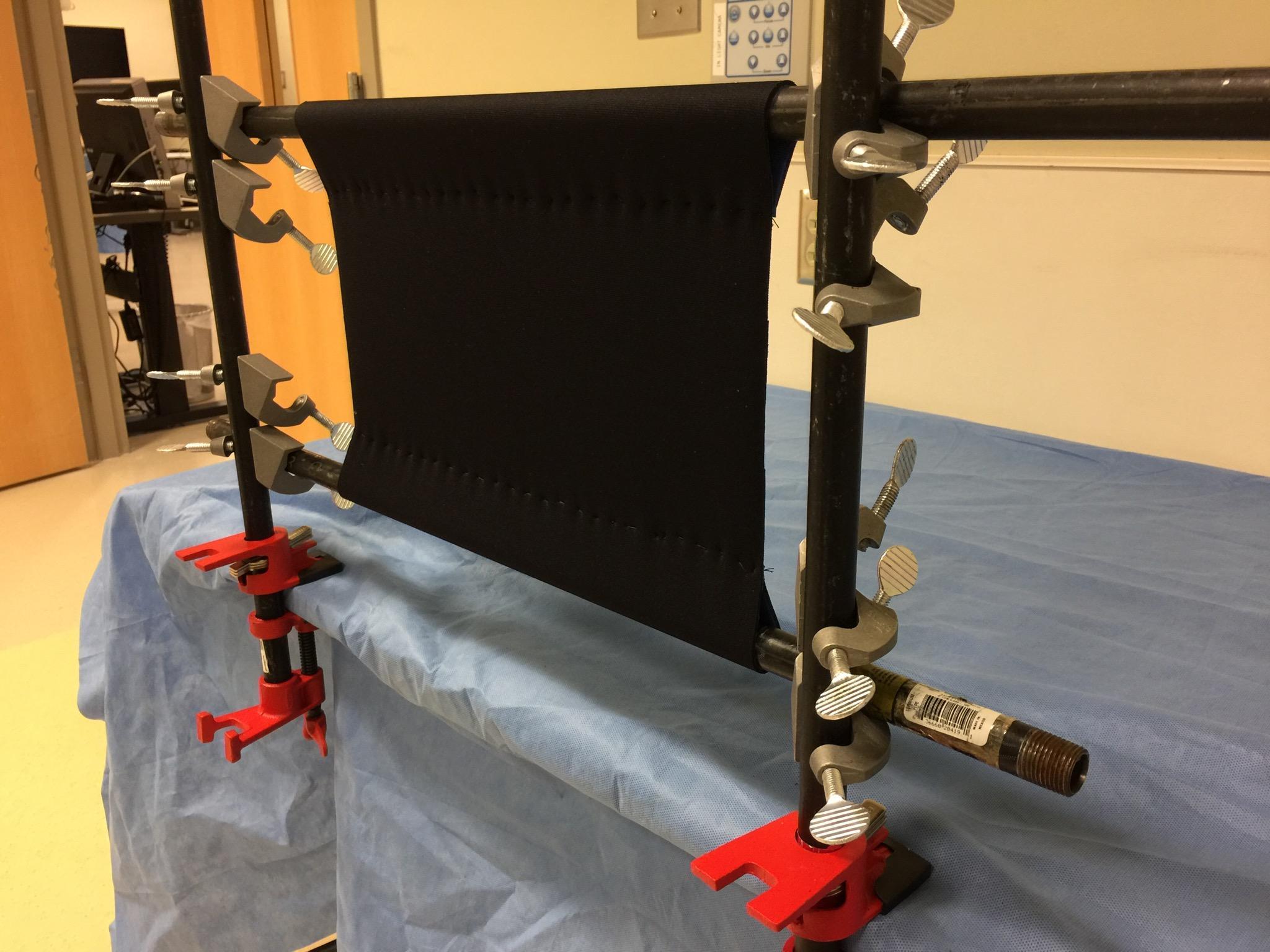


Figure 3. Two ½” pipes are affixed vertically to the table edge with H-style clamp fixtures. Two horizontal pipes are then inserted through the sleeves of the neoprene layer and secured to the vertical pipes with clamps. Neoprene should be taut, but avoid creating too much tension and strain on the sleeve stitching. *Image author owned (BS)*


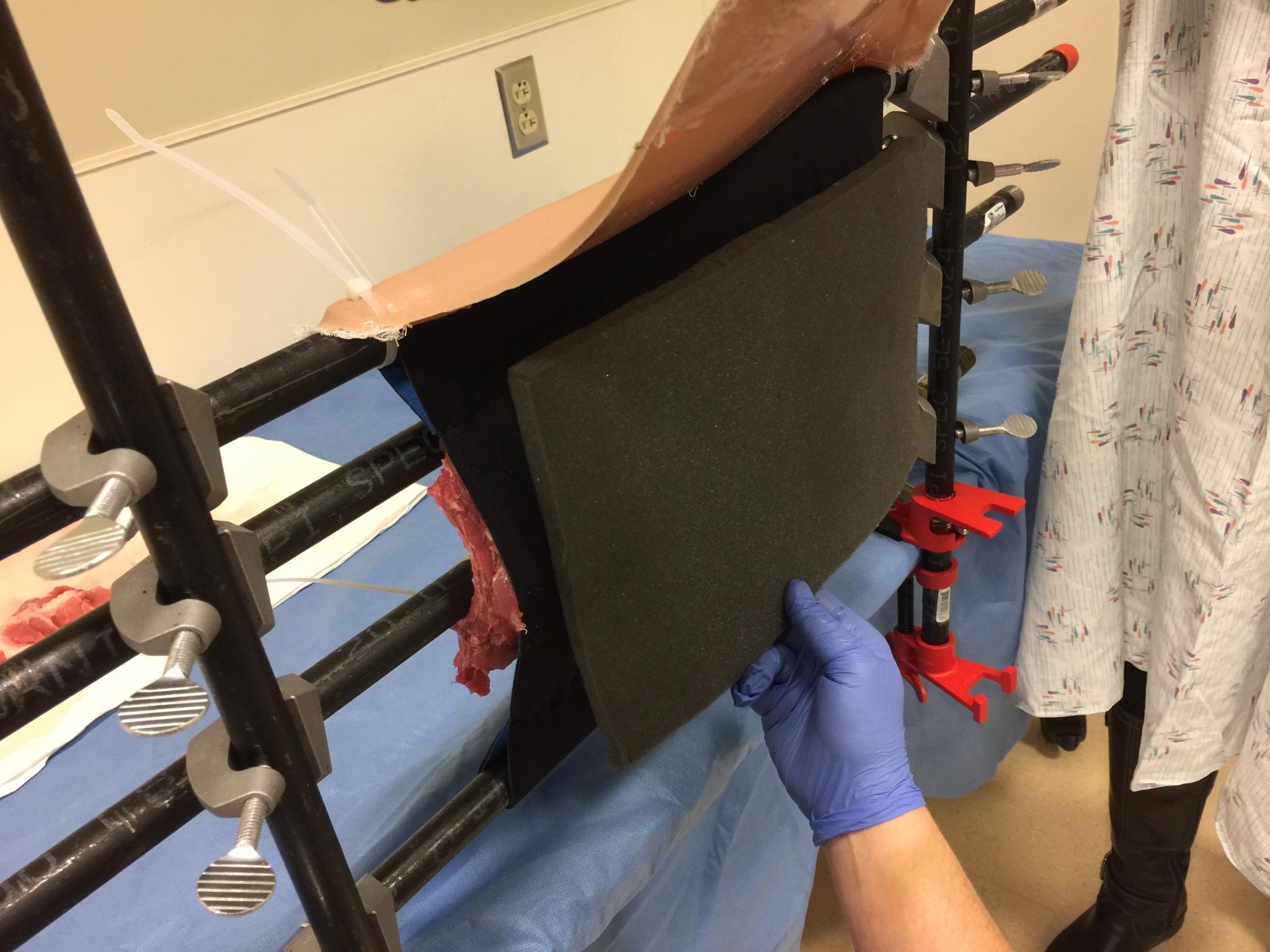


Figure 4. In this view, two additional crossbars have been added, to which the ribs have been fixed with zip ties (see Figure 2). The layer of foam is being placed over the neoprene. The latex skin is attached to the uppermost pipe with zip ties; it will be lowered over the foam and attached to the lower pipe. *Image author owned (BS)*


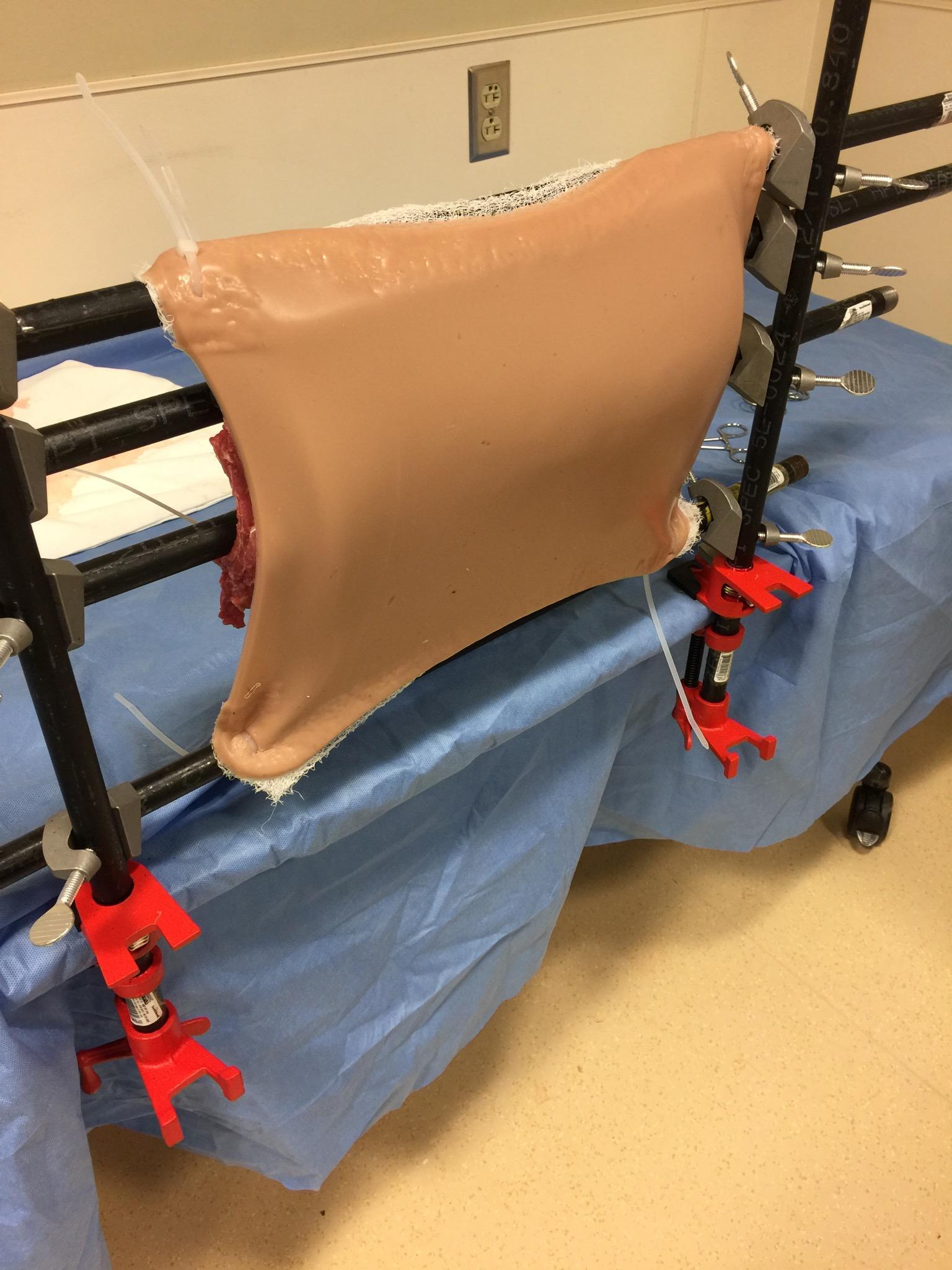


Figure 5. The silicone skin has been lowered over the foam layer and attached to the lower pipe with zip ties. The model is ready for use at this stage, or faculty may wish to affix a 1-liter bag of IV fluids to the ribs by wrapping both with plastic cling wrap, prior to affixing the ribs to the frame (see Appendix D. Instructor Guide Seldinger Chest Tube). If this approach is used, faculty must be prepared to catch a large volume of fluid below the table as the workshop progresses. *Image author owned (BS)*

1. **Additional Resources**

**MAKING SKIN FOR TRAUMA SKILLS IN 10 EASY STEPS:**

Various silicone compounds are available online to make synthetic skins for procedure workshops. The product we used is Eco-Flex 00-20, a two part silicone compound sold in “Part A” and “Part B” sets ([www.smoothon.com](http://www.smoothon.com)). We also used “Silc Pig '' colorant is used to tint the skin; this is not required.

The other **materials** used for making synthetic skins are:

1. Cafeteria trays which were used as the mold.
2. Measuring cups for each part of compound (disposable are best, we used 10 oz plastic disposable cups)
3. Larger container for mixing both parts together.
4. Plastic silverware for mixing and the addition of colorants.
5. Mold release spray is optional depending on the silicone product and mold material used. Various brands are available from art and craft supply stores. We found it was not required for cafeteria trays as they are coated plastic.
6. Gauze for reinforcement of edges. (gauze pads work, or any kind or rolled gauze. We used 1” wide gauze rolls)

To **make** the skins we:

1. Cover our work area with hospital drapes, plastic covering, paper tablecloth, etc.
2. Stir both parts of the compound thoroughly before mixing as there is settlement on the bottom of the containers.
3. Measure equal parts of “A” and “B” together in the larger container.
4. Add a very small amount of ‘Silc Pig” colorant if desired.
5. Mix thoroughly to achieve even consistency and coloration.
6. Spray mold (if applicable)
7. Pour the compound into the cafeteria tray.
8. Shake slightly to settle evenly and remove any possible air bubbles.
9. Lay gauze strips around the outer top and bottom edges. This reinforces the edges of the skin when it is attached to the rack system and pulled taught with zip ties at the top and bottom. Without this reinforcement, the skin may tear.
10. Set molds/trays to cure on a level surface per manufacturer instructions (about 4 hours for the products we use).

The compound should peel out of the tray/mold easily, or can be left in the mold indefinitely.

The skins will keep for a very long time. We stored them between sheets of parchment paper for over a year with no loss in fidelity.

| **Cost Estimates (excludes hospital consumables and surgical instruments)** | | | | | |
| --- | --- | --- | --- | --- | --- |
| **Reusable Items** | **Quantity** | **Price Per Unit** | **Total Price** | **Source** | **Notes** |
| Black pipes 1/2" diameter 48" length | 6 | $12 | $72 | Home Depot Seattle, WA |  |
| Pipe clamp for 1/2" diameter | 2 | $13.50 | $27 | Home Depot Seattle, WA |  |
| Fisherbrand™ Castaloy™ Clamp Jumbo Holder | 8 | $35 | $280 | FisherBrand Scientific |  |
|  |  |  | **$379** |  |  |
| **Non-Reusable Items** | | | | | |
| Pork Ribs | 1 | $12 | $12 | Grocery Store Seattle, WA |  |
| Neoprene 2mm (dimensions TBD) | 1 | $5 | $5 | Fabric Store  Seattle, WA |  |
| Foam 1/4" - 1/2" thickness, 22" length x 7" width | 1 | $2 | $2 | Fabric Store Seattle, WA | One 0.5" x 24" x 90" roll: $27. Yields 12 cuts of 22" x 7." Each cut ~ $2. |
| Zip Ties | Many | $4.50 | $4.50 | Home Depot  Seattle, WA | One package of 20 zip ties: $4.50. At least 20 zip ties per setup, extra is recommended. |
| Smooth On  (EcoFlex 00-20) | N/A | N/A | $4.50 | Amazon.com | One container Smooth On Ecoflex 00-20: $45. Yields 2 pints of liquid skin, which produces at least 10 skin layers.  ~$4.50 per skin. |
|  |  | **Total** | **$28** |  |  |
