# Supplementary material for: A Multidisciplinary Curriculum to Standardize Chest Procedures Training for Trainees in General Surgery, Emergency Medicine, and Critical Care
Source: MedEdPORTAL. 2024 Jul 9;20:11421. doi: 10.15766/mep_2374-8265.11421 (PMC11231065; doi:10.15766/mep_2374-8265.11421)
Supplement: Supplementary file 1 — Surgical Tube Thoracostomy Checklist.docxSample Workshop Schedule.docxInstructor Guide Surgical Chest Tube.docxInstructor Guide Seldinger Chest Tube.docxLow-Cost Chest Tube Model.docxInstructor Guide Chest Tube Securement Station.docxInstructor Guide Thoracentesis.docxInstructor Guide POCUS for Thoracic Procedures.docxThoracic Abnormal US Images.pptxChest Procedures Workshop Evaluation.docx [file mep_2374-8265.11421-s001.zip › H. Instructor Guide POCUS for Thoracic Procedures.docx]

**Point of Care Ultrasound for Thoracic Procedure Planning**

**Instructions: This instructor guide is to be used as a reference by faculty guiding the thoracic ultrasound station. It outlines the supplies needed, station setup, transducer handling and image optimization, normal sonographic anatomy, artifacts, and abnormal findings, and provides a clinical scenario through which to discuss the procedure in a clinical context.**

**
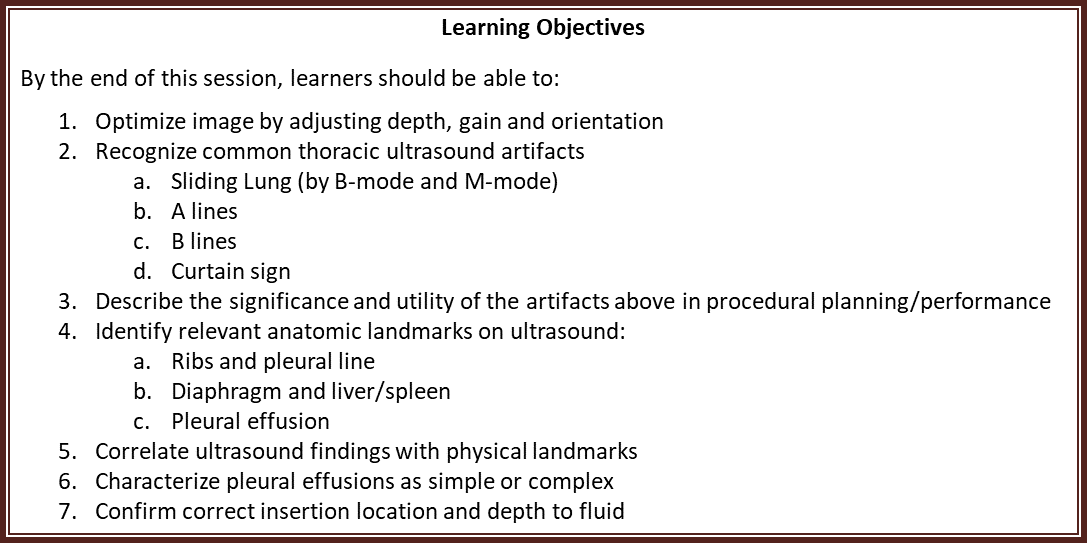
**

**Supplies**

For each group of up to four learners and one instructor:

- Point of care ultrasound machine: one per group
- At minimum, machine should have a sector array transducer (phased array preferred to visualize between ribs, though curvilinear is acceptable)
- Ideally, machine will also have a linear transducer to more clearly delineate pleural line
- Extra gel and towels
- One gurney or chair for model
- One mayo stand or table for laptop
- Laptop with PowerPoint slides loaded (see Appendix I)

Consider starting the session with the clinical case(s) provided at the end of this document.

**Station Setup**

- Learners take turns as models, or instructors can recruit volunteers from outside the learner group prior to the workshop. Model can wear no shirt, sports bra, or T shirt or scrub top
- The model sits on the side of the gurney or chair
- Learners take turns holding the transducer and adjusting machine settings. Instructors should rotate learners frequently so every learner has a chance to use the transducer to obtain images. A good rule of thumb is to rotate learners every 10 minutes
- Please note that some learners may not feel comfortable being models. We make every effort to ensure a comfortable and inclusive learning environment for all, and make it a point not to pressure learners to volunteer as models.

**Prerequisites**

Prior to the workshop learners will be encouraged to watch a video on essential ultrasound physics and the use of ultrasound for thoracentesis planning. The videos we use are open access, available on the Washington Institute for Simulation in Healthcare (WISH) website: <https://wish.washington.edu/online-learning-modules#ultrasound>. While not required, these videos are highly recommended.

Alternatively, instructors may choose to present the didactic material in person at the beginning of the course, using their own slides and videos.

*Required background knowledge*

- Understanding of anatomy and physiology of the chest wall, pleura, and lungs
- Understanding of essential ultrasound physics and artifact interpretation

*Required background skills expected in trainees prior to receiving training in the target course:*

- None

**Step 1: Transducer handling and image optimization (10 minutes)**

During the entirety of the station, instructors should endeavor to NOT touch the ultrasound transducer. Instead, they should guide learners through transducer movements and machine adjustments using verbal instruction.

1. **Transducer handling**

Instructor should ensure learners hold the transducer in a comfortable grip that is not overly tight, stabilized by at least the 5th finger.

1. **Image optimization: Depth**

Ideal depth is at least as deep as the first A-line in a normal thoracic image, or as deep as required to identify relevant anatomy and appreciate the size of a pleural effusion.

1. **Image optimization: Gain**

Ideal gain allows the sonographer to delineate soft tissue planes, visualize A-lines, and determine whether an effusion is simple or complex (discussed in step 4).

1. **Image optimization: Orientation**

Transducer should initially be oriented cephalad-caudad to visualize ribs on either side of the intercostal space. Learners should be able to articulate the utility of the orientation marker on the ultrasound screen to facilitate image interpretation and transducer movement.


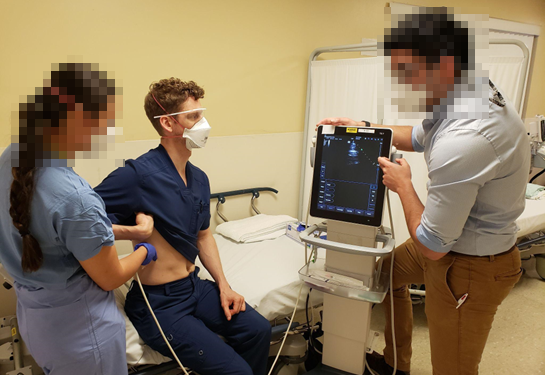


Fig 1. An instructor at the ultrasound station guides a learner through image optimization using a phased array transducer on a normal model. Note this learner and model are not positioned for thoracentesis. Pictured is author RJK, others in image obscured for privacy. *Image author owned (RJK)*

**Step 2: Ultrasound anatomy (5 minutes)**

Learners should be able to identify the following:

- Subcutaneous tissue
- Ribs and rib shadows
- Pleural line
- Diaphragm
- Liver/spleen
- Pleural effusion (see Step 4): Note depth at which operator would expect to aspirate fluid

**Step 3: Normal Ultrasound Artifacts (15 minutes)**

Learners should be able to obtain images, recognize and explain the physics and clinical relevance of:

- Sliding lung sign via B-mode and M-mode (“seashore sign”)
  - Presence confirms apposition of visceral and parietal pleural layers
  - Absence (images of absent sliding lung and M mode with “bar code sign” on PowerPoint slides) does not confirm pneumothorax, but is suspicious if sliding lung was present prior to thoracentesis and afterwards absent, particularly in a symptomatic patient
- A lines
  - Presence indicates normal aerated lung OR pneumothorax deep to pleura
  - Learners should be able to describe the concept of reverberation artifact
  - Instructor should guide learners to fan the transducer to demonstrate how the reverberation artifact can be magnified when ultrasound beams are perpendicular to the pleura (which is not necessarily parallel to the chest wall)
- Curtain sign
  - Presence indicates absence of pleural effusion
  - Learners must consider gravity and placement of transducer to ensure they are adequately posterior (in a supine patient) to rule out pleural effusion

**Step 4: Abnormal findings**

For this step, the instructor should refer to images on the laptop PowerPoint to discuss the following:

- B lines
  - Presence indicates lung parenchyma is not normal, with ultrasound signal penetrating through the pleura due to thickened interstitium or fluid within peripheral alveoli
  - Presence rules out pneumothorax, which can prevent placement of unnecessary chest tube
- Pleural effusion
  - Characterization ranging from simple anechoic to complex, loculated appearance
  - Size and safety of static (as opposed to real-time) ultrasound guidance for small effusions
  - Estimated depth to pleural effusion, for recall during thoracic procedure
  - Color doppler to rule out presence of large vessels in anticipated procedure path
  - Importance of transducer handling to mimic anticipated trajectory of thoracic procedure in estimating depth and assessing presence of vessels

**Clinical Case**

***CC: Altered mentation and dyspnea***

**HPI:** A 48 year old female with a history of alcohol use disorder is brought in by her sister when she was found to be more altered at home with some difficulty breathing after a weekend of heavy drinking. The sister states that the patient does not typically seek medical care and has no known medical problems. The patient is oriented to herself only, but does say she has difficulty breathing and cough. The patient endorses vomiting and malaise. She denies any trauma.

**Allergies:** None

**Meds:** None

**PMHx**: None

**SocHx**: Divorced and lives by herself; per her sister she has had “problems with alcohol” for decades, but she is not sure how much the patient drinks. No tobacco or drug use.

**FamHx:** none

**ROS:** complete ROS negative except as per HPI

***Pertinent PE:***

**Vitals:** Temp 37.6 HR 104, BP 90/60, RR 24, O2 90% on room air

**Gen:** Thin woman in no acute distress

**CV:** Tachycardic, no murmur

**Chest:** Decreased breath sounds 1/2 up the right posterior chest wall with dullness to percussion over the same area. Rhonchi noted above the area of dullness.

**Abd:** Soft, nontender, normal bowel sounds

**Ext/Skin:** jaundice and caput medusa noted

**Chest X-ray:** Right sided pleural effusion with right lower and middle lobe infiltrates concerning for aspiration vs infection.

**Ultrasound:** This case could be used with several of the images included in the accompanying PowerPoint to direct different courses of action by the learners.
