# Supplementary material for: A Multidisciplinary Curriculum to Standardize Chest Procedures Training for Trainees in General Surgery, Emergency Medicine, and Critical Care
Source: MedEdPORTAL. 2024 Jul 9;20:11421. doi: 10.15766/mep_2374-8265.11421 (PMC11231065; doi:10.15766/mep_2374-8265.11421)
Supplement: Supplementary file 1 — Surgical Tube Thoracostomy Checklist.docxSample Workshop Schedule.docxInstructor Guide Surgical Chest Tube.docxInstructor Guide Seldinger Chest Tube.docxLow-Cost Chest Tube Model.docxInstructor Guide Chest Tube Securement Station.docxInstructor Guide Thoracentesis.docxInstructor Guide POCUS for Thoracic Procedures.docxThoracic Abnormal US Images.pptxChest Procedures Workshop Evaluation.docx [file mep_2374-8265.11421-s001.zip › _Educational Summary Report_11421.pdf]

MedEdPORTAL® The AAMC Journal of Teaching and Learning Resources

Original Publication Open Access

A Multidisciplinary Curriculum to Standardize Chest Procedures Training for Trainees in General Surgery, Emergency Medicine, and Critical Care

Jacob LoMonaco, MD*, Robert J. Klemisch, MD, Jonathan S. Ilgen, MD, Beth Sobba, MD, Jenelle Badulak, MD, Thomas K. Varghese Jr, MD, Amy E. Morris, MD

*Corresponding author: jacoblomonaco@gmail.com

Abstract

Introduction: Critical care, emergency medicine, and surgical trainees frequently perform surgical and Seldinger-technique tube thoracostomy, thoracentesis, and thoracic ultrasound. However, approaches to teaching these skills are highly heterogeneous. Over 10 years, we have developed a standardized, multidisciplinary curriculum to teach these procedures. **Methods:** Emergency medicine residents, surgical residents, and critical care fellows, all in the first year of their respective programs, underwent training in surgical and Seldinger chest tube placement and securement, thoracentesis, and thoracic ultrasound. The curriculum included preworkshop instructional videos and 45-minute in-person practice stations (3.5 hours total). Sessions were co-led by faculty from emergency medicine, thoracic surgery, and pulmonary/critical care who performed real-time formative assessment with standardized procedural steps. Postcourse surveys assessed learners' confidence before versus after the workshop in each procedure, learners' evaluations of faculty by station and specialty, and the workshop overall. **Results:** One hundred twenty-three trainees completed course evaluations, demonstrating stable and positive responses from learners of different backgrounds taught by a multidisciplinary group of instructors, as well as statistically significant improvement in learner confidence in each procedure. Over time, we have made incremental changes to our curriculum based on feedback from instructors and learners. **Discussion:** We have developed a unique curriculum designed, revised, and taught by a multidisciplinary faculty over many years to teach a unified approach to the performance of common chest procedures to surgical, emergency medicine, and critical care trainees. Our curriculum can be readily adapted to the needs of institutions that desire a standardized, multidisciplinary approach to thoracic procedural education.

Keywords

Chest Tube, Thoracentesis, Thoracic Ultrasound, Clinical/Procedural Skills Training, Critical Care Medicine, Emergency Medicine, Pulmonary Medicine, Surgery - General, Editor's Choice

Educational Objectives

By the end of this workshop, learners will be able to:

1. Describe the anatomy, landmarks, and insertion locations relevant to common thoracic drainage procedures.
2. Perform surgical tube thoracostomy on a simulated thorax model.
3. Perform Seldinger tube thoracostomy on a simulated thorax model.
4. Secure chest tubes and troubleshoot the connection.
5. Perform thoracentesis on a simulated thorax model.
6. Demonstrate fundamental thoracic ultrasound techniques to plan common thoracic drainage procedures.

Citation:
LoMonaco J, Klemisch RJ, Ilgen JS, et al. A multidisciplinary curriculum to standardize chest procedures training for trainees in general surgery, emergency medicine, and critical care. *MedEdPORTAL*. 2024;20:11421.
https://doi.org/10.15766/mep_2374-8265.11421

Introduction

Chest tube placement, thoracentesis, and thoracic ultrasound are core bedside skills in emergency medicine, general surgery, and critical care medicine practice.[1-4] Simulation provides safe and effective procedural training[5] and can lead to improved patient outcomes.[6,7] However, the ways in which procedures are taught varies by training program, even within the same institution. The variability in training across specialties presents challenges for institutional consistency and shared learning, and past work has demonstrated that both varied teaching methods and a multidisciplinary approach may benefit teaching procedures.[8,9]

Copyright © 2024 LoMonaco et al. This is an open-access publication distributed under the terms of the Creative Commons Attribution license.

Published information regarding multidisciplinary curricula for a wide range of core thoracic procedures is limited. A search of *MedEdPORTAL* for curricula using the terms *chest procedures, chest tube, thoracostomy tube,* or *thoracentesis* did not return any results that either used faculty from multiple disciplines to develop or teach these procedures, were aimed at learners from multiple disciplines, or covered the full range of thoracic procedures in this curriculum. Antonoff, Green, and D'Cunha published a curriculum aiming to teach surgery-bound senior medical students a broad range of intern-level skills including chest tubes, but it does not cover thoracic ultrasound or thoracentesis and is not applicable to a multidisciplinary graduate-level audience.[10] Johnston and colleagues published a hands-on pulmonary device curriculum that addresses thoracostomy tubes in a limited fashion but focuses on general respiratory devices such as incentive spirometry and positive pressure airway devices.[11] Branditz and colleagues published a curriculum on the management of pulmonary emergencies that discusses chest tubes, pigtail chest tubes, and thoracic ultrasound but focuses on broader management of pulmonary emergencies rather than honing the procedural skills of chest tube insertion and thoracic ultrasound.[12] Other resources have provided concise, hands-on means to teaching chest procedures but only in the setting of a broader procedural curriculum and focusing on neonates or general pediatric patients.[13,14] Still others offer a handbook for common inpatient procedures for internal medicine residents but include only thoracentesis among chest procedures.[15]

We have demonstrated exportability within our own institution, as part of this training has been adapted to teach ultrasound and thoracentesis to internal medicine residents and medical students. Our hope is that this curriculum can serve as a plug-and-play solution for institutions seeking a standardized approach to teaching common chest procedures to providers in any specialty.

Methods

In 2009, a multidisciplinary faculty group representing thoracic surgery, emergency medicine, and critical care collaborated to create a uniform training method for surgical tube thoracostomy. Essential didactic elements were identified, and a standardized series of procedural steps was developed (Appendix A) using an informal modified Delphi approach through which experts from all three specialties agreed upon best practices. Over time, this curriculum evolved to encompass the additional common thoracic procedures described here. Our target learners were first-year residents in emergency medicine and general surgery and first-year critical care fellows. Each workshop was led by faculty from all three disciplines, but due to scheduling limitations, the trainees completed the workshop in single-specialty cohorts as a standard component of their protected learning time.

Prior to the workshop, learners were asked to review didactic materials in a flipped classroom approach. These materials included thoracic ultrasound training videos previously published in *MedEdPORTAL,*[16] as well as a module on surgical chest tube placement utilizing images from a published chest tube performance video.[17]

In-person workshops lasted up to 4 hours depending on the number of learners. A brief orientation provided an overview of the schedule (Appendix B). Learners divided into small groups and rotated through five stations (details below). Trainees worked at each station until both they and the instructor felt the trainee had demonstrated competent performance; faculty conducted targeted real-time formative assessment and feedback during each station, utilizing the list of procedural steps included in the station's instructor guide and the checklist in Appendix A.

Station 1: Surgical Tube Thoracostomy (Instructor Guide: Appendix C and Procedure Checklist: Appendix A).
First, the instructor reviewed the anatomic landmarks, then demonstrated and narrated all the steps of the procedure. Trainees were prompted by facilitators to describe the anatomy and procedure and to ask questions. Next, trainees practiced all steps of placing a chest tube, stopping before tube securement. We utilized two sets of equipment so that two trainees could practice simultaneously on each side of a TraumaMan manikin (Simulab Corporation). Learners verbalized positioning the patient and using universal barrier precautions. They described anatomic landmarks and insertion location before pantomiming performance of local anesthesia. They then practiced incising the skin, dissecting through the intercostal muscles, and inserting the chest tube.

Learners alternated between performance and observation as faculty guided the group to discuss prevention of potential complications such as incorrect insertion site, failure to ensure intrapleural placement, and suboptimal tube placement (e.g., within a fissure).

Station 2: Seldinger-Technique Tube Thoracostomy (Instructor Guide: Appendix D)
This station used a novel, low-cost, high-fidelity simulator that securely suspended animal ribs such that their orientation simulated a supine patient (Appendix E).

Copyright © 2024 LoMonaco et al. This is an open-access publication distributed under the terms of the Creative Commons Attribution license.

First, trainees discussed patient positioning, identifying the correct rib space, and using ultrasound to confirm effusion findings. They pantomimed cleaning the skin and draping the patient. They then practiced the procedure on the simulator, alternating between performance and observation as the group discussed potential complications and their prevention.

Station 3: Chest Tube Securement, Attachment to Collection Device, and Troubleshooting (Instructor Guide: Appendix F)
This station started with a thoracic-procedure manikin with surgical chest tubes already inserted. First, learners practiced suturing the tubes to the chest wall. Next, they set up and secured the chest tube to the collection system. They then demonstrated stepwise assessment of the entire drainage system, including inspection for tidaling and troubleshooting air leaks.

Station 4: Thoracentesis (Instructor Guide: Appendix G)
This station began with a discussion of indications, contraindications, and patient positioning. Instructors oriented trainees to the contents of a standard thoracentesis/paracentesis kit used at our institution.

Next, trainees identified fluid in an ultrasound-compatible partial task trainer and discussed appropriate insertion location. In order to reduce waste, they pantomimed donning sterile attire, prepping and draping the patient, and placing a sterile ultrasound probe cover. Finally, they performed a complete thoracentesis, including tubing connection and demonstrating proper drainage technique, while the group discussed potential complications and their prevention.

Station 5: Thoracic Ultrasound for Procedural Planning (Instructor Guide: Appendix H)
First, trainees used each other as models to identify and explain the etiology and significance of key ultrasound findings such as lung sliding, A-lines, and the M-mode "seashore sign."

Next, they reviewed abnormal findings in a standard set of video clips: absent lung sliding, M-mode "barcode sign," lung point, B lines, and pleural effusion of various sizes and degrees of complexity (Appendix I). Faculty guided learners through interpretation of various combinations of these findings to aid in procedural decision-making and planning.

Optional Curriculum Expansion Activities
Our workshops focused heavily on the hands-on skills at each station as described above. However, the instructor guides (Appendices C, D, F-H) include clinical scenarios that can be used to augment decision-making discussions either at the relevant hands-on station or, for larger learner groups, at separate discussion stations.

Assessment of Achievement of Learning Objectives
Learners reviewed the anatomy, landmarks, and insertion locations relevant to common thoracic drainage procedures before the workshop, as well as in person during the surgical chest tube and thoracic ultrasound stations, in line with Educational Objective 1. They performed and practiced placement of surgical chest tubes (Educational Objective 2) and Seldinger chest tubes (Educational Objective 3), securement of chest tubes and tubing troubleshooting (Educational Objective 4), thoracentesis (Educational Objective 5), and fundamentals of thoracic ultrasound (Educational Objective 6) with instructors conducting real-time formative assessment utilizing standardized lists of procedural skills included in each instructor guide. Formal summative assessment was not routinely employed due to time constraints and the large numbers of trainees at each workshop, in favor of maximizing the time for learners to practice at their own pace and discuss technique with the instructor. However, the surgical tube thoracostomy checklist available in Appendix A was structured for use as a summative assessment tool if desired.

After the workshop, learner surveys (Appendix J) were distributed. For the first 4 years of the curriculum, learners completed paper surveys, a preprocedure confidence assessment, and a final evaluation prior to leaving the workshop. Later in course development, surveys were transitioned to an online postworkshop format to avoid survey fatigue, and learners were able to complete evaluations remotely. In all formats, surveys were collected anonymously. Data points obtained included trainees' global evaluation of the course and individual stations, faculty performance at each station, and trainees' self-assessed confidence in performing each procedure before versus after the workshop. An overall course review question was not asked until 2015, so, for surveys collected prior to this, we used responses to the prompt "The course enhanced my understanding of surgical chest tubes" as this station was at the time the majority of the course. The chest tube securement station did not have individual evaluation data as it was added later in the course evolution. Given that the first 4 years of the course used preworkshop surveys of preworkshop procedural confidence and that this was later changed to postworkshop surveys alone, the preworkshop survey cohort was compared to the postworkshop survey cohort to assess for differences in preworkshop confidence by procedure.

Copyright © 2024 LoMonaco et al. This is an open-access publication distributed under the terms of the Creative Commons Attribution license.

All measurements utilized 5-point Likert scales. Anchors for learner assessment of the course and faculty were 1 (*strongly disagree*) to 5 (*strongly agree*). Average scores and confidence intervals were determined, analyzed by trainee specialty, and compared against each other using a one-way analysis of variance (ANOVA). Anchors for self-assessment questions were 0 (*not at all confident* [in ability to perform this procedure]) to 5 (*very confident*). Learners' responses before and after the workshop were compared respectively to each station using a two-sample paired *t* test for means. Finally, learner evaluations of faculty from their own specialty versus faculty from other specialties were compared using a two-sample *t* test assuming unequal variances.

Learner Feedback and Workshop Evolution

Over time, we have incrementally changed our curriculum structure and content in response to faculty and learner feedback. For example, early offerings of this course included only one chest tube station. However, tube securement and system troubleshooting were identified as important and time-intensive components of competency. Trainees routinely had questions on these steps, and securement had been specifically addressed in prior procedural education research given the risk of dislodgement.[18] We therefore created a dedicated station for practicing these skills. Similarly, Seldinger tube thoracostomy kits were not widely available when this workshop was first developed, and point-of-care ultrasound was not yet routinely used. These stations were developed as clinical practice evolved and are now part of the core curriculum for all trainees.

Results

Since inception, we have received surveys from 123 learners representing all three groups of trainees. Overall course ratings (scored on a 5-point scale where 1 = *highly unfavorable,* 5 = *highly favorable*) for all groups of learners have been consistently excellent (Figure 1). Learners rated their confidence in performing each procedure before versus after the workshop, with statistically significant improvement in these self-reported measures in all categories (Figure 2). Learners felt faculty of all stations were competent and advanced their learning (Figure 3). Lastly, learners rated station faculty from their own specialty similarly to other specialties (Figure 4), underscoring the acceptability of this multidisciplinary approach.

Overall Course Evaluation

There were 25 survey responses from the emergency medicine trainees, who rated the overall course an average of 4.6 (95% CI, 4.4 to 4.9). For the 44 surveys from surgery trainees, the average

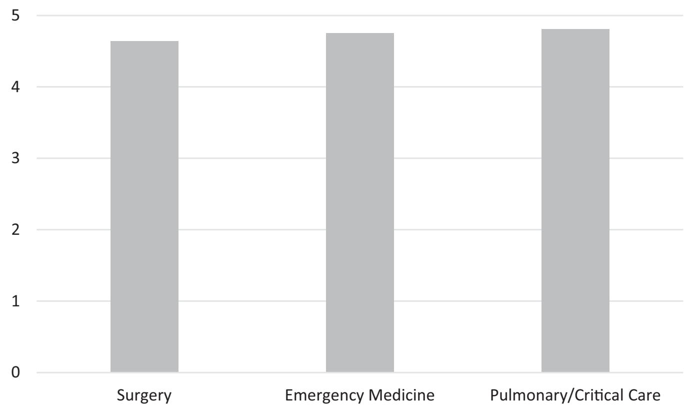

Figure 1. Overall course rating by trainee specialty, rated on a 5-point scale (1 = *highly unfavorable,* 5 = *highly favorable*). An overall course evaluation question was not asked until 2015, so these responses were combined with responses to the prompt "The course enhanced my understanding of surgical chest tubes" from 2009 to 2015 as this station was at the time the majority of the course. All trainee groups rated the course highly, and differences between groups are not statistically significant.

response was 4.8 (95% CI, 4.6 to 4.9). In the pulmonary/critical care group, 54 responses were received, with an average rating of 4.8 (95% CI, 4.7 to 4.9). Comparing these responses via one-way ANOVA, the differences were not statistically significant ($p = .38$). Overall course ratings are presented in Figure 1.

Improvement in Trainee Confidence After the Workshop

After each workshop, we collected attitudinal survey responses in which learners were asked to rate their confidence in their ability to perform each procedure on a 5-point scale (1 = *not at all confident,* 5 = *very confident*). For the surgical chest tube station, 92 surveys were collected. Analysis was carried out using two-sample paired *t* tests for means. Learners rated their confidence in their ability to perform surgical tube thoracostomy at an average 2.7 before the workshop and 4.1 after, for an average improvement of 1.4 (95% CI, 1.2 to 2.9). For Seldinger chest tubes ($n = 55$), average confidence before the workshop was 1.8, improving to 3.7 after the workshop, for an average improvement of 1.9 (95% CI, 1.6 to 2.1). For thoracentesis ($n = 59$), average confidence increased from 2.2 to 3.9, for an improvement of 1.7 (95% CI, 1.5 to 2.0). For the thoracic ultrasound ($n = 27$), confidence improved from 1.8 to 3.6, for an average increase of 1.8 (95% CI, 1.4 to 2.2). Changes in learner confidence before versus after the course are depicted in Figure 2.

The cohort that completed precourse surveys on procedural confidence was compared to the later cohort that completed postcourse surveys alone to assess preworkshop confidence by procedure. There were no statistically significant differences between these groups. For the surgical chest tube station, the

Copyright © 2024 LoMonaco et al. This is an open-access publication distributed under the terms of the Creative Commons Attribution license.

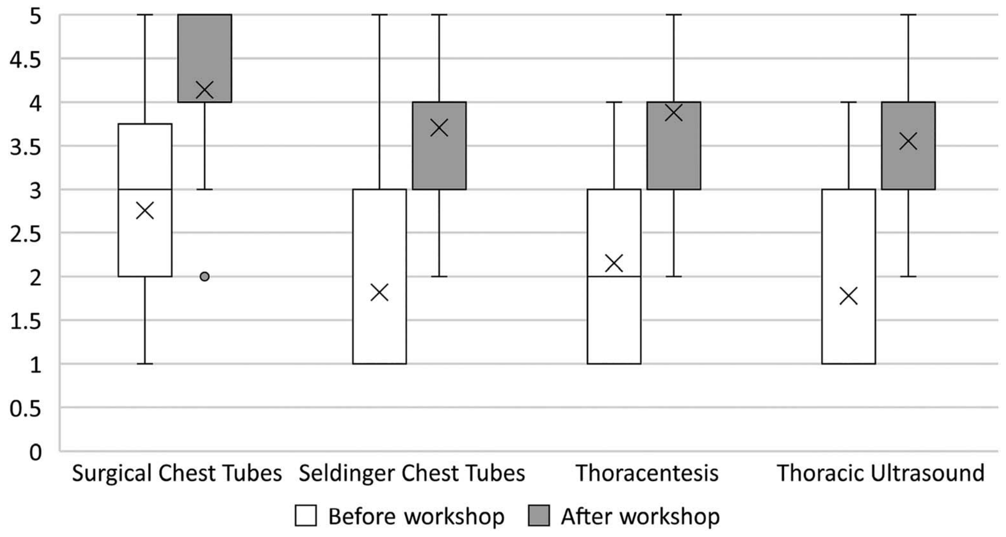

Figure 2. Box and whisker plots of learner confidence by procedure before and after the workshop, rated on a 5-point scale (1 = *not at all confident,* 5 = *very confident*). Improvement was statistically significant for all categories. Upper and lower whisker lines signal the maximum and minimum of the data, boxes represent the interquartile range, and Xs represent the mean of the data. The open circle indicates an outlier. A horizontal line inside a box indicates the median line, where one exists.

mean difference was 0.4 (95% CI, −0.1 to 0.9). For the Seldinger chest tube station, the mean difference was 0.4 (95% CI, −1.0 to 0.2). For the thoracentesis station, the difference was 0.4 (95% CI, −1.0 to 0.2). The thoracic ultrasound station was added later in course development, so it has been omitted from this analysis.

Learner Evaluation of Faculty

Trainees were asked to rate their instructing faculty members on two survey items, “The instructor had good command of the content” and “The instructor contributed to my learning.” Both items were scored from 1 (*strongly disagree*) to 5 (*strongly agree*).

In response to the prompt “The instructor had good command of the content,” the surgical chest tube station surveys (n = 81) recorded an average response of 4.8 (95% CI, 4.7 to 4.9). Other station average scores were as follows: Seldinger tube thoracostomy (n = 54), 4.8 (95% CI, 4.7 to 4.9); thoracentesis (n =

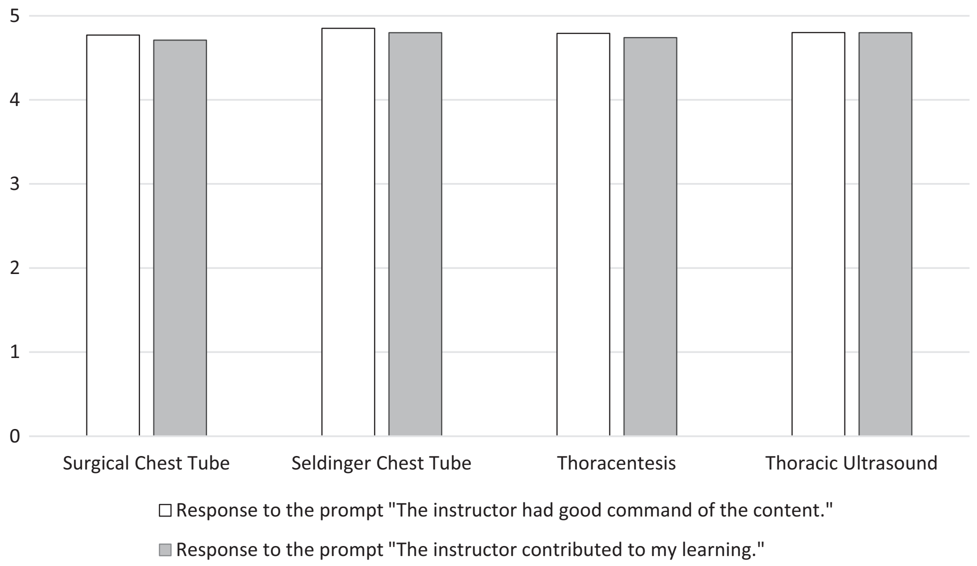

Figure 3. Learners’ evaluation of instructor competence and contribution to their learning by station, rated on a 5-point scale (1 = *strongly disagree,* 5 = *strongly agree*).

Copyright © 2024 LoMonaco et al. This is an open-access publication distributed under the terms of the Creative Commons Attribution license.

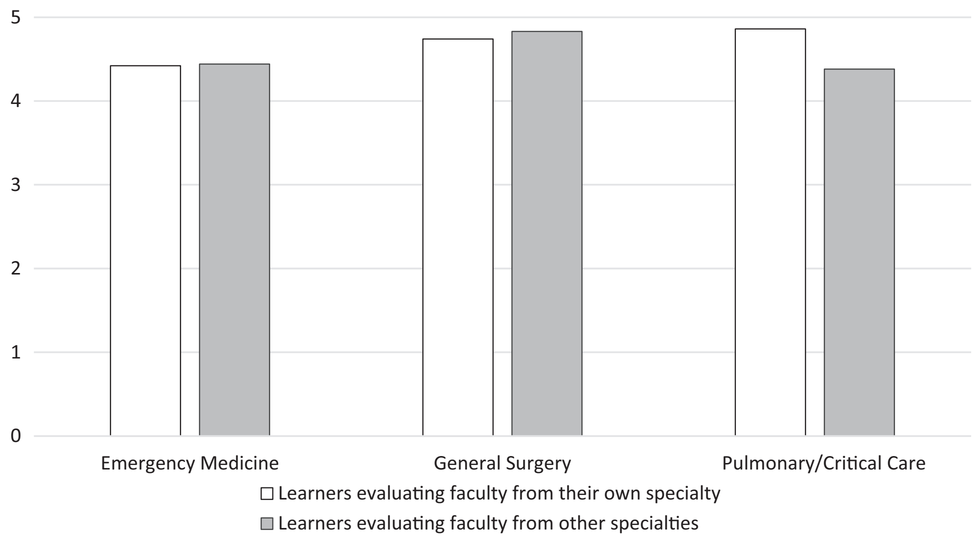

Figure 4. Learner evaluations of instructors from their own specialties versus other specialties. Learners rated the prompt "Overall, the instructor contributed to my learning" on a 5-point scale (1 = *strongly disagree,* 5 = *strongly agree*). There were no statistically significant differences between trainees' evaluations of faculty from their own versus other specialties. There was a nonsignificant but consistent trend of learners rating instructors from other specialties higher than their own.

42), 4.8 (95% CI, 4.7 to 4.9); and thoracic ultrasound ($n = 46$), 4.8 (95% CI, 4.7 to 4.9). One-way ANOVA testing of the means between the groups showed no significant difference ($p = .69$).

In response to the survey prompt "The instructor contributed to my learning," the surgical chest tube station had 80 responses, with an average of 4.7 (95% CI, 4.6 to 4.8). Other station average scores were as follows: Seldinger tube thoracostomy, 4.8 (95% CI, 4.7 to 4.9); thoracentesis, 4.7 (95% CI, 4.6 to 4.9); and thoracic ultrasound, 4.8 (95% CI, 4.7 to 4.9). One-way ANOVA carried out between groups also showed no significant differences for this question ($p = .61$). Trainee evaluations of faculty are depicted in Figure 3.

Learner Evaluations of Faculty From Their Own Versus Other Specialties

Emergency medicine trainee survey responses included 12 evaluations of emergency medicine faculty, with an average score of 4.4 (95% CI, 4.1 to 4.7), and 18 evaluations of faculty from other specialties, with an average score of 4.4 (95% CI, 4.2 to 4.7). The differences between emergency medicine trainee evaluations of their own versus other faculty were not statistically significant (95% CI, −0.4 to 0.4).

Surgery trainee survey responses included 62 evaluations of their own faculty, with an average score of 4.7 (95% CI, 4.6 to 4.8), and 95 evaluations of faculty from other specialties, with an average score of 4.8 (95% CI, 4.7 to 4.9). These differences were also not statistically significant (95% CI, −0.1 to 0.0).

Pulmonary and critical care trainee survey responses included seven evaluations of their own faculty, with an average score of 4.9 (95% CI, 4.5 to 5.0), and eight evaluations of faculty from other departments, with an average of 4.4 (95% CI, 2.9 to 5.0). These differences were also not statistically significant (95% CI, −1.0 to 2.0). Trainees evaluating their own versus other specialties' faculty are depicted in Figure 4.

Discussion

We have developed a curriculum that meets a common need among multiple medical specialties, merging the expertise and best practices of each to standardize thoracic drainage procedures and ultrasound training for a broad range of trainees. Existing literature on curricula that both address the full range of core chest procedures and are taught by, and applicable to, a wide range of specialties is limited. Our curriculum is unique in its development and execution by faculty from multiple specialties to ensure standardized training for trainees of all disciplines. Multidisciplinary collaboration in thoracic procedures allows faculty and trainees alike to benefit from colleagues' expertise and ensures patients throughout the hospital receive excellent care no matter the specialty of the provider performing a procedure. Much as interprofessional collaboration benefits patients by reducing length of stay and improving adherence to recommended practices in a variety of aspects of clinical care, shared expectations around procedural competence

Copyright © 2024 LoMonaco et al. This is an open-access publication distributed under the terms of the Creative Commons Attribution license.

have the potential to improve care transitions between services.[19]

Our learners uniformly rated the multidisciplinary approach highly. Each group of trainees rated instructors from other specialties at least as high as those from their own. These findings suggest multidisciplinary instruction is effective; anecdotally, we have found that this sharing of expertise between specialties has built respect between disciplines, which has been particularly beneficial for our trainees as they form their professional identity. We feel this is a highlight of the curriculum. Our findings suggest that multidisciplinary instruction is also effective and even desirable for more advanced trainees such as critical care fellows. Trainees rated their learner confidence in performing each procedure significantly higher at the end of the workshop.

Our curriculum not only reduces training variability but also has been sustainable for over 10 years with stable and positive course ratings. These ratings likely reflect in part the incremental changes made over time to mirror changing clinical practice, including the rising importance of small-bore chest tubes placed via the Seldinger technique [20-22] and the shift toward point-of-care ultrasound as the standard of care in procedural planning.[23] In contrast to this expansion of procedural skills over time, we have also noted a decrease in the number of thoracenteses performed during residency by incoming pulmonary fellows, which reflects larger trends in internal medicine practice.[24] Ten years ago, we did not include this station in the fellows' chest procedures workshop but have done so in recent years to address this emergent training gap. Given its modular nature, our curriculum can be easily tailored to the needs of a given program or institution with varying learner backgrounds as is evident from our success implementing the curriculum for both residents and fellows as well as multiple specialties. The modular nature also allows for individual components (e.g., surgical chest tubes, thoracic ultrasound) to be selected or removed to better fit the needs of an institution. These incremental changes have been a large part of the sustainability and durability of the curriculum across our institution, and this flexibility supports its adaptation to other institutions.

Our curriculum has some limitations. Though it has been used over many years in multiple departments, it has not yet been adopted at other institutions. While we have tried to include options for lower-fidelity equipment, we recognize that some of the curriculum would be difficult to replicate in a lower-resource setting. Regarding assessment, asking learners about preprocedure confidence after the workshop carries some risk of bias. More importantly, it must be emphasized that learner confidence (Kirkpatrick level 2, learning) is not a surrogate for competence (Kirkpatrick levels 3-4, behavior, results).[25] Faculty in our workshops conduct real-time formative assessment of trainees' ability to correctly perform all procedural steps and provide targeted feedback using a standardized list of procedural steps. However, time constraints did not allow our large number of learners to undergo formal summative evaluation at dedicated testing stations, which would increase evidence of curricular efficacy. The procedural steps in each instructor guide and especially the itemized checklist we developed for surgical tube thoracostomy (Appendix A) would be particularly suited to this purpose. Alternatively, assessment tools for chest tube insertion published after our curriculum had been developed, such as the modified Objective Structured Assessment of Technical Skills, have additional validity evidence and could be incorporated.[26] Our survey method changed after the first 4 years of the curriculum, transitioning from pre- and postcourse surveys to postcourse surveys only, which could affect how trainees have rated their preworkshop confidence. However, our subgroup analysis did not find any statistically significant differences between these groups.

In summary, we have created a unique multidisciplinary curriculum to address common chest drainage procedures and the thoracic ultrasound techniques needed for their performance. We have standardized the training using a multispecialty group of faculty and applied it to trainees across a range of specialties. Our feedback has been consistently positive, and we have made iterative improvements over a decade of use. We feel that this curriculum can benefit other institutions seeking to standardize the training of common chest procedures across disciplines.

Appendices

A. Surgical Tube Thoracostomy Checklist.docx
B. Sample Workshop Schedule.docx
C. Instructor Guide Surgical Chest Tube.docx
D. Instructor Guide Seldinger Chest Tube.docx
E. Low-Cost Chest Tube Model.docx
F. Instructor Guide Chest Tube Securement Station.docx
G. Instructor Guide Thoracentesis.docx

Copyright © 2024 LoMonaco et al. This is an open-access publication distributed under the terms of the Creative Commons Attribution license.

H. Instructor Guide POCUS for Thoracic Procedures.docx

I. Thoracic Abnormal US Images.pptx

J. Chest Procedures Workshop Evaluation.docx

All appendices are peer reviewed as integral parts of the Original Publication.

Jacob LoMonaco, MD: Third-Year Resident, Department of Emergency Medicine, Kirk Kerkorian School of Medicine at the University of Nevada, Las Vegas; ORCID: https://orcid.org/0000-0002-6635-3824

Robert J. Klemisch, MD: Acting Instructor, Department of Emergency Medicine, University of Washington School of Medicine

Jonathan S. Ilgen, MD: Professor, Department of Emergency Medicine, University of Washington School of Medicine

Beth Sobba, MD: Third-Year Resident, Department of Obstetrics and Gynecology, Ochsner Clinic Foundation

Jenelle Badulak, MD: Assistant Professor, Department of Emergency Medicine, University of Washington School of Medicine

Thomas K. Varghese Jr, MD: Professor, Department of Surgery, University of Utah School of Medicine

Amy E. Morris, MD: Associate Professor, Department of Medicine, University of Washington School of Medicine

Disclosures
None to report.

Funding/Support
None to report.

Ethical Approval
Reported as not applicable.

References

1. Havelock T, Teoh R, Laws D, Gleeson F; BTS Pleural Disease Guideline Group. Pleural procedures and thoracic ultrasound: British Thoracic Society pleural disease guideline 2010. *Thorax*. 2010;65(suppl 2):i61-i76. https://doi.org/10.1136/thx.2010.137026
2. *Supplemental Guide: Pulmonary Critical Care*. Accreditation Council for Graduate Medical Education; 2020. Accessed June 7, 2024. https://www.acgme.org/globalassets/pdfs/milestones/pulmonarycriticalcaresupplementalguidetemplate.docx
3. *Supplemental Guide: Surgery*. Accreditation Council for Graduate Medical Education; 2019. Accessed June 7, 2024. https://www.acgme.org/globalassets/pdfs/milestones/surgerysupplementalguide.pdf
4. Beeson MS, Ankel F, Bhat R, et al; 2019 EM Model Review Task Force; American Board of Emergency Medicine. The 2019 Model of the Clinical Practice of Emergency Medicine. *J Emerg Med.* 2020;59(1):96-120. https://doi.org/10.1016/j.jemermed.2020.03.018
5. Tan TX, Buchanan P, Quattromani E. Teaching residents chest tubes: simulation task trainer or cadaver model? *Emerg Med Int*. 2018:9179042. https://doi.org/10.1155/2018/9179042
6. Barsuk JH, Cohen ER, Williams MV, et al. Simulation-based mastery learning for thoracentesis skills improves patient outcomes: a randomized trial. *Acad Med*. 2018;93(5):729-735. https://doi.org/10.1097/ACM.0000000000001965
7. Seam N, Lee AJ, Vennero M, Emlet L. Simulation training in the ICU. *Chest*. 2019;156(6):1223-1233. https://doi.org/10.1016/j.chest.2019.07.011
8. Vogel D, Harendza S. Basic practical skills teaching and learning in undergraduate medical education—a review on methodological evidence. *GMS J Med Educ*. 2016;33(4):Doc64. https://doi.org/10.3205/zma001063
9. Ritter KA, Horne C, Nassar A, French JC, Prabhu AS, Lipman JM. Multidisciplinary simulation training improves surgical resident comfort with airway management. *J Surg Res*. 2020;252:57-62. https://doi.org/10.1016/j.jss.2020.02.008
10. Antonoff M, Green C, D'Cunha J. Operative and technical skills for the senior medical student entering surgery. *MedEdPORTAL*. 2013;9:9470. https://doi.org/10.15766/mep_2374-8265.9470
11. Johnston M, Bridges T, Palen B, Parsons E, Wemple M, Adamson R. Hands-on pulmonary curriculum: interactive learning sessions on oxygen delivery, spirometry, positive airway pressure devices, tracheostomy, and thoracostomy tubes. *MedEdPORTAL*. 2017;13:10633. https://doi.org/10.15766/mep_2374-8265.10633
12. Branditz L, King A, Kaide C, et al. Emergency medicine curriculum utilizing the flipped classroom method: pulmonary emergencies. *J Educ Teach Emerg Med*. 2019;4(4):1-72. https://doi.org/10.5070/M544045605
13. Rosen O, Campbell D, Bruno C, Gabelman L, Goffman D, Angert R. Low-cost, easy-to-assemble neonatal procedural trainers: chest tube, pericardiocentesis, and exchange transfusion. *MedEdPORTAL*. 2014;10:9787. https://doi.org/10.15766/mep_2374-8265.9787
14. Nestor-Arjun B, Miano M. Orientation to emergency pediatric procedures. *MedEdPORTAL*. 2014;10:9852. https://doi.org/10.15766/mep_2374-8265.9852
15. Abraham J, Al-Aloul B, Ali SS, et al. Introduction to inpatient procedures: a resident-to-resident guide. *MedEdPORTAL*. 2008;4:641. https://doi.org/10.15766/mep_2374-8265.641
16. Vande Vusse L, Shepherd A, Bergam B, Andros J, Morris A. Procedure training workshop for internal medicine residents that emphasizes procedural ultrasound: logistics and teaching

Copyright © 2024 LoMonaco et al. This is an open-access publication distributed under the terms of the Creative Commons Attribution license.

materials. *MedEdPORTAL*. 2020;16:10897. https://doi.org/10.15766/mep_2374-8265.10897

17. Dev SP, Nascimiento B Jr, Simone C, Chien V. Chest-tube insertion. *N Engl J Med*. 2007;357(15):e15. https://doi.org/10.1056/NEJMvcm071974
18. Ruparel RK, Laack TA, Brahmbhatt RD, et al. Securing a chest tube properly: a simple framework for teaching emergency medicine residents and assessing their technical abilities. *J Emerg Med*. 2017;53(1):110-115. https://doi.org/10.1016/j.jemermed.2017.02.016
19. Reeves S, Pelone F, Harrison R, Goldman J, Zwarenstein M. Interprofessional collaboration to improve professional practice and healthcare outcomes. *Cochrane Database Syst Rev*. 2017;(6):CD000072. https://doi.org/10.1002/14651858.CD000072.pub3
20. Filosso PL, Sandri A, Guerrera F, et al. When size matters: changing opinion in the management of pleural space—the rise of small-bore pleural catheters. *J Thorac Dis*. 2016;8(7): E503-E510. https://doi.org/10.21037/jtd.2016.06.25
21. Light RW. Pleural controversy: optimal chest tube size for drainage. *Respirology*. 2011;16(2):244-248. https://doi.org/10.1111/j.1440-1843.2010.01913.x
22. DeVivo A. Unlocking common ED procedures—cutting through the triangle: a review of tube thoracostomy in the ED. *emDocs* blog. June 20, 2019. Accessed June 7, 2024. http://www.emdocs.net/unlocking-common-ed-procedures-cutting-through-the-triangle-a-review-of-tube-thoracostomy-in-the-ed/
23. Menegozzo CAM, Utiyama EM. Steering the wheel towards the standard of care: proposal of a step-by-step ultrasound-guided emergency chest tube drainage and literature review. *Int J Surg*. 2018;56:315-319. https://doi.org/10.1016/j.ijsu.2018.07.002
24. Wigton RS, Alguire P. The declining number and variety of procedures done by general internists: a resurvey of members of the American College of Physicians. *Ann Intern Med*. 2007; 146(5):355-360. https://doi.org/10.7326/0003-4819-146-5-200703060-00007
25. Kirkpatrick DL. Techniques for evaluating training programs. *J Am Soc Train Dir*. 1959;13(11):3-9.
26. Friedrich M, Ober J, Haubruck P, et al. Pilot evaluation of an Objective Structured Assessment of Technical Skills tool for chest tube insertion. *GMS J Med Educ*. 2018;35(4):Doc48. https://doi.org/10.3205/zma001194

Received: May 19, 2023
Accepted: March 19, 2024
Published: July 9, 2024

Copyright © 2024 LoMonaco et al. This is an open-access publication distributed under the terms of the Creative Commons Attribution license.
